# Decarbonising concrete production in the United Kingdom: a life cycle assessment of energy, transport, and fuel transitions

**DOI:** 10.1007/s11367-025-02537-5

**Published:** 2025-10-06

**Authors:** Irene Josa, Aiduan Borrion

**Affiliations:** 1 The Bartlett School of Sustainable Construction, University College London, London, UK; 2 Department of Civil, Environmental & Geomatic Engineering, University College London, London, UK

**Keywords:** Sustainable construction, Life cycle assessment (LCA), Environmental impact assessment, Alternative fuels, Built environment, Carbon footprint

## Abstract

**Purpose:**

The construction sector, and in particular concrete, contributes substantially to global emissions, energy demand, and the extensive use of materials. To address these challenges, it is important to develop and implement strategies that reduce the environmental footprint of concrete supply chains. Understanding such impacts and the ways to mitigate them is essential. Therefore, this study focuses on analysing the impacts of decarbonisation strategies within the construction sector, with a specific focus on concrete.

**Methods:**

A life cycle assessment (LCA) was conducted at different levels of the concrete supply chain, from the production in the United Kingdom (UK) of 1 ton of cement and 1 m^3^ of concrete to the construction of a building. In addition to the business-as-usual scenario, three alternative scenarios were assessed, namely cleaner electricity, in which the impact of using five different electricity grid mixes was evaluated; cleaner transportation, for which the impact of using battery-powered electric trucks and different transportation distances was assessed; and cleaner fuels, for which the impact of using alternative fuel combinations in the cement kiln was analysed. Multi-objective optimisation was used to find the optimal solution when minimising Global Warming Potential (GWP) and maximising the reduction of all the other impact categories.

**Results and discussion:**

The results show that significant reductions (of 10 to 37%) in CO_2_-eq emissions can be achieved when combining different strategies. However, certain strategies could bring an increase in other impact categories, including stratospheric ozone depletion, ionising radiation, freshwater eutrophication, and land use.

**Conclusions:**

Adopting an electricity mix featuring substantial proportions of nuclear and wind energies, coupled with the use of biomass alongside municipal solid waste for kiln fuel, and integrating battery electric trucks, emerges as a promising alternative. However, this optimal scenario for CO_2_-eq reduction might not align with the best outcomes across all impact categories. Specific attention is warranted, particularly regarding nuclear sources for electricity and increasing land use due to expanding renewable energy sources.

**Supplementary Information:**

The online version contains supplementary material available at 10.1007/s11367-025-02537-5.

## Introduction

The construction sector exerts substantial impacts across the three pillars of sustainability: the environment, the economy, and society, making its role in sustainable development critical (United Nations Environment, 2021). The industry’s activities contribute significantly to global challenges like carbon emissions and resource depletion, while its outputs—the buildings and infrastructure we inhabit—fundamentally shape social well-being and economic progress. Therefore, a thorough consideration of these multifaceted impacts is essential for advancing the sustainable development agenda.

Among the many materials used in construction, concrete stands out due to its central role and significant environmental footprint (Gregory et al. 2021). As a key component of modern infrastructure, its production process is highly resource- and energy-intensive, leading to substantial greenhouse gas (GHG) emissions (Habert et al. 2020). The combination of high demand, widespread use, and carbon-intensive manufacturing makes concrete a crucial focus for sustainability efforts within the construction sector. Addressing its environmental impact is therefore essential in the transition toward a low-carbon and resource-efficient built environment.

Notably, global initiatives such as net zero and circular economy strategies have gained prominence in guiding the construction industry to significantly improve its environmental impacts. Governments and organisations worldwide are increasingly adopting comprehensive net zero and circularity commitments, setting ambitious targets to achieve a balance between emitted and removed carbon dioxide. Some examples of this include industry-wide strategies, such as UK Green Building Council (2021), but several more specific documents exist, like for cement (Mineral Products Association 2015), steel (British Constructional Steelwork Association 2021), or asphalt (Asphalt Industry Alliance 2021).

Until the present, researchers have explored various approaches to mitigate the environmental impact of concrete. A widely evaluated approach has been the use of alternative materials and waste products in concrete production. Examples of this are the incorporation of fly ash and silica fume in concrete mixes (Onyelowe et al. 2022), or the use of recycled and by-product aggregates in concrete (Roh et al. 2020). Another studied approach is the development of alternative binders for concrete. For instance, alkali-activated materials have been shown to be promising sustainable alternatives to Portland cement (White 2020).

While material-focused approaches are important and can provide several significant benefits, their success in terms of reducing emissions in the construction sector has been proven to be heavily dependent on the transition to cleaner energy systems (Rogelj et al. 2015). In the construction sector, where concrete plays a significant role, the energy sources used for production can have a profound effect on the overall carbon footprint (Habert et al. 2020). Low carbon energy systems not only align with the imperative to reduce emissions, but can also contribute to the broader circularity agenda by minimising resource consumption and waste generation (Hailemariam and Erdiaw‐Kwasie 2022; Stewart and Niero 2018). In fact, several studies highlight the importance of integrating prospective energy models into LCA to assess future scenarios accurately. For instance, Hache et al. (2020) analysed the interplay between energy transition policies and cement demand, demonstrating the complex dependencies between material use and evolving power generation technologies; García-Gusano et al. (2017) applied prospective LCA to electricity systems, showing how different long-term electricity scenarios can affect environmental impacts; and Louis et al. (2018) discussed the integration of LCA into long-term energy planning models, reinforcing the need for prospective life cycle approaches in sustainability assessments.

While a growing body of prospective research provides a critical foundation for assessing decarbonisation pathways for cement and concrete, important methodological and scope limitations remain. Georgiades et al. (2023) and (Müller et al. 2024), for instance, present prospective LCAs of cement production in Europe, with a primary focus on upstream plant-level interventions such as fuel switching, kiln improvements, and CCS. However, their system boundaries exclude downstream concrete and building-scale implications and are mostly limited to GHG-related metrics. Alaux et al. (2022) and Alig et al. (2021) incorporate energy projections in prospective LCAs of alternative materials but are either restricted to single buildings or omit a comprehensive treatment of environmental trade-offs. Broader strategic studies, such as Barbhuiya et al. (2024) and Rihner et al. (2025), highlight decarbonisation levers and sectoral challenges but lack detailed, scenario-specific LCA modelling and often exclude key contributors such as transport emissions.

In light of the above and given the substantial impacts of the construction sector and the unique challenges posed by concrete, it becomes imperative to assess how various net-zero strategies will influence the environmental impacts of concrete production chains. These strategies aim to drive a transition towards more sustainable practices, reduce carbon emissions, and enhance the efficient use of resources. Understanding their potential effects on the concrete supply chain is essential for guiding policy, investment, and operational decisions within the construction industry.

Thus, the objective of this article is to analyse the environmental impacts of decarbonisation strategies within the construction sector, using an LCA approach at multiple levels—from cement and concrete production to building construction. The study explores various scenarios that assess the impact of low carbon electricity sources (e.g., renewable grid integration), fuels (i.e., replacing fossil fuels with alternative energy sources), and transportation methods (i.e., electrification). Furthermore, the study employs a multi-objective discrete optimisation (MODO) framework to move beyond simple impact assessment, identifying optimal pathways that balance CO_2_-eq reductions against overall environmental performance. The results of this study can inform policymakers, industry leaders, and researchers to guide decision-making processes based on effective strategies to transition toward a more environmentally responsible construction sector.

## Methods

This section describes the LCA method followed, which is based on the ISO 14040/14044 (International Organization for Standardization [ISO] 2006), and the scenarios evaluated, including the definition of goal and scope, life cycle inventory (LCI), life cycle impact assessment (LCIA), and interpretation.

### Goal and scope of the study

The goal of this study is to precisely analyse the environmental impacts of decarbonisation strategies, specifically focusing on energy, transportation, and fuel transitions, within the concrete supply chain in the United Kingdom (UK). This analysis aims to quantify the potential reductions in global warming potential and identify associated trade-offs across other environmental impact categories when implementing these strategies.

Namely, the environmental impacts of different energy, transportation modes, and kiln fuel type scenarios of the concrete supply chain are evaluated. This is done at different levels of the supply chain (i.e., cement, concrete, building) and, therefore, three different functional units (FUs) were defined for this study: the production of 1 ton of cement, the production of 1 m^3^ of concrete, and the construction of 1 m^2^ of a reinforced concrete building. These FUs were defined based on common practice in this field, both in research (Knoeri et al. 2013; Panesar et al. 2017) and industry (Chinfon Cement Corporation 2025; Readymix Industries Ltd. 2022).

The temporal scope of this study reflects current (2025) industry practices for the business-as-usual scenario, with alternative scenarios assessing prospective impacts based on established UK energy and transport decarbonisation roadmaps, as detailed in subsequent subsections. The geographical scope of this study is the UK.

**Fig. 1:**
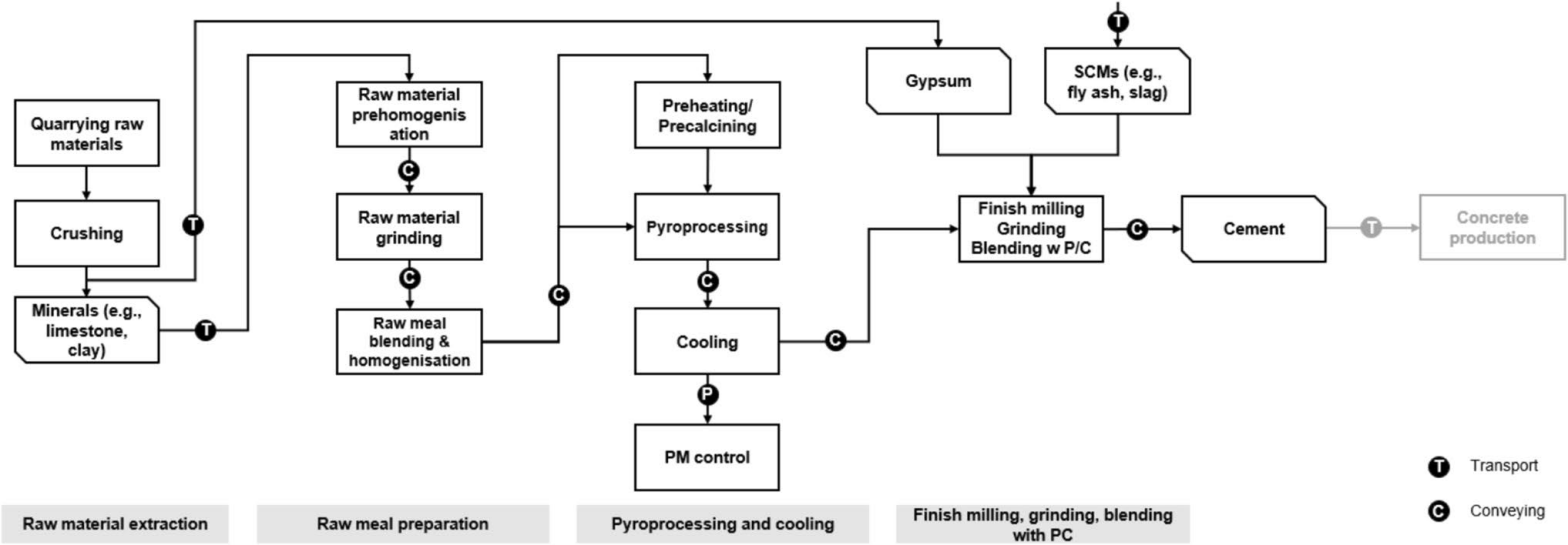
Scope of the cement production LCA

As shown in Fig. 1, the LCA for the cement production includes a set of processes that correspond to modules A1-A3 according to the nomenclature in EN15804 (European Standards 2019). First, the extraction of raw materials (quarrying and raw materials crushing) involves acquiring raw materials from the Earth’s crust and then crushing. In this study, this was considered to be done by an impact crusher. Then, the preparation of the raw meal involves pre-homogenising the material. This can be done through the so-called dry or wet processes. In the former, raw materials are dried, ground, and homogenised separately before being mixed together. In the latter, raw materials are mixed in water to form a slurry before homogenising. Here, it was considered that the dry process was used, and that the blending was performed using a ball mill.

The following step is the pyroprocessing and cooling, which is the process whereby the input materials (e.g., limestone, clay, sand) are subjected to high temperatures in a kiln. This triggers a reaction known as calcination, where the calcium carbonate is transformed into calcium oxide, which then reacts with the other constituents of the input material to form clinker. There are different technologies that can be used for the pyroprocessing (e.g., wet kiln, dry kiln, preheater kiln, precalciner kiln) (Boateng 2016) and cooling (e.g., rotary cooler, planetary cooler) (Petek Gursel 2014). Here, these processes were considered to be done using a preheater kiln and a rotary cooler, respectively. These technologies were selected due to their widespread industrial adoption and their alignment with best available techniques as recommended by the European Commission (2013). The preheater kiln significantly reduces fuel consumption by utilising preheated raw materials, while the rotary cooler improves heat recovery efficiency.

Lastly, the finish milling, grinding, and blending involves grinding the clinker with additional materials like gypsum to obtain a fine powder. Among the different technologies existing for these processes (e.g., tube mill, roller mill, ball mill), it was considered that the tube mill was used. In this process, any supplementary cementitious materials (SCMs), like fly ash or slag, are added to the mix. Once this process is finalised, the cement is transported to the concrete production plant.

**Fig. 2:**
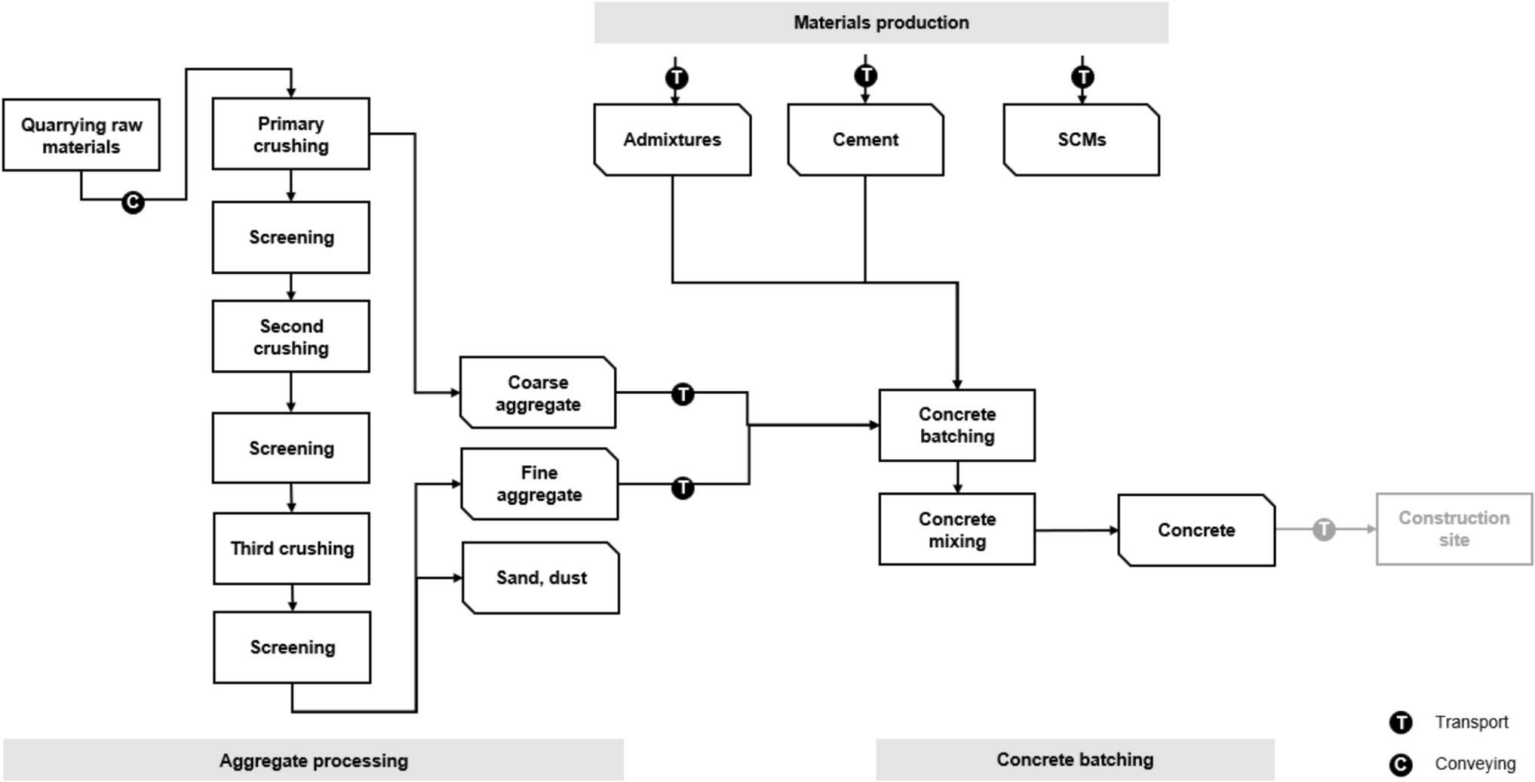
Scope of the concrete production LCA

For the production of concrete, in addition to the stages concerning the cement manufacture, there is also the production of other resources, which includes aggregates, supplementary cementitious materials, and additives, as well as the supply of water. The process for obtaining aggregates was modelled by considering different crushing stages which allow obtaining the different sizes of aggregates, as shown in Fig. 2. The crushing was considered to be done with a cone crusher, and the screening with a horizontal screening technology.

Once aggregates have been obtained and transported to the concrete production plant, they are mixed in the right amounts with cement and other components (i.e., admixtures, SCMs). After batching and mixing, the concrete mix is ready to be transported to the construction site.

**Fig. 3:**
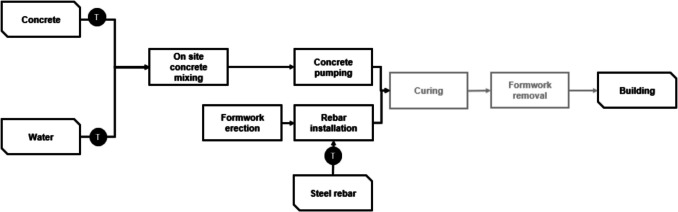
Scope of the building construction LCA

This concrete was considered to be used for the construction of a reinforced concrete building (see Fig. 3). In this study, the structural components of a representative building case study were assessed, specifically focusing on the concrete structure, including foundations, columns, beams, and slabs. Non-structural elements such as finishes, interior partitions, mechanical systems, and fixtures are excluded from the analysis, as the objective is to evaluate the environmental impact of concrete-related materials and construction processes.

For the construction of the building, the concrete mix is combined with water and pumped into the formwork system, where rebar has already been installed. It needs to be noted that the use and end-of-use phases were not considered for the analysis and, therefore, the study considers solely the impacts of the building materials production, their transportation to site, and the construction activities necessary to build the structure. This approach was chosen as the study primarily focuses on decarbonisation strategies in energy, fuel, and transport within concrete production, which are most relevant to the production phase. The use phase was excluded due to its strong dependence on building operation, climate conditions, and user behaviour, introducing significant variability. Likewise, the end-of-life phase involves uncertainties related to regional demolition practices, recycling processes, and landfill policies. While this study does not address these aspects, readers can refer to other studies that explore circularity strategies specifically at the end of life (Backes et al. 2022; Nwonu et al. 2025; Quéheille et al. 2022).

All the resources utilised throughout the process need to be transported to the concrete production plant. Reference distances were considered according to average data for the UK concrete supply chain (MPA The Concrete Centre 2022), and they are included in the Supplementary material.

This study considers five different scenarios: business-as-usual (BAU), low-carbon electricity (LCE), alternative low-emission transport (ALET), alternative fuel substitution (AFS), and a combination of all these scenarios. These strategies were selected to address critical, yet less frequently quantified, aspects of the concrete production chain, guided by three key principles: a focus on production-oriented decarbonisation levers; technological maturity enabling widespread UK deployment by 2035–2050; and alignment with established UK national energy and transport roadmaps. Other potential strategies, such as those focusing on alternative materials and designs, were excluded due to extensive coverage in prior literature and the scope of this study. A summary of all excluded pathways is summarised in the Supplementary material (Table S1).

Details of each scenario assessed are described next.

#### Scenario 1—BAU

The first scenario is the baseline scenario, namely business-as-usual (BAU). As for the electricity, the electricity mix considered is the latest available UK electricity mix, which corresponds to 2022 and is available from UK Department for Energy Security and Net Zero public data (UK Department for Energy Security and Net Zero 2023). It should be noted that not all materials used in the concrete supply chain are extracted and produced within the UK. However, the majority of limestone, gypsum, sand, and gravel, which are key constituents of concrete, are sourced domestically. According to the ONS 2024 Material Flows Account for the UK (Office for National Statistics 2024), domestic extraction of non-metallic minerals accounted for 200.8 million tonnes, whereas imports were limited to 30.2 million tonnes, with key materials like limestone and sand having import rates below 2%. Thus, given the low share of imported materials, the environmental impacts from international electricity mixes are expected to be negligible. Therefore, the UK electricity mix was applied to material production, ensuring a representative impact assessment. Additionally, regarding transportation, it was considered to be made by 16 to 32 ton lorries. The transport distances used in this study were obtained from industry sources (MPA The Concrete Centre 2023), which provide representative data for material transport in the UK construction sector. While it is true that some raw materials may be imported and transported over long distances, a substantial proportion of aggregates, cement, and other materials used in UK concrete production are sourced locally, leading to lower transport distances in most cases. Lastly, a common fuel mix for the kiln was used for the pyroprocessing thermal energy (Petek Gursel 2014).

#### Scenario 2—low-carbon electricity (LCE)

In this scenario, the BAU assumptions considered (see previous section) were used, except those regarding the electricity mix. Here, different possible electricity mixes were defined based on roadmaps to the decarbonisation of the UK electricity network. In particular, the roadmaps presented below were used, from which five alternatives of electricity mixes were extracted. Table 1 presents a summary of the defined scenarios. Note that this study does not include carbon capture and storage (CCS) due to its low technology readiness level (TRL) for cement applications and the lack of large-scale deployment data. While CCS is recognised as a key decarbonisation pathway in cement industry roadmaps (Cembureau n.d; Global Cement and Concrete Association 2021), full-scale implementation remains in early pilot stages, with commercial feasibility expected only after 2030–2040. Unlike the strategies assessed in this study—such as fuel substitution, grid decarbonisation, and transport electrification—CCS is still under development and lacks operational life cycle data for robust environmental modelling. Future research should integrate CCS as deployment progresses and more industry data become available.

**Table 1:** Electricity mix scenarios (unit: %)

	BAU	LCE1^1^	LCE2^2^	LCE3^3^	LCE4^2^	LCE5^2^
Coal	1.71	0.00	0.00	0.00	0.00	0.00
Oil	0.68	0.00	0.00	0.00	0.00	0.00
Gas	38.42	0.67	0.47	1.17	4.70	0.88
Nuclear	14.67	51.67	22.99	2.11	4.44	4.19
Hydro (natural flow)	1.73	0.00	0.00	0.00	0.00	0.00
Onshore wind	10.83	8.91	7.11	13.15	10.70	12.80
Offshore wind	13.84	29.18	45.73	26.06	31.85	30.46
Shoreline wave/tidal	0.00	3.34	10.19	0.00	0.00	0.00
Solar	4.08	2.90	10.19	27.00	18.80	21.41
Bioenergy	11.01	2.90	2.61	0.94	3.39	3.31
Pumped storage	0.61	0.00	0.00	22.30	15.14	18.76
Other fuels	2.39	0.45	0.71	7.28	10.97	8.17

^1^Based on Scenario ESC-C (Energy Systems Catapult 2020)^2^Based on Scenario ESC-P (Energy Systems Catapult 2020)^3^Based on Scenario FES-LTW (National Grid ESO, 2020)^4^Based on Scenario FES-ST (National Grid ESO, 2020)^5^Based on Scenario FES-CT (National Grid ESO, 2020)

The first two mixes were adapted from the 2020 *Innovating to Net Zero* report by Energy Systems Catapult (ESC) (Energy Systems Catapult 2020). This report presents two different pathways for decarbonisation of the energy system, namely the “Clockwork” (LCE1) and the “Patchwork” (LCE2). The former represents a scenario where there is no major behaviour change and large-scale centralised changes to energy supply, which are accompanied by important technology advances; the latter describes a scenario where there are major behavioural changes and less progress in terms of technology advance.

The remaining three mixes were adapted from the 2020 *Future Energy Scenarios* (FES) report by National Grid ESO (2020). In this report, the pathways defined are the “Leading the Way ” (LCE3), “System Transformation ” (LCE4), and “Consumer Transformation ” (LCE5). The first scenario considers that there is a large societal change. The second scenario considers that there is no major behavioural change, and the changes are large-scale centralised. In the third scenario, there is a near-total electrification of demand.

According to (Mineral Products Association 2023; Office for National Statistics 2023), while there is a part of the raw material used in the concrete supply chain that is imported, this represents a low percentage overall. Therefore, in this study, it was assumed that all material is extracted nationally.

#### Scenario 3—alternative low-emission transport (ALET)

Some sources (Moultak et al. 2017; Speth & Funke 2021) have indicated that battery electric trucks (BETs) are a promising solution for emissions reduction in road freight transport. Therefore, in this alternative scenario, road freight transport is considered to be done using BETs.

Two distinct conditions are examined within this cleaner transportation scenario: one involving normal trucks covering the baseline transportation distance, and the other utilising BETs for the same baseline distance. Additionally, to assess the robustness of the cleaner transportation approach, both normal trucks and BETs were evaluated for an extended transportation distance (+ 200%). This analysis tests whether the environmental benefits of BETs persist under more demanding conditions, ensuring that the advantages observed at shorter distances are not negated when transportation distances increase. By doing so, we evaluate the reliability of this decarbonisation strategy across different logistical conditions.

#### Scenario 4—alternative fuel substitution (AFS)

In recognition of the important role that fuels play in the overall environmental impacts of concrete production, the cleaner fuels scenario explores the potential benefits of alternative fuel mixes. In particular, this scenario considers the fuel mix scenarios shown in Table 2. Note that, in reality, the specific percentages can vary based on factors such as the availability of waste feedstock, technological capabilities, and regulatory constraints.

**Table 2:** Kiln fuel combinations for use in the kiln (unit: %)

Fuel	BAU^1^	Solvent^2^	Biomass^3^	MSW 1^4^	MSW 2^4^	Paper and plastic^5^
*Conventional fuels*						
Bituminous coal	65	15	15	30	5	0
Lignite coal	0	15	15	0	0	0
Distillate (diesel or light) fuel oil	0.8	10	0	0	0	0
Petroleum coke	22	0	0	0	0	0
Residual fuel (heavy) oil	0.2	0	0	0	0	0
Natural gas	4	0	0	0	0	0
Biomass	0	0	70	35	50	0
*Waste*						
Waste oil	1	0	0	0	0	0
Waste solvent	4	60	0	0	0	0
Waste tire	3	0	0	0	0	0
Waste paper	0	0	0	25	30	50
Waste plastics	0	0	0	10	15	50
Waste sewage sludge	0	0	0	0	0	0

^1^Based on Petek Gursel (2014)^2^Partially based on Seyler et al. (2004)^3^Partially based on MPA et al. (2019)^4^Partially based on Burnley (2007); hypothetical case assuming solely the use of recycled paper and plastic

The first scenario (BAU) is based on current practice, where a major proportion of the fuel is based on coal and coke. Then, the following scenario (named here Solvent) is based on the consideration of waste solvent as an alternative to fossil fuels, option which has been studied in the past (Seyler et al. 2004). The scenario Biomass is based on the assumption that a high percentage of biomass, combined with lower proportions of fossil fuels, is used in the kiln. The use of biomass holds great potential in this area and has been studied in the past (Gallego Fernández et al. 2019). The following two scenarios (MSW 1, MSW 2) are combinations of conventional and waste fuels in different proportions. Lastly, the last scenario focuses on a combination of waste paper and plastic. While the disposal of plastic from various packaging is becoming more complex, the potential for utilising waste plastic for fuel production has been explored (Patni et al. 2013).

#### Scenario 5—combined scenarios

Scenario 5 represents an aggregation of all individual strategies assessed in Scenarios LCE, ALET, and AFS. By combining these measures, this scenario provides insight into the potential cumulative benefits of implementing multiple strategies simultaneously. The combined impact is assessed across all environmental indicators to evaluate whether synergies or trade-offs emerge when applying all decarbonisation levers together.

### Life cycle inventory analysis

This stage of the LCA involves gathering information for each unit process pertaining to the relevant energy and mass flow inputs and outputs, as well as data on emissions into the atmosphere, water, and soil.

Based on the system described before, data from the industry and the literature were collected, where industry data correspond to publicly available sector data from the cement and concrete industries as well as commercial data. A summary of the data used is shown in Table 3 and the reader is referred to the Supplementary material for further details. The processes that are the subject of this study are situated in the UK. Therefore, whenever possible, the LCI data refer to local suppliers and manufacturers.

**Table 3:** Summary of LCI data used and respective sources

Item	Data sources*			
	Lit	Com	Ecoinv	
Electricity	●		●	BAU: UK Department for Energy Security and Net Zero (2023), cleaner electricity scenarios: Energy Systems Catapult (2020); (National Grid ESO, 2020) Data adapted from Ecoinvent: medium voltage electricity – electricity voltage transformation from high to medium voltage
Transport	●		●	MPA The Concrete Centre (2023) Data adapted from Ecoinvent. For the BAU: freight transport, lorry 16–32 metric ton, EURO5. For the alternative scenario, adapted from electric vehicle
Conveying	●	●		Commercial data (Claudius Peters, Agico Cement, SDDOM), Petek Gursel (2014)
Cement production				
Raw materials	●		●	Marceau et al. (2007) Data adapted from Ecoinvent: tap water production, conventional treatment; limestone, unprocessed; clay; shale; sand; anhydrite rock; iron ore; gypsum, mineral
Pyroprocessing	●		●	Bhatty et al. (2004); Boesch and Hellweg (2010); Boesch et al. (2009) Data adapted from Ecoinvent: diesel, burned in building machine; bituminous coal; distillate fuel oil; petroleum coke; residual fuel oil; natural gas; waste mineral oil; solvent mixture; pneumatic tyres; waste paper; waste plastic; sewage
Crushing		●		Commercial data (Sinonine, Chaeng)
Milling and grinding	●			Marceau et al. (2007)
Concrete production				
Aggregates			●	Data adapted from Ecoinvent: gravel
Mixing and batching	●	●		Commercial data (Meka Global), Cembureau (1999); Marceau et al. (2007)
Building				
Building			●	Data adapted from Ecoinvent: gravel; reinforcing steel

*All data adapted from Ecoinvent is cut-off by classification – unit. Lit: literature, Com: commercial data, Ecoinv: Ecoinvent

The software used for the modelling was SimaPro v9.6. While foreground systems were modelled using industry and literature data, the Ecoinvent v3.11 database was used to represent background systems (other process inventories, e.g., fuel and raw material production data). The most recent available data were utilised, which allows ensuring that the processes examined in this study are based on contemporary technologies and standard production conditions.

The LCI was built in the form of a model comprised of several integrated worksheets. This model allows the user to select the type of cement (e.g., CEM I, CEM II), the raw materials for the cement, different technology options for the different processes, the fuel mix used for pyroprocessing, the kiln type, the strength class of concrete, the concrete mix, geographical considerations for the grid mix, conveying technology options and distances, transportation distances and modes, and information regarding the building (e.g., material quantities).

Considering the extensive array of choices available, including composition, material type, and other factors, the subsequent bullet points delineate the specific selections made for the various inputs within the model. Detailed inventory data can be referenced in the Supplementary Material.

- **Cement**. In this study, the cement type investigated was CEM I. A complete mixture description for the cement is provided in the Supplementary Material.
- **Concrete**. A concrete C25 was considered in this study, with the composition mix as shown in the Supplementary Material.
- **Building**. A model of a 5-story reinforced concrete building was analysed in this study. Details of the design can be found in the Supplementary Material.

### Impact assessment

The LCIA phase assesses the environmental impacts and gauges the resources consumed within the modelled system. This phase comprises two essential components: the selection of impact categories, which involves categorising LCI outcomes (emissions, waste, and resources) into the chosen impact categories, and the characterisation process, where the transformed LCI data is combined to produce an indicator result. This indicator result represents the ultimate outcome of the essential aspect of LCIA. Additional steps like normalisation, grouping, weighting, and a supplementary analysis of LCIA data quality are discretionary and were not undertaken in this study.

For the impact assessment, characterisation factors were collected for inputs and outputs of the system for each scenario. These factors were sourced from Ecoinvent v3.11 and the impact assessment method used was ReCiPe midpoint (hierarchical).

### Interpretation

In the interpretation stage of LCA, the results of the analysis are evaluated, and conclusions are drawn. This stage involves identifying the key findings of the LCA, understanding their implications, and communicating them to the intended audience.

Sensitivity analysis allows testing how variations in specific input parameters affect the results of the LCA. This is important for identifying which factors have the greatest impact on the environmental performance of the concrete supply chain and assessing the robustness of conclusions. However, it is important to distinguish sensitivity analysis from uncertainty analysis. Sensitivity analysis evaluates the effect of specific parameter changes, whereas uncertainty analysis focuses on quantifying the overall reliability of the model, including data quality, methodological assumptions, and background database uncertainties.

In this study, sensitivity analysis was embedded in each of the defined scenarios. Specifically, in Scenario LCE, different grid mixes were considered; in Scenario ALET, alternative transportation modes and distances were analysed; and in Scenario AFS, varying proportions of fuel mixes were assessed. Moreover, all these variations were integrated into the combined scenario to evaluate cumulative effects. While this approach provides insights into the influence of key parameters on LCA results, a formal uncertainty analysis was not performed.

In fact, it needs to be noted that while some scenarios exhibit significant differences in environmental impacts, it is important to acknowledge that even large variations may still fall within the uncertainty range of the LCA model. Factors such as data variability, assumptions in background databases, and methodological choices contribute to this uncertainty. Therefore, these differences should be interpreted with caution, and further uncertainty analysis (e.g., Monte Carlo simulations) could provide a more robust assessment of result variability.

### Multi-objective optimisation

To assess the optimal trade-offs between CO_2_-eq emission reductions and overall changes in other environmental impact categories, a multi-objective discrete optimisation (MODO) approach (Liu et al. 2020) was implemented. MODO is considered an adequate approach for addressing sustainability problems, where there are often conflicting criteria (Limleamthong & Guillén-Gosálbez 2018; Yuan et al. 2023). In this study, the goal was to minimise CO_2_-eq emissions while maximising reduction of impacts in all the other impact categories, measured as the percentage change across multiple environmental impact categories.

The optimisation problem was thus structured as follows:

Objective 1:

1 $$\underset{{S}_{i}}{\mathrm{min}}\left(C{O}_{2}-eq\left({S}_{i}\right)\right)$$

Objective 2:

2 $$\underset{S_i}{\mathrm{max}}\left(-Total\;impact\;change\left(S_i\right)\right)$$

where CO_2_-eq(S_i_) represents the total CO_2_-eq emissions for scenario S_i_, and Total Impact Change (S_i_) denotes the sum of the percentage changes in environmental impact categories (without including GWP), with negative values representing reductions in impact.

Since the dataset consists of predefined discrete scenarios, a Pareto front optimisation approach was employed to identify the set of non-dominated solutions, where no scenario was strictly better in both objectives.

A scenario S_i_ was considered Pareto-optimal if no other scenario S_j_ (j ≠ i) satisfied:

3 $$CO_2-eq\left(S_j\right)\leq CO_2-eq\left(S_i\right)and\;Total\;impact\;change\left(S_j\right)\leq Total\;impact\;change\left(S_i\right)$$

with at least one inequality being strict.

The set of optimal solutions was determined by iterating through all scenarios and filtering out dominated solutions. If no optimal solutions were found, a trade-off approach was implemented to balance CO_2_-eq minimisation with acceptable total impact reductions. In particular, a threshold (ϵ) was defined to select scenarios with total impact reductions within the top 10% of the dataset. Among these scenarios, the one with the lowest CO_2_-eq emissions was selected as the optimal trade-off solution.

This approach ensures that the selected scenario not only minimises CO_2_-eq emissions but also maintains a strong environmental performance across all other impact categories, thus avoiding solutions that might significantly reduce CO_2_-eq emissions at the cost of increasing other environmental burdens. It is important to acknowledge that this optimisation approach assigns equal weights to all environmental impact categories, assuming that all categories contribute equally to sustainability objectives. While this provides a balanced approach, it may not fully reflect the relative importance of different environmental impacts in specific contexts.

## Results

This section presents the results for the scenarios analysed. Given that the main objective was to evaluate the change in impact resulting from different strategies, the visuals utilised here are based on relative values (i.e., percentage changes). However, all results in absolute values can be found in the Supplementary material.

### Scenario low-carbon electricity (LCE)

**Fig. 4:**
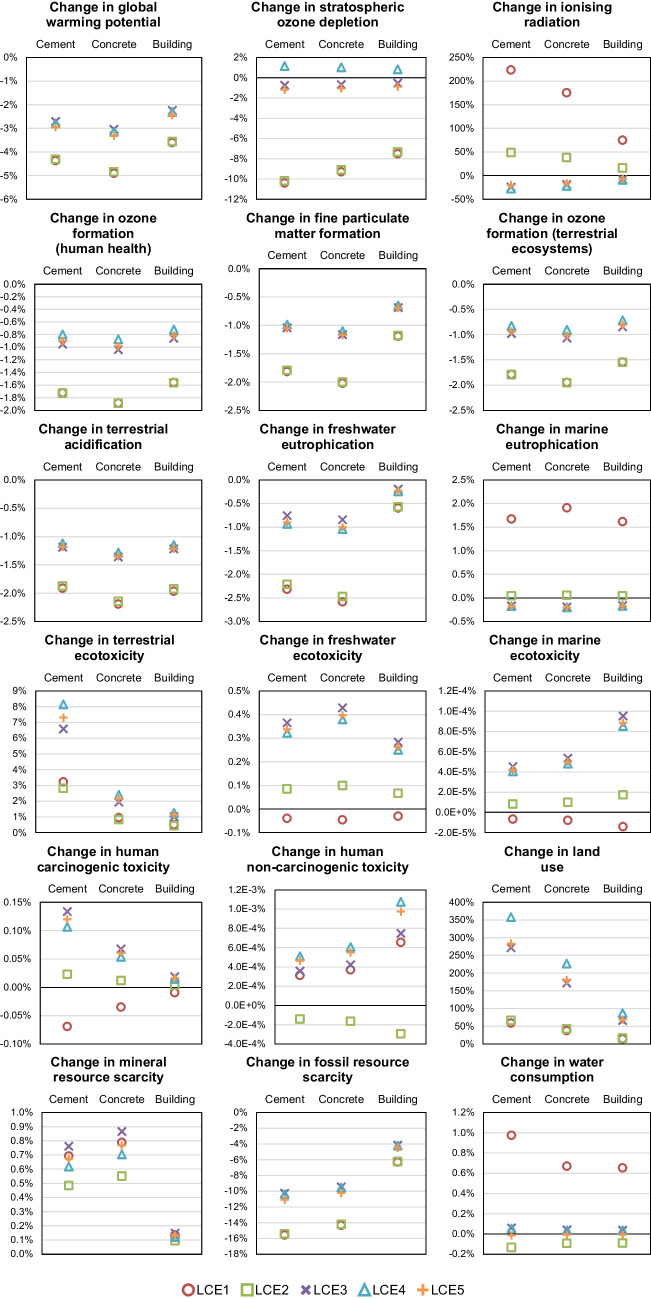
Results of different electricity mix scenarios on the impacts of cement (FU: 1 ton), concrete (FU: 1 m^3^), and building (FU: 1 m^2^)

The results of different impact categories considering different electricity mixes are shown in Fig. 4. The figure presents a series of bar graphs comparing the percentage changes in various environmental impacts associated with cement, concrete, and buildings made from concrete. It consists of multiple graphs, each of which focuses on a different impact category. In each graph, the results for the three FUs studied are compared (i.e., 1 ton of cement, 1 m^3^ of concrete, 1 m^2^ of a building). In the graphs, the y-axis represents the percentage change in comparison to the BAU scenario, with the 0% indicating no change with respect to that scenario. A positive change indicates an increase of impacts, whereas a negative change indicates a decrease of impacts.

As it can be seen, when cleaner electricity mixes are applied to cement production, the environmental impact is diverse. Namely, among the various impact categories, there is a blend of positive and negative effects. The changes in environmental impacts for concrete and the building are generally consistent with the trends observed for cement, albeit with slight percentage changes in some instances.

In seven impact categories (i.e., global warming potential, ozone formation – human health, fine particulate matter formation, ozone formation – terrestrial ecosystems, terrestrial acidification, freshwater eutrophication, and fossil resource scarcity), cleaner electricity contributes only to reductions in the impacts, potentially mitigating the environmental footprint of the different stages of the concrete supply chain analysed. In most of these categories, the variation in the impact is low (less than 4%), except for global warming potential and fossil resource scarcity.

Regarding global warming potential, in the study by (Tangthieng 2017), the results showed that indirect CO_2_-eq emissions from electricity production for cement production were around 8% of the total emissions from cement, which is consistent with the results obtained in this study, where improving electricity grid sources allows for a reduction of between 2.7 and 4.4% of emissions of cement. As for fossil resource scarcity, the BAU electricity mix relies on fossil fuels for its production, which consequently exacerbates the scarcity of fossil resources in comparison to cleaner electricity scenarios studied herein.

In three of the impact categories analysed (i.e., terrestrial ecotoxicity, land use, mineral resource scarcity), the change in electricity sources leads to a worsening of environmental impact for cement. Previous research has indicated that the adoption of renewable energy sources may paradoxically contribute to elevated environmental impacts. For instance, the study by Mahmud and Farjana (2022) showed that the use of photovoltaic and wind power plants can have high environmental impacts in terms of terrestrial ecotoxicity.

The transition to renewable energies may intensify the global competition for land, as these technologies often require larger areas for deployment compared to conventional energy sources (Capellán-Pérez et al. 2017). Additionally, the development of renewable energy resources can lead to changes in land use, potentially affecting terrestrial ecosystems and wildlife (Moore‐O’Leary et al., 2017).

In the case of mineral resource scarcity, the percentage changes with respect to the BAU scenario are low (increases of impact between 0.1 and 0.9%). This is consistent with previous literature (Lieberei & Gheewala 2016), which has pointed out that the use of certain renewable energy sources may contribute to mineral resource depletion.

As it can be seen, there are eight impact categories for which some scenarios lead to a decrease and some to an increase in estimates of impacts (i.e., stratospheric ozone depletion, ionising radiation, marine eutrophication, freshwater ecotoxicity, marine ecotoxicity, human carcinogenic toxicity, human non-carcinogenic toxicity, and water consumption).

The change observed for ionising radiation is between −22 and 224%. Such a substantial increase is given by scenario LCE1, which corresponds to a grid mix with a high percentage (51.7%) of nuclear energy. This result is supported by previous literature (Zhang & Bauer 2018), from which it emerged that nuclear electricity generation is linked to high ionising radiation, mostly due to the processes of uranium mining and milling and operation of the nuclear power plant.

For marine eutrophication, the percentage change of the impact for the different scenarios is between −0.2 and 1.7%, where the positive values correspond to scenarios LCE1 and LCE2. These two scenarios are the only ones that have a proportion of tidal energy in the mix, which has been associated in the literature with the potential of increasing marine eutrophication (Kadiri et al. 2014).

Photovoltaic panels have been shown to increase impacts in freshwater ecotoxicity (Kabayo et al. 2019), which explains the reason why scenarios LCE3, LCE4, and LCE5, which have higher proportions of solar energy, yielded slight increases in these impacts.

Regarding water consumption (change between −0.1 and 1.0%), previous studies have shown that certain renewable energy sources might increase this impact category due to the need for water in processes like cooling for solar power plants.

In three of the cases (i.e., marine ecotoxicity, human carcinogenic toxicity, human non-carcinogenic toxicity), the variations observed with respect to the BAU scenario are lower than 0.2%. Such variation can be considered negligible given potential uncertainties in the data.

### Scenario alternative low-emission transport (ALET)

**Fig. 5:**
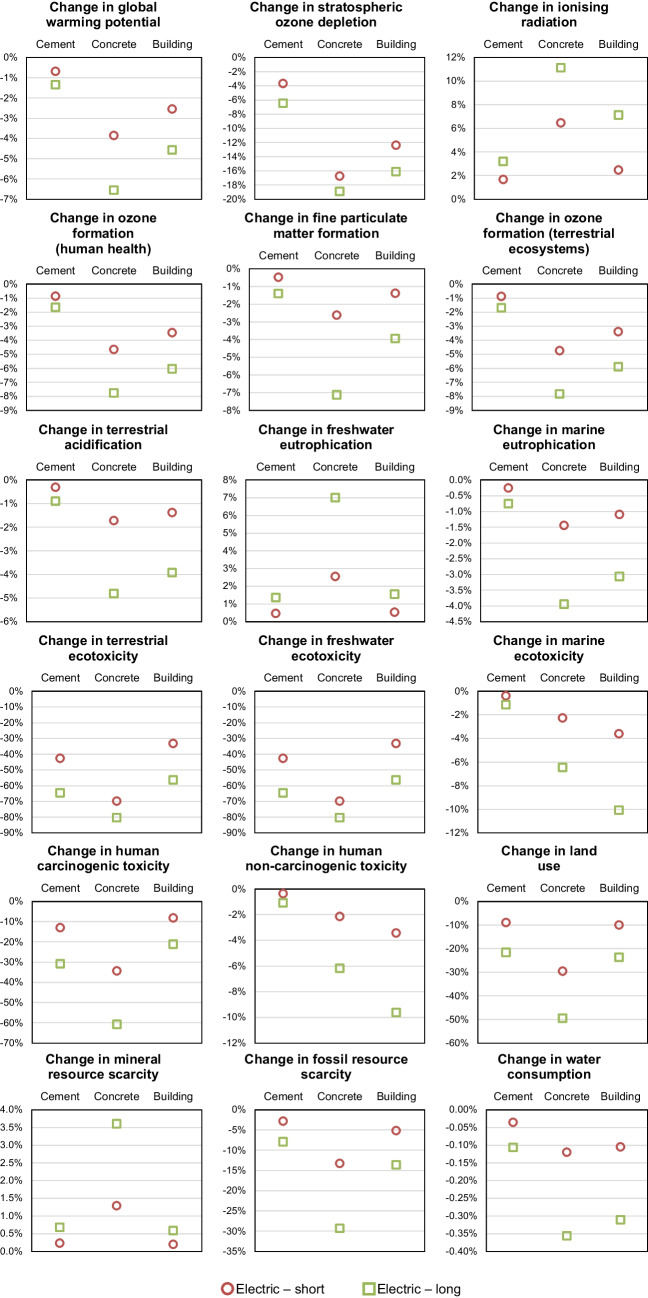
Results of different transportation scenarios on the impacts of cement (FU: 1 ton), concrete (FU: 1 m^3^), and building (FU: 1 m^2^)

The outcomes for all impact categories, considering various transportation methods and distances, are depicted in Fig. 5. This figure illustrates the percentage change concerning the BAU scenario across different environmental parameters, considering the production of 1 ton of cement, 1 m^3^ of concrete, and 1 m^2^ of building. Overall, the percentage change in the impacts of cement tends to be lower than that of concrete and buildings. This discrepancy is not necessarily due to longer transport distances but rather due to the higher volume of materials required for concrete and building construction. Since transportation impacts are calculated in ton-kilometres (t-km), the greater quantity of transported material results in higher total transportation emissions, even if distances remain unchanged. In essence, the higher number of resources transported in concrete and building production amplifies the cumulative impact of transportation, resulting in a relatively higher percentage change in environmental parameters compared to the production of cement alone.

An increase in three impacts due to the use of electric battery vehicles is observed (i.e., ionising radiation, freshwater eutrophication, freshwater ecotoxicity, and mineral resource scarcity). Among these impact categories, the increase in impact is relatively low in mineral resource scarcity (between 0.2 and 3.6% increase).

However, a more significant increase in impacts due to the use of electric battery vehicles is observed in ionising radiation, freshwater eutrophication, and freshwater ecotoxicity. Regarding ionising radiation, according to Cusenza et al. (2019), battery operation phase only impacts significantly the ionising radiation category, which explains the increase in this impact category when it is operating. Regarding freshwater eutrophication, Hawkins et al. (2012) also identified an increase in freshwater eutrophication when comparing conventional vehicles with electric ones. Regarding freshwater ecotoxicity, in the past studies have shown that ionic liquids (Mehrkesh & Karunanithi 2016), and in general electric batteries (Mahmud & Tasmin 2022), can have significant impacts in terms of aquatic toxicity in spite of allowing to limit atmospheric pollution.

For the remaining categories (i.e., global warming potential, stratospheric ozone depletion, ozone formation, fine particulate matter formation, terrestrial acidification, marine eutrophication, terrestrial ecotoxicity, marine ecotoxicity, human toxicity, land use, fossil resource scarcity, and water consumption), a decrease in impacts is observed. In one of these impact categories (i.e., water consumption), the increase is less than 1%.

A more significant increase is observed in the remaining impact categories. The fact that the use of electricity to power trucks results in much lower climate impacts and primary energy use compared to diesel-based pathways has been proved in the past literature (see, for instance, Sathre and Gustavsson (2023). In addition to global warming potential, electric battery trucks reduce fossil resource scarcity due to the significant reduction in fossil-fuel dependence.

### Scenario alternative fuel substitution (AFS)

**Fig. 6:**
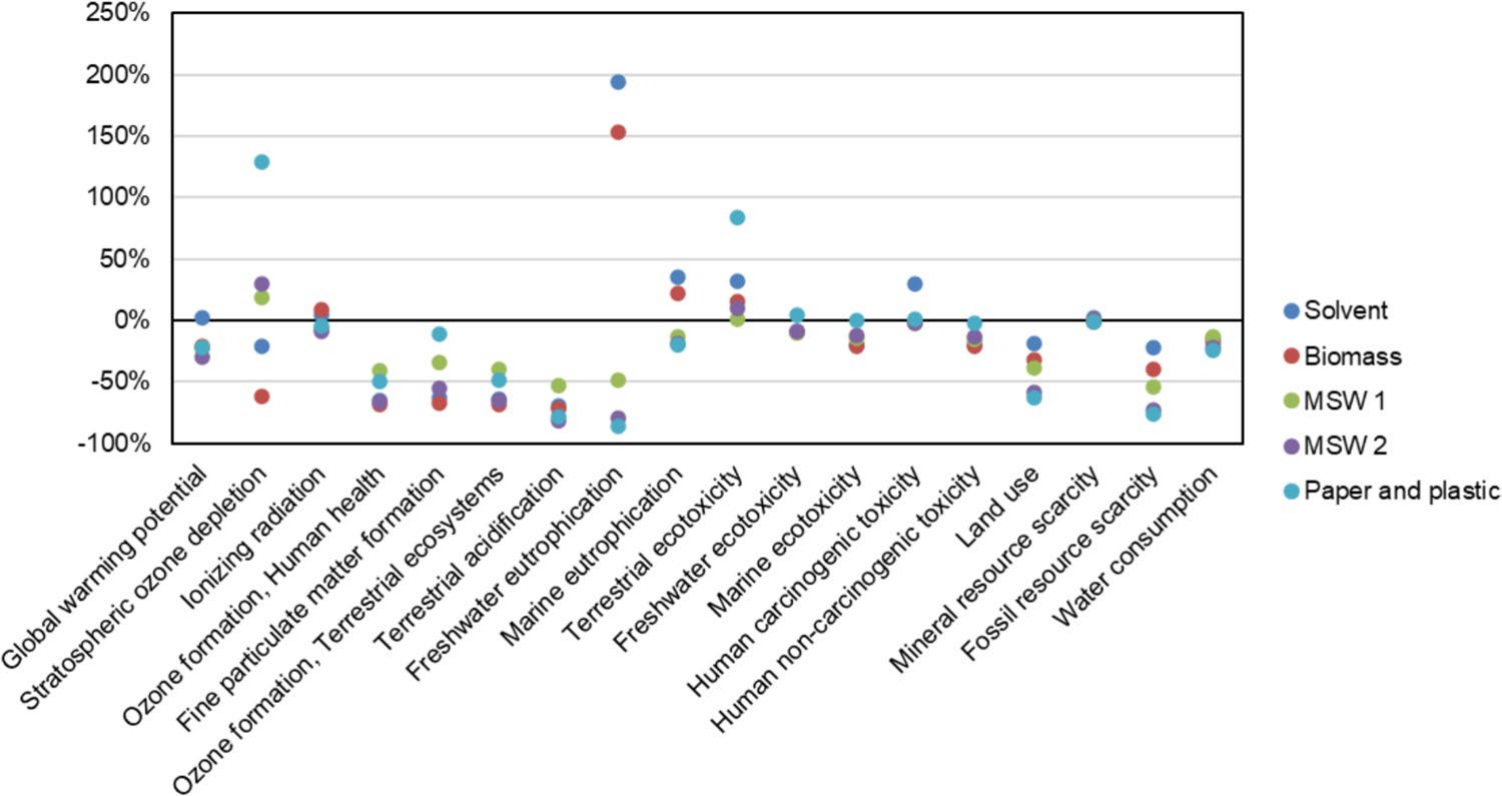
Results of different fuel mix scenarios on the impact of cement (FU: 1 ton)

The results of the impact categories considering different fuel mix are shown in Fig. 6. In this case, given that this scenario has only a direct effect on the cement production, the results shown correspond to percentage change of impacts of cement.

As it can be seen, the environmental implications of using waste as a fuel source are multifaceted. In eight of the impact categories (i.e., ozone formation, fine particulate matter formation, terrestrial acidification, marine ecotoxicity, human non-carcinogenic toxicity, land use, fossil resource scarcity, and water consumption) there exist potential benefits, which are due to the decrease in fossil fuels in the kiln, with the associated reduction in gaseous and particulate emissions, as well as the reduction in the use of fossil fuels.

In the impact category of terrestrial ecotoxicity, all scenarios exhibit an increase in impact, ranging from 1.6% to 83.7%. The most substantial rise occurs in the Paper & plastic scenario, where terrestrial ecotoxicity escalates due to the release of toxic substances into the environment (Alston & Arnold 2011). The incineration of paper and plastic generates emissions of harmful chemicals and compounds, notably including heavy metals, dioxins, and furans. These emissions have been identified as major contributors to terrestrial ecotoxicity, underscoring the environmental implications associated with the incineration of paper and plastic materials.

However, in the remaining impact categories (i.e., global warming potential, stratospheric ozone depletion, ionising radiation, freshwater eutrophication, marine eutrophication, freshwater ecotoxicity, human carcinogenic toxicity, and mineral resource scarcity) there is either an increase or a decrease depending on the scenario analysed.

For global warming potential, all scenarios allow reducing the CO_2_-eq emissions (between 20 and 30%), except for the Solvent scenario, which relies still on coal in spite of using a percentage of waste and leads to a 2% increase. The same is observed for freshwater eutrophication, marine eutrophication, and human carcinogenic toxicity, where this scenario yields an increase in impacts.

As it can be observed, stratospheric ozone depletion shows changes between 128.9% and −61.6% of kg CFC11-eq. The increase in impacts is due to the release of halogenated compounds when paper and plastic are burnt, such as chlorine and bromine, into the atmosphere, compounds which have been identified as the main drivers of stratospheric ozone depletion (Solomon, 1999). A similar phenomenon happens with terrestrial ecotoxicity, where the paper and plastic scenario yields the worst results.

This being said, an important consideration to be made is the effects that using waste as fuel in the kiln has on the properties of the cement. Studies in the past have shown that using waste as fuel in cement kilns could lead to changes in the physico-chemical properties of clinker. As stated by Cortada Mut et al. (2015), using alternative fuels can lead to incomplete combustion and increase the internal circulation of some elements (e.g., sodium, potassium) that affect the process stability and operation by forming buildups and blockages, rings, and shell corrosion. This may result in a decrease in clinker production, a higher consumption of heat, and stops of the cement plant. In fact, according to Ariyaratne et al. (2015), the replacement ratio of coal energy should not exceed 50% to avoid potential negative effects on the product quality and process.

Additionally, substituting fossil fuels by alternative fuels (e.g., waste) requires some further considerations at present, like their availability in large amounts, legislation, and permissions to be able to use a specific alternative fuel, as well as practical issues such as transportation and handling of the fuel (Cortada Mut et al. 2015).

According to the study by Verma et al. (2018), most studies focus on the use of 5 to 20% of waste tires in fuels. Georgiopoulou and Lyberatos (2018) considered the substitution between 10 and 30% of fossil fuels by alternative fuels.

Lastly, it needs to be mentioned that, in addition to fuel-related strategies, improvements in kiln design and technology can also contribute to the improvement of environmental impacts. For example, Mikulčić et al. (2016) identified the use of more energy-efficient kiln processes as a potential mitigation measure.

### All scenarios

There are ten impact categories (i.e., global warming potential, ozone formation – human health, fine particulate matter formation, ozone formation – terrestrial ecosystems, terrestrial acidification, freshwater eutrophication, marine eutrophication, marine ecotoxicity, human carcinogenic toxicity, human non-carcinogenic toxicity, fossil resource scarcity, and water consumption) for which the combination of scenarios yields benefits (i.e., decrease in impacts). These benefits primarily arise from the reduction of raw material consumption through two strategies: the use of alternative fuels in kilns—particularly waste-to-energy solutions that replace fossil fuels—and the electrification of transportation, which reduces the reliance on conventional fuels. While these approaches contribute to resource efficiency, this study does not address material recirculation strategies such as reuse or recycling, which are essential components of a comprehensive circular economy framework.

In the remaining eight impact categories (i.e., stratospheric ozone depletion, ionising radiation, freshwater eutrophication, marine eutrophication, terrestrial ecotoxicity, freshwater ecotoxicity, land use, and mineral resource scarcity), the combined scenarios lead to either increases or decreases in impacts depending on the scenarios combined. The changes for freshwater ecotoxicity and mineral resource scarcity are low (changes between −15% and 15%).

The highest percentual increases in impacts arise from ionising radiation and land use, followed by stratospheric ozone depletion. Regarding ionising radiation, it can be observed that alternative scenarios yield changes in impact between −34.6 and 242.5%. The maximum increases are given by scenarios that consider longer distances with electric trucks and LCE1 for the electricity grid mix. As for land use, it was seen in previous sections that scenarios with high proportions of renewable energy in the electricity mix caused an increase in this impact category. In the case of stratospheric ozone depletion and terrestrial ecotoxicity, the highest increase is given by scenarios using electric battery trucks, LCE4, and paper and plastic.

### Optimal trade-off scenarios

**Fig. 7:**
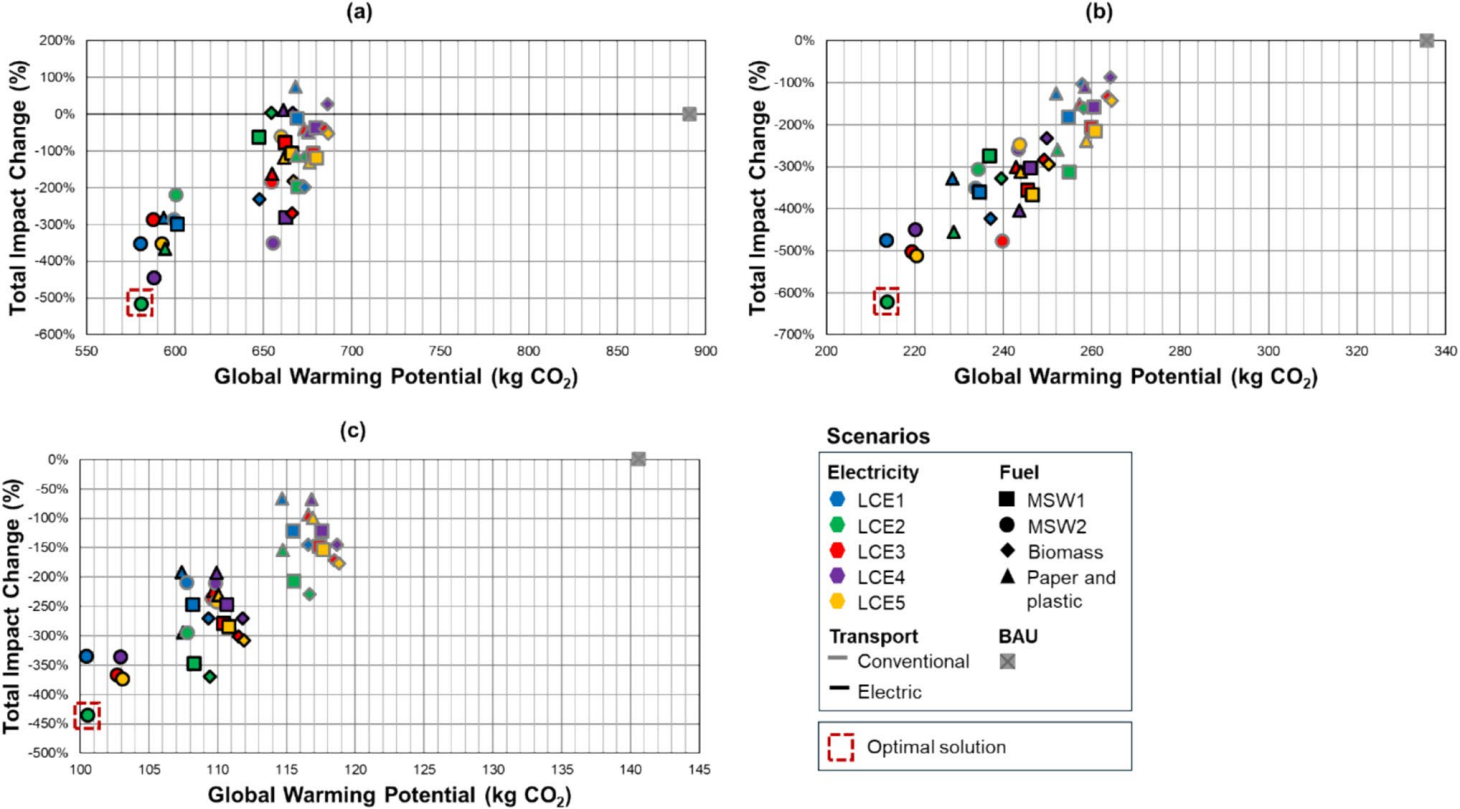
Results of the multi-objective discrete optimisation (MODO) for **a** cement (FU: 1 ton), **b** concrete (FU: 1 m^3^), **c** building (FU: 1 m^2^). GWP on the x-axis and total impact change (%) on the y-axis. Each point represents a unique scenario defined by electricity mix (colour), fuel type (marker shape), and transport mode (border). Optimal scenarios are circled in red. Baseline (BAU) is shown in grey

The results of the MODO, performed to simultaneously minimise CO_2_-eq emissions and maximise the overall impact reduction across the other environmental impact categories, are represented in Fig. 7.

Across all three functional units, the scenarios generally group into two distinct clusters. Scenarios incorporating electric transport consistently populate the lower-left quadrant of each plot, indicating superior performance with both lower GWP and greater overall environmental impact reduction. Conversely, scenarios using conventional transport typically occupy the top-right, representing higher environmental burdens. Notably, a few outliers—specifically those combining the MSW2 fuel mix with cleaner electricity grids (LCE1 or LCE2)—bridge this gap, demonstrating strong performance even with conventional transport.

The optimisation process identified a consistent combination of strategies that offered the most balanced environmental performance. However, the specifics varied slightly for each functional unit.

For cement production, the scenario with the absolute lowest GWP (580 kg CO_2_/t) involved the LCE1 electricity mix and MSW2 fuel. However, this was only the fourth-best option for total impact reduction. The optimal trade-off solution was identified as the combination of LCE2, MSW2, and electric transport, which achieved a superior total impact reduction (−515.5%) at a minimally higher GWP (581 kg CO_2_/t). This optimal choice still resulted, however, in an increase in two impact categories: Stratospheric Ozone Depletion and Ionising Radiation—For concrete production, the scenario with the lowest GWP (213.5 kg CO_2_/m^3^) was not the best overall performer. The optimal solution combined LCE2, MSW2, and electric transport, which yielded the best total impact reduction (−621.7%) and was also the second-lowest GWP option (213.8 kg CO_2_/m^3^), making it the optimal solution. In this case, only the Ionising Radiation impact category increased.

Lastly, for the building construction, the lowest GWP scenario (100 kg CO_2_/m^2^) was the seventh-best total impact reduction, making it non-optimal. The optimal solution was again the LCE2, MSW2, and electric transport combination, which delivered the highest total impact reduction with only a marginal increase in GWP (101 kg CO_2_/m^2^). This combination, however, showed increased impacts for Ionising Radiation and Mineral Resource Scarcity.

The optimisation process highlights that the lowest GWP scenario is not always the best overall environmental option. In both concrete and building cases, the best total impact reduction scenario also closely aligns with the lowest GWP, making it an ideal trade-off solution. However, for cement, a strict GWP minimisation approach would lead to a scenario that does not provide the highest environmental benefits, demonstrating the importance of a multi-criteria assessment. Overall, the selected optimal solutions ensure substantial CO_2_-eq reductions without drastically increasing other environmental impacts, and balanced trade-offs between global warming potential mitigation and broader environmental sustainability.

In conclusion, for all three functional units, the optimal scenario consisted of the combination of LCE2, MSW2, and transportation by electric truck. However, none of the scenarios improved all impact categories simultaneously. Ionising radiation was the only impact category that increased across all three optimal scenarios, with stratospheric ozone depletion and mineral resource scarcity being higher in the optimal trade-off scenario for the cement and building-level assessments, respectively.

## Discussion

### Comparison to existing sector roadmaps

Over the last few years, a great number of documents have been published which establish roadmaps for the improvement of the environmental impacts of cement and concrete. Some UK-relevant documents include Institution of Civil Engineers (2022); Mineral Products Association (2015, 2020); National Highways (2022); UK Green Building Council (2021), while other related publications can be found in Cembureau (n.d.); Concrete New Zealand (2023); Global Cement and Concrete Association (2021); National Highways (2022).

The results obtained in this paper regarding the potential savings when decarbonising the electricity grid align with most reports. For instance, Cembureau (n.d.) states that a 6% reduction in CO_2_-eq emissions can be attained by using renewable energy; Global Cement and Concrete Association (2021) states that a 5% reduction can be obtained by decarbonising electricity, and Mineral Products Association (2020) places this value at 4%.

Concerning transport, (Cembureau, n.d.) determined that 1.5% of cement CO_2_-eq emissions can be reduced by moving to zero-carbon transport modes, while Mineral Products Association (2020) places this value at 7% for cementitious materials. In the remaining documents, while emphasis is given to reducing impacts from transport, no specific value is given to the potential reduction that can be achieved. In fact, it is important to highlight that a key contribution of this study is its transparency in assumptions regarding transportation distances and their impacts for decarbonisation pathways. While some industry roadmaps present ambitious emissions reduction targets, the underlying assumptions for transport distances and vehicle efficiency are often not fully disclosed. In contrast, this study bases its transport modelling on concrete industry reports, ensuring a realistic and data-driven assessment. Average transportation distances for cement and concrete in the UK have been explicitly integrated into the analysis, ensuring a representative evaluation of potential emission reductions.

Alternative fuels also have particular importance in the different reviewed documents, aiming to decrease fossil fuels and increase, among other things, the use of waste and biomass. The Institution of Civil Engineers (2022); Mineral Products Association (2020) reports consider a 16% reduction of CO_2_-eq in the production of Portland cement due to fuel swapping, while International Energy Agency (2018) reports a potential 18% reduction in CO_2_-eq emissions of cement due to fuel switching, and Cembureau (n.d.) aims at increasing the use of alternative fuels to above 90% and states that a saving of 55% of the fuel CO_2_-eq could be achieved (i.e., around a 22% of total CO_2_-eq reduction for cement). These values (between 16 and 22%) are similar to the range obtained in this study (reduction between 20 and 30%).

This article focused on aspects related to electricity, fuel, and transportation. Nevertheless, the documents mentioned before propose and recommend other measures, including improving efficiency in production processes and using alternative materials. For instance, according to the Mineral Products Association (2020), the use of low carbon cements and concrete could reduce CO_2_-eq emissions by 12%. While different recommendations are given in these documents, a common point is the utilisation of carbon capture and storage, albeit each roadmap relies more or less strongly on this technology. In this regard, the CO_2_-eq reduction percentage differs from study to study; for instance, the Mineral Products Association (2020) establishes a 61% reduction in the emissions of cementitious materials and concrete, whereas it is a 42% according to Cembureau (n.d.) and a 48% according to the International Energy Agency (2018).

While this study does not analyse secondary materials in depth, their potential in reducing emissions remains significant. Future work should integrate material substitution strategies, such as supplementary cementitious materials and recycled aggregates, to complement energy-based decarbonisation pathways and provide a more holistic perspective on concrete sustainability.

### Challenges and future outlook

Considering the results, it appears that adopting an electricity mix featuring substantial proportions of nuclear and wind energies, coupled with the use of biomass alongside municipal solid waste (MSW) for kiln fuel, and integrating battery electric trucks, emerges as a promising alternative for reducing CO_2_-eq emissions. However, this optimal scenario for CO_2_-eq reduction might not align with the best outcomes across all impact categories. Specific attention is warranted, particularly regarding the use of nuclear sources in the electricity mix, which could elevate levels of ionising radiation. Additionally, there could be an increased land use associated with the expansion of renewable energy sources, as well as potential releases to compartments other than air from specific fuel sources for the kiln and the production and utilisation of electric batteries.

In this study, it was assumed that all processes happen at a national level. However, not in all contexts will the success of decarbonisation efforts be limited to the country where construction products are manufactured. It is important to consider the fact that a portion of the environmental footprint is associated with the global supply chain, which includes imports and exports (Davis & Caldeira 2010; Li et al. 2022; Zhang et al. 2017). Therefore, reducing emissions and improving sustainability in construction supply chains necessitates not only domestic action but also consideration of elements related to international trade and cooperation.

Achieving fully decarbonised concrete presents significant challenges, particularly due to the process emissions inherent in cement production. According to Habert et al. (2020), the cement industry must implement multiple decarbonisation strategies in parallel, including the adoption of supplementary cementitious materials, alternative fuels, CCS, and improvements in kiln efficiency. While these measures can substantially reduce CO_2_-eq emissions, they also introduce potential trade-offs in other environmental impact categories, such as increased ionising radiation, resource depletion, and potential toxicity issues. Similarly, Georgiades et al. (2023) highlight that prospective life cycle assessments of European cement production indicate that a combination of clinker substitution, alternative fuels, and CCS could lead to an 88% reduction in CO_2_-eq emissions by 2050. However, such reductions depend on the availability of SCMs, the scalability of CCS, and broader shifts in the energy sector. Thus, while net-zero cement is theoretically achievable, it requires balancing CO_2_-eq reductions with the potential burden-shifting to other environmental categories, reinforcing the need for a holistic, multi-criteria assessment approach.

Additionally, some decarbonisation initiatives, such as using waste as a fuel source, require not only technical changes but also a shift in the cultural mindset of the construction sector (Udawatta et al. 2018). Incorporating waste-to-energy practices may challenge traditional construction practices and require a broader sectorial change (Udawatta et al. 2018). This transition involves educating industry stakeholders about the environmental benefits of these practices, promoting the adoption of new technologies, and fostering a culture of sustainability. Initiatives like this call for industry-wide engagement and awareness campaigns to drive acceptance and integration.

While this study provides a detailed assessment of production-oriented decarbonisation strategies—namely low-carbon electricity mixes, fuel substitution in cement kilns, and electrified transport—it does not encompass the full range of mitigation strategies identified in the literature and proposed in industry roadmaps. Notably, other approaches such as the use of alternative materials and innovative design solutions are also widely regarded as important levers to reduce the environmental impacts of the cement and concrete sector (Habert et al. 2020; Müller et al. 2024).

For instance, strategies involving alternative binders (e.g., alkali-activated cements or geopolymers), supplementary cementitious materials (SCMs) such as fly ash or ground granulated blast-furnace slag, or the incorporation of recycled aggregates have been extensively explored in prior studies (Adesina 2020; Georgiades et al. 2023). Design-based interventions, including more efficient structural layouts or the reuse of precast concrete elements (e.g., in modular buildings or reclaimed façade panels), have also shown potential to reduce embodied impacts by minimising material use (Habert et al. 2020). A summary of existing strategies can be found in the Supplementary material.

These strategies were not included in our analysis primarily due to scope limitations, challenges in harmonising data across system boundaries, and the need to focus on a consistent set of scenarios aligned with UK energy transition roadmaps. Additionally, some of the excluded strategies—such as those involving novel binders or CO_2_ mineralisation—still face technical, regulatory, or scalability barriers that make their widespread deployment by 2035–2050 less certain (Cembureau, n.d.); Institution of Civil Engineers, 2022). Others, such as SCMs, have well-documented benefits but are already widely covered in the literature and may face future limitations in availability due to declining production of key industrial by-products (e.g., coal fly ash, blast furnace slag).

Ultimately, the selected focus on low-carbon production pathways reflects both a gap in the current body of multi-scale LCAs and the desire to evaluate strategies that are technologically mature, policy-relevant, and applicable across national contexts. However, integrating these scenarios with complementary material- and design-based strategies would offer a more complete picture. Future research should build on this work by assessing combinations of production, material, and design interventions to better understand potential synergies and trade-offs across environmental impact categories.

The economic viability of decarbonising the electricity grid and deploying electric battery trucks plays a pivotal role in steering the construction sector towards sustainability (Gibson et al. 2024; Owusu et al. 2016). Regarding the electricity grid, transitioning to cleaner energy sources, such as renewable and low-carbon electricity, often involves upfront investment in infrastructure but can lead to long-term economic benefits. Though initial costs may be substantial (Usman et al. 2024), the potential for reduced operational expenses, increased energy efficiency, and the creation of green jobs contribute to a favourable economic outlook. It needs to be noted that governments and industries investing in renewable energy technologies have observed a decline in costs over time, making these alternatives more economically competitive. Furthermore, there exist other challenges related to decarbonisation of energy power sources, such as justice considerations (Abdelbary et al. 2024) or reliability of power supply (Gibson et al. 2024; Shen et al. 2024).

Similarly, the deployment of electric battery trucks presents both challenges and opportunities. While electric vehicles may have higher upfront costs compared to traditional counterparts, the potential for long-term savings through reduced fuel and maintenance expenses can make them economically viable (Mittal et al. 2024; Nawaz et al. 2024). Additionally, governments and businesses are increasingly incentivising the adoption of electric vehicles through subsidies, tax breaks, and regulatory support, further enhancing their economic attractiveness. As technology advances and economies of scale come into play, the costs associated with electric battery trucks are likely to decrease, reinforcing their economic feasibility.

In both cases, it is crucial to consider the broader economic and social impacts, including job creation, reduced healthcare costs associated with air pollution, and the potential for innovation and market growth in the renewable energy and electric vehicle sectors (Owusu et al. 2016). While the initial investment may pose challenges, the long-term economic benefits and the alignment with sustainability goals position decarbonisation of the electricity grid and the deployment of electric battery trucks as economically viable strategies for the construction industry.

## Conclusions

This study presented the results of an environmental LCA investigating the impacts of introducing circularity and decarbonisation strategies in the concrete supply chain. Scenarios of cleaner electricity, fuels, and transportation were evaluated and compared to the business-as-usual case.

The results showed that the scenarios analysed could allow reducing greenhouse gas emissions of cement, concrete, and a reinforced concrete building by up to 35%, 37%, and 29%, respectively. In four impact categories (i.e., stratospheric ozone depletion, ionising radiation, freshwater eutrophication, and land use), significant increases were observed for certain combinations of scenarios. A multi-objective discrete optimisation (MODO) approach was implemented to identify the optimal trade-offs between CO_2_-eq minimisation and overall environmental impact reduction. The results demonstrated that the best-performing scenario for all three functional units (cement, concrete, and building) involved the combination of LCE2, MSW2, and electric truck transport. However, no single scenario improved all impact categories simultaneously, with ionising radiation increasing across all optimal cases. These findings highlight the inherent trade-offs in decarbonisation strategies and reinforce the need for multi-objective approaches in sustainability assessments.

Lastly, to enhance the robustness of this study, future work could incorporate uncertainty analysis to provide insights into the reliability of the findings. Additionally, consequential life cycle assessment (LCA) methods are recommended to assess the anticipated changes in the construction supply chain by 2050, offering a forward-looking perspective on the environmental implications of evolving technologies. The scenarios analysed in this study could be integrated with production processes that integrate circularity strategies, such as the use of supplementary cementitious materials (SCMs) or recycled aggregates, to provide a more comprehensive assessment of decarbonisation pathways in concrete production.

## Supplementary Information

Below is the link to the electronic supplementary material.

ESM1 (PDF 1.62 MB)

## Data Availability

The authors declare that the data supporting the findings of this study are available within the paper and its supplementary information files.

## References

[CR1] Abdelbary AM, Manglicmot L, Kanwhen O, Mohamed AA (2024) Community-centric distributed energy resources for energy justice and decarbonization in dense urban regions. Energy Rep 11:1742–1751. 10.1016/j.egyr.2024.01.023

[CR2] Adesina A (2020) Recent advances in the concrete industry to reduce its carbon dioxide emissions. Environmental Challenges. 10.1016/j.envc.2020.100004

[CR3] Alaux N, Truger B, Hoxha E, Ruschi Mendes Saade M, Passer A (2022) Greenhouse gas reduction strategies for building materials: a reality check with the climate targets. IOP Conf Ser Earth Environ Sci. 10.1088/1755-1315/1078/1/012050

[CR4] Alig, M., Frischknecht, R., Krebs, L., Ramseier, L., & Stolz, P. (2021). *LCA of climate friendly construction materials*. A. f. H. d. S. Z. A. Bundesamt für Energie BFE.

[CR5] Alston SM, Arnold JC (2011) Environmental impact of pyrolysis of mixed WEEE plastics part 2: life cycle assessment. Environ Sci Technol 45(21):9386–9392. 10.1021/es2016654 21939231 10.1021/es2016654

[CR6] Ariyaratne, W. K. H., C. Melaaen, M., & Tokheim, L.-A. (2015). CFD modeling of multi-fuel combustion of coal and meat and bone meal (MBM) in a cement rotary kiln. *International Journal of Modeling and Optimization*,* 5*(6), 353–360. 10.7763/ijmo.2015.V5.488

[CR7] Asphalt Industry Alliance (2021). *The route to net zero: delivering lower emissions*. https://www.asphaltuk.org/wp-content/uploads/asphalt-now-autumn-winter-2021-v11.pdf

[CR8] British Constructional Steelwork Association (2021). *UK structural steelwork: 2050 decarbonisation roadmap*. https://steelconstruction.info/images/3/33/BCSA_2050_Decarbonisation_Roadmap.pdf

[CR9] Backes JG, Del Rosario P, Luthin A, Traverso M (2022) Comparative life cycle assessment of end-of-life scenarios of carbon-reinforced concrete: a case study. Appl Sci. 10.3390/app12189255

[CR10] Barbhuiya S, Kanavaris F, Das BB, Idrees M (2024) Decarbonising cement and concrete production: strategies, challenges and pathways for sustainable development. J Build Eng. 10.1016/j.jobe.2024.108861

[CR11] Bhatty, J. I., Miller, F. M., & Kosmatka, S. H. (2004). *Innovations in Portland cement manufacturing*. Portland Cement Association Skokie, Ill.

[CR12] Boateng, A. A. (2016). Rotary kiln minerals process applications. In *Rotary Kilns* (pp. 231–264). 10.1016/b978-0-12-803780-5.00010-1

[CR13] Boesch ME, Hellweg S (2010) Identifying improvement potentials in cement production with life cycle assessment. Environ Sci Technol 44(23):9143–9149 21047057 10.1021/es100771k

[CR14] Boesch ME, Koehler A, Hellweg S (2009) Model for cradle-to-gate life cycle assessment of clinker production. Environ Sci Technol 43(19):7578–7583. 10.1021/es900036e 19848179 10.1021/es900036e

[CR15] Burnley SJ (2007) A review of municipal solid waste composition in the United Kingdom. Waste Manag 27(10):1274–1285. 10.1016/j.wasman.2006.06.018 17011771 10.1016/j.wasman.2006.06.018

[CR16] Capellán-Pérez I, de Castro C, Arto I (2017) Assessing vulnerabilities and limits in the transition to renewable energies: land requirements under 100% solar energy scenarios. Renewable Sustainable Energy Rev 77:760–782. 10.1016/j.rser.2017.03.137

[CR17] Cembureau (n.d.) Cementing the European green deal: reaching climate neutrality along the cement and concrete value chain by 2050. https://cembureau.eu/media/kuxd32gi/cembureau-2050-roadmap_final-version_web.pdf

[CR18] Cembureau (1999) Best available techniques for the cement industry. The European cement association

[CR19] Chinfon Cement Corporation (2025) Environmental product declaration: CEM I/52.5 N according to EN 197–1:2011 manufactured by Chinfon Cement Corporation

[CR20] Concrete New Zealand (2023) A net-zero carbon concrete industry for Aotearoa New Zealand

[CR21] Cortada Mut MdM, Nørskov LK, Frandsen FJ, Glarborg P, Dam-Johansen K (2015) Circulation of inorganic elements in combustion of alternative fuels in cement plants. Energy Fuels 29(7):4076–4099. 10.1021/ef502633u

[CR22] Cusenza MA, Bobba S, Ardente F, Cellura M, Di Persio F (2019) Energy and environmental assessment of a traction lithium-ion battery pack for plug-in hybrid electric vehicles. J Clean Prod 215:634–649. 10.1016/j.jclepro.2019.01.056 31007414 10.1016/j.jclepro.2019.01.056 PMC6472661

[CR23] Davis SJ, Caldeira K (2010) Consumption-based accounting of CO2 emissions. Proc Natl Acad Sci U S A 107(12):5687–5692. 10.1073/pnas.0906974107 20212122 10.1073/pnas.0906974107 PMC2851800

[CR24] Energy Systems Catapult. (2020). *Innovating to net zero*. https://esc-production-2021.s3.eu-west-2.amazonaws.com/2021%2F09%2FESC_Innovating_to_net_zero_report_A4_AW-single.pdf

[CR25] European Commission (2013) Best Available Techniques (BAT) reference document for the production of cement, lime and magnesium oxide. In: Joint research centre, institute for prospective technological studies.

[CR26] European Standards (2019) Sustainability of construction works - environmental product declarations - core rules for the product category of construction products. In EN 15804+A2

[CR27] Gallego Fernández LM, Navarrete Rubia B, González Falcón R, Vega F (2019) Evaluation of different pretreatment systems for the energy recovery of greenhouse agricultural wastes in a cement plant. ACS Sustain Chem Eng 7(20):17137–17144. 10.1021/acssuschemeng.9b03453

[CR28] García-Gusano D, Garraín D, Dufour J (2017) Prospective life cycle assessment of the Spanish electricity production. Renew Sustain Energy Rev 75:21–34. 10.1016/j.rser.2016.10.045

[CR29] Georgiades M, Shah IH, Steubing B, Cheeseman C, Myers RJ (2023) Prospective life cycle assessment of European cement production. Resour Conserv Recycl. 10.1016/j.resconrec.2023.106998

[CR30] Georgiopoulou M, Lyberatos G (2018) Life cycle assessment of the use of alternative fuels in cement kilns: a case study. J Environ Manage 216:224–234. 10.1016/j.jenvman.2017.07.017 28716294 10.1016/j.jenvman.2017.07.017

[CR31] Gibson A, Makuch Z, Yeganyan R, Tan N, Cannone C, Howells M (2024) Long-term energy system modelling for a clean energy transition in Egypt’s energy sector. Energies. 10.3390/en17102397

[CR32] Global Cement and Concrete Association. (2021). *Concrete future: the GCCA 2050 Cement and Concrete Industry Roadmap for Net Zero Concrete*.

[CR33] Gregory J, AzariJafari H, Vahidi E, Guo F, Ulm FJ, Kirchain R (2021) The role of concrete in life cycle greenhouse gas emissions of US buildings and pavements. Proc Natl Acad Sci U S A. 10.1073/pnas.2021936118 10.1073/pnas.2021936118 PMC8449374 34493648

[CR34] Habert G, Miller SA, John VM, Provis JL, Favier A, Horvath A, Scrivener KL (2020) Environmental impacts and decarbonization strategies in the cement and concrete industries. Nat Rev Earth Environ 1(11):559–573. 10.1038/s43017-020-0093-3

[CR35] Hache E, Simoën M, Seck GS, Bonnet C, Jabberi A, Carcanague S (2020) The impact of future power generation on cement demand: an international and regional assessment based on climate scenarios. Int Econ 163:114–133. 10.1016/j.inteco.2020.05.002

[CR36] Hailemariam A, Erdiaw-Kwasie MO (2022) Towards a circular economy: implications for emission reduction and environmental sustainability. Bus Strateg Environ 32(4):1951–1965. 10.1002/bse.3229

[CR37] Hawkins TR, Singh B, Majeau-Bettez G, Strømman AH (2012) Comparative environmental life cycle assessment of conventional and electric vehicles. J Ind Ecol 17(1):53–64. 10.1111/j.1530-9290.2012.00532.x

[CR38] Institution of Civil Engineers (2022) Low carbon concrete routemap: setting the agenda for a path to net zero

[CR39] International Energy Agency (2018) Technology roadmap: low-carbon transition in the cement industry

[CR40] International Organization for Standardization [ISO] (2006) Environmental management — Life cycle assessment — Requirements and guidelines. In ISO 14044:2006

[CR41] Kabayo J, Marques P, Garcia R, Freire F (2019) Life-cycle sustainability assessment of key electricity generation systems in Portugal. Energy 176:131–142. 10.1016/j.energy.2019.03.166

[CR42] Kadiri M, Ahmadian R, Bockelmann-Evans B, Falconer RA, Kay D (2014) An assessment of the impacts of a tidal renewable energy scheme on the eutrophication potential of the Severn Estuary, UK. Comput Geosci 71:3–10. 10.1016/j.cageo.2014.07.018

[CR43] Knoeri C, Sanyé-Mengual E, Althaus HJ (2013) Comparative LCA of recycled and conventional concrete for structural applications [Article]. Int J Life Cycle Assess 18(5):909–918. 10.1007/s11367-012-0544-2

[CR44] Li R, Zhang J, Krebs P (2022) Global trade drives transboundary transfer of the health impacts of polycyclic aromatic hydrocarbon emissions. Commun Earth Environ 3(1):170. 10.1038/s43247-022-00500-y 35935537 10.1038/s43247-022-00500-y PMC9340739

[CR45] Lieberei J, Gheewala SH (2016) Resource depletion assessment of renewable electricity generation technologies—comparison of life cycle impact assessment methods with focus on mineral resources. Int J Life Cycle Assess 22(2):185–198. 10.1007/s11367-016-1152-3

[CR46] Limleamthong, P., & Guillén-Gosálbez, G. (2018). Combined Use of Bilevel Programming and Multi-objective Optimization for Rigorous Analysis of Pareto Fronts in Sustainability Studies: Application to the Redesign of the UK Electricity Mix. In *28th European Symposium on Computer Aided Process Engineering* (pp. 1099–1104). 10.1016/b978-0-444-64235-6.50192-3

[CR47] Liu Q, Li X, Liu H, Guo Z (2020) Multi-objective metaheuristics for discrete optimization problems: a review of the state-of-the-art. Appl Soft Comput. 10.1016/j.asoc.2020.106382

[CR48] Louis J-N, Allard S, Debusschere V, Mima S, Tran-Quoc T, Hadjsaid N (2018) Environmental impact indicators for the electricity mix and network development planning towards 2050 – a POLES and EUTGRID model. Energy 163:618–628. 10.1016/j.energy.2018.08.093

[CR49] Mahmud MAP, Farjana SH (2022) Comparative life cycle environmental impact assessment of renewable electricity generation systems: a practical approach towards Europe, North America and Oceania. Renew Energy 193:1106–1120. 10.1016/j.renene.2022.05.031

[CR50] Mahmud, M. A. P., & Tasmin, N. (2022). Environmental impact assessment of battery storage. In *Environmental Assessment of Renewable Energy Conversion Technologies* (pp. 277–302). 10.1016/b978-0-12-817111-0.00001-2

[CR51] Marceau, M. L., Nisbet, M. A., & VanGeem, M. G. (2007). *Life Cycle Inventory of Portland Cement Concrete* (Vol. SN3011). Portland Cement Association.

[CR52] Mehrkesh A, Karunanithi AT (2016) Life-cycle perspectives on aquatic ecotoxicity of common ionic liquids. Environ Sci Technol 50(13):6814–6821. 10.1021/acs.est.5b04721 26599072 10.1021/acs.est.5b04721

[CR53] Mikulčić H, Cabezas H, Vujanović M, Duić N (2016) Environmental assessment of different cement manufacturing processes based on emergy and ecological footprint analysis. J Clean Prod 130:213–221. 10.1016/j.jclepro.2016.01.087

[CR54] Mineral Products Association (2015) UK cement industry 2050 greenhouse gas strategy. https://cement.mineralproducts.org/documents/MPA_Cement_2050_Strategy.pdf

[CR55] Mineral Products Association (2020) UK concrete and cement industry roadmap to beyond net zero

[CR56] Mineral Products Association (2023) MPA cement stats data - quarterly cementitious. https://cement.mineralproducts.org/MPACement/media/Cement/Industry-Statistics/2023/2023-07-11_Quarterly_cementitious.pdf

[CR57] Mittal G, Garg A, Pareek K (2024) A review of the technologies, challenges and policies implications of electric vehicles and their future development in India. Energy Storage. 10.1002/est2.562

[CR58] Moore-O’Leary KA, Hernandez RR, Johnston DS, Abella SR, Tanner KE, Swanson AC, Kreitler J, Lovich JE (2017) Sustainability of utility-scale solar energy – critical ecological concepts. Front Ecol Environ 15(7):385–394. 10.1002/fee.1517

[CR59] Moultak, M., Lutsey, N., & Hall, D. (2017). Transitioning to zero-emission heavy-duty freight vehicles.

[CR60] MPA The Concrete Centre. (2022). Local material – UK locally-sourced material. In.

[CR61] MPA The Concrete Centre. (2023). Concrete Industry Sustainability Performance Report. 14th report: 2020 performance data. In.

[CR62] MPA, Cinar Ltd, & VDZ gGmbH. (2019). *Options for switching UK cement production sites to near zero CO2 emission fuel: Technical and financial feasibility*. T. 1674/10/2018.

[CR63] Müller A, Harpprecht C, Sacchi R, Maes B, van Sluisveld M, Daioglou V, Šavija B, Steubing B (2024) Decarbonizing the cement industry: findings from coupling prospective life cycle assessment of clinker with integrated assessment model scenarios. J Clean Prod. 10.1016/j.jclepro.2024.141884

[CR64] National Grid ESO (2020) Future energy scenarios. https://www.nationalgrideso.com/future-energy/future-energy-scenarios

[CR65] National Highways. (2022). *Net zero highways: our zero carbon roadmap for concrete, steel and asphalt*.

[CR66] Nawaz MU, Umar S, Qureshi MS (2024) Life cycle analysis of solar-powered electric vehicles: environmental and economic perspectives. International Journal of Advanced Engineering Technologies and Innovations 1(3):96–115

[CR67] Nwonu DC, Josa I, Bernal SA, Velenturf APM, Hafez H (2025) Circrete: a multi-criteria performance-based decision support framework for end-of-life management of concrete. Renew Sustain Energy Rev. 10.1016/j.rser.2024.115232

[CR68] Office for National Statistics (2023) Material flows account for the United Kingdom

[CR69] Office for National Statistics (2024) Dataset(s): material flow accounts. https://www.ons.gov.uk/economy/environmentalaccounts/datasets/ukenvironmentalaccountsmaterialflowsaccountunitedkingdom/current

[CR70] Onyelowe KC, Ebid AM, Riofrio A, Soleymani A, Baykara H, Kontoni D-PN, Mahdi HA, Jahangir H (2022) Global warming potential-based life cycle assessment and optimization of the compressive strength of fly ash-silica fume concrete; environmental impact consideration. Front Built Environ. 10.3389/fbuil.2022.992552

[CR71] Owusu, P. A., Asumadu-Sarkodie, S., & Dubey, S. (2016). A review of renewable energy sources, sustainability issues and climate change mitigation. *Cogent Eng.*,* 3*(1). 10.1080/23311916.2016.1167990

[CR72] Panesar DK, Seto KE, Churchill CJ (2017) Impact of the selection of functional unit on the life cycle assessment of green concrete. Int J Life Cycle Assess 22(12):1969–1986. 10.1007/s11367-017-1284-0

[CR73] Patni N, Shah P, Agarwal S, Singhal P (2013) Alternate strategies for conversion of waste plastic to fuels. ISRN Renewable Energy 2013:1–7. 10.1155/2013/902053

[CR74] Petek Gursel, A. (2014). *Life-cycle assessment of concrete: decision-support tool and case study application* University of California, Berkeley].

[CR75] Quéheille E, Ventura A, Saiyouri N, Taillandier F (2022) A life cycle assessment model of end-of-life scenarios for building deconstruction and waste management. J Clean Prod. 10.1016/j.jclepro.2022.130694

[CR76] Readymix Industries Ltd. (2022). Environmental Product Declaration: C25/30 READY-MIX CONCRETE Programme: by Readymix Industries (Israel) Ltd.

[CR77] Rihner, M., Hafez, H., Walkley, B., Purnell, P., & Drewniok, M. (2025). Thousand cuts: a realistic route to decarbonisation the UK cement and concrete sector by 2050. *Sustain. Prod. Consum.*10.1016/j.spc.2025.06.010

[CR78] Rogelj J, Schaeffer M, Meinshausen M, Knutti R, Alcamo J, Riahi K, Hare W (2015) Zero emission targets as long-term global goals for climate protection. Environ Res Lett. 10.1088/1748-9326/10/10/105007

[CR79] Roh S, Kim R, Park W-J, Ban H (2020) Environmental evaluation of concrete containing recycled and by-product aggregates based on life cycle assessment. Appl Sci. 10.3390/app10217503

[CR80] Sathre R, Gustavsson L (2023) Lifecycle climate impact and primary energy use of electric and biofuel cargo trucks. GCB Bioenergy 15(4):508–531. 10.1111/gcbb.13034

[CR81] Seyler C, Hellweg S, Monteil M, Hungerbühler K (2004) Life cycle inventory for use of waste solvent as fuel substitute in the cement industry - a multi-input allocation model (11 pp). The International Journal of Life Cycle Assessment 10(2):120–130. 10.1065/lca2004.08.173

[CR82] Shen B, Hove A, Hu J, Dupuy M, Bregnbæk L, Zhang Y, Zhang N (2024) Coping with power crises under decarbonization: the case of China. Renew Sustain Energy Rev. 10.1016/j.rser.2024.114294

[CR83] Solomon S (1999) Stratospheric ozone depletion: a review of concepts and history. Rev Geophys 37(3):275–316

[CR84] Speth D, Funke SÁ (2021) Comparing options to electrify heavy-duty vehicles: findings of German pilot projects. World Electr Veh J. 10.3390/wevj12020067

[CR85] Stewart R, Niero M (2018) Circular economy in corporate sustainability strategies: a review of corporate sustainability reports in the fast-moving consumer goods sector. Bus Strateg Environ 27(7):1005–1022. 10.1002/bse.2048

[CR86] Tangthieng C (2017) Inventory-based analysis of greenhouse gas emission from the cement sector in Thailand. Eng J 21(5):125–136. 10.4186/ej.2017.21.5.125

[CR87] United Nations Environment (2021) 2021 global status report for buildings and construction: towards a zero‑emission, efficient and resilient buildings and construction sector. www.globalabc.org

[CR88] Udawatta N, Zuo J, Chiveralls K, Yuan H, George Z, Elmualim A (2018) Major factors impeding the implementation of waste management in Australian construction projects. J Green Build. 10.3992/1943-4618.13.3.101

[CR89] UK Department for Energy Security and Net Zero. (2023). *National Statistics - Energy Trends: Fuel used in electricity generation and electricity supplied*. https://www.gov.uk/government/statistics/electricity-section-5-energy-trends

[CR90] UK Green Building Council (2021) Net zero whole life carbon roadmap: a pathway to net zero for the UK built environment

[CR91] Usman, F. O., Ani, E. C., Ebirim, W., Montero, D. J. P., Olu-lawal, K. A., & Ninduwezuor-Ehiobu, N. (2024). Integrating renewable energy solutions in the manufacturing industry: challenges and opportunities: a review. *Engineering Science & Technology Journal*,* 5*(3), 674–703. 10.51594/estj/v5i3.865

[CR92] Verma P, Zare A, Jafari M, Bodisco TA, Rainey T, Ristovski ZD, Brown RJ (2018) Diesel engine performance and emissions with fuels derived from waste tyres. Sci Rep 8(1):2457. 10.1038/s41598-018-19330-0 29410435 10.1038/s41598-018-19330-0 PMC5802751

[CR93] White C (2020) Alkali-activated materials: the role of molecular-scale research and lessons from the energy transition to combat climate change. RILEM Tech Lett 4:110–121. 10.21809/rilemtechlett.2019.98

[CR94] Yuan J, Xu X, Huang B, Li Z, Wang Y (2023) Regional planning of solar photovoltaic technology based on LCA and multi-objective optimization. Resour Conserv Recycling. 10.1016/j.resconrec.2023.106977

[CR95] Zhang, X., & Bauer, C. (2018). Life Cycle Assessment (LCA) of Nuclear Power in Switzerland. In. Paul Scherrer Institut (PSI), Villigen, Switzerland.

[CR96] Zhang Q, Jiang X, Tong D, Davis SJ, Zhao H, Geng G, Feng T, Zheng B, Lu Z, Streets DG, Ni R, Brauer M, van Donkelaar A, Martin RV, Huo H, Liu Z, Pan D, Kan H, Yan Y, Lin J, He K, Guan D (2017) Transboundary health impacts of transported global air pollution and international trade. Nature 543(7647):705–709. 10.1038/nature21712 28358094 10.1038/nature21712

