# Supplementary material for: Decarbonising concrete production in the United Kingdom: a life cycle assessment of energy, transport, and fuel transitions
Source: Int J Life Cycle Assess. 2025 Oct 6;30(12):3515–35. doi: 10.1007/s11367-025-02537-5 (PMC12864233; doi:10.1007/s11367-025-02537-5)
Supplement: Supplementary file 1 — (PDF 1.62 MB) [file 11367_2025_2537_MOESM1_ESM.pdf]

Decarbonising concrete production in the United Kingdom: a life cycle assessment of energy, transport, and fuel transitions

Supplementary material

DOI: 10.1007/s11367-025-02537-5

Irene Josa[a*], Aiduan Borrion[b]

[a]The Bartlett School of Sustainable Construction, University College London, United Kingdom

[b]Department of Civil, Environmental & Geomatic Engineering, University College London, United Kingdom

[*] i.josa@ucl.ac.uk

Contents

S1. Background to the selection of scenarios 3

S1. Inventory data 5

S2. Validation of the model 9

S2. Results 11

References 31

List of tables

Table 1 Decarbonisation strategies assessed and excluded. 3

Table 2 Reference distances 5

Table 3 Cement inventory data. 5

Table 4 Cement raw material quantities by kiln type (unit: kg/ton cement). Source: (Marceau et al., 2007). 5

Table 5 Concrete composition. 6

Table 6 Conveying distance and technology 6

Table 7 Conveying technology and associated energy. 6

Table 8 Crushing technology and associated energy. 6

Table 9 Kiln fuel preparation associated electricity. 7

Table 10 Electricity use for milling and grinding technology options. 7

Table 11 Electricity use for concrete mixing and batching technology options. 7

Table 12 Electricity use and water consumption for clinker cooling technology options. 7

Table 13 Electricity use and particulate matter emission for particulate matter control technology. 8

Table 14 Technology options for the production processes. 8

Table 15 Building design. 9

Table 16 Building inventory – floors. 9

Table 17 Building inventory – foundation. 9

Table 18 Results of the cleaner electricity scenarios for the production of 1 ton of cement (part 1). 13

Table 19 Results of the cleaner electricity scenarios for the production of 1 ton of cement (part 2). 13

Table 20 Results of the cleaner electricity scenarios for the production of 1 m^3 of concrete (part 1)...13
Table 21 Results of the cleaner electricity scenarios for the production of 1 m^3 of concrete (part 2)...14
Table 22 Results of the cleaner electricity scenarios for the production of 1 m^2 of building (part 1)...14
Table 23 Results of the cleaner electricity scenarios for the production of 1 m^2 of building (part 2)...15
Table 24 Results of the cleaner fuels scenarios for the production of 1 ton of cement (part 1). 15
Table 25 Results of the cleaner fuels scenarios for the production of 1 ton of cement (part 2). 16
Table 26 Results of the cleaner transport scenarios for the production of 1 ton of cement (part 1)......16
Table 27 Results of the cleaner transport scenarios for the production of 1 ton of cement (part 2)......17
Table 28 Results of the cleaner transport scenarios for the production of 1 m^3 of concrete (part 1).....17
Table 29 Results of the cleaner transport scenarios for the production of 1 m^3 of concrete (part 2).....17
Table 30 Results of the cleaner transport scenarios for the production of 1 m^2 of building (part 1).....18
Table 31 Results of the cleaner transport scenarios for the production of 1 m^2 of building (part 2).....18
Table 32 Results of the combined scenarios for the production of 1 ton of cement (part 1)................19
Table 33 Results of the combined scenarios for the production of 1 ton of cement (part 2)................21
Table 34 Results of the combined scenarios for the production of 1 m^3 of concrete (part 1)................22
Table 35 Results of the combined scenarios for the production of 1 m^3 of concrete (part 2)................24
Table 36 Results of the combined scenarios for the production of 1 m^2 of building (part 1)................26
Table 37 Results of the combined scenarios for the production of 1 m^2 of building (part 2)................28
Table 38 Comparison of scenario results with industry roadmap targets...30

List of figures

Figure 1 Structural components considered in the LCA analysis (foundations, columns, beams, and slabs). Non-structural elements such as interior finishes and mechanical systems are excluded from the study. 8
Figure 2 Relative results of the LCA for 1 ton of cement. 9
Figure 3 Relative results of the LCA for m^3 of concrete. 10
Figure 4 Relative results of the LCA for m^2 of building. 10
Figure 5 Results of all scenarios on the impacts of cement (FU: 1 ton), concrete (FU: 1 m^3), and building (FU: 1 m^2)...11

S1. Background to the selection of scenarios

Habert et al. (2020) reviewed a comprehensive range of decarbonisation strategies for the cement and concrete industries. These strategies can be broadly categorised into three groups: (i) low-carbon production processes (e.g., energy efficiency improvements, fuel substitution), (ii) alternative materials (e.g., supplementary cementitious materials (SCMs), alkali-activated binders, recycled aggregates), and (iii) alternative design strategies (e.g., optimised structural design, reuse of concrete components).

In this study, the strategies assessed were selected from the first category—low-carbon production processes—based on three main considerations: (1) this area is relatively underexplored in detailed multi-scale LCAs despite its importance for the reduction of impacts, (2) the selected strategies align with current UK policy targets and national decarbonisation roadmaps, increasing their relevance and potential for near-term implementation, and (3) their level of technological maturity allow for an expected deployment within the short to medium term.

To ensure transparency regarding the scope of the assessment, Table 1 summarises the key strategies considered and those excluded from this study, along with justifications based on factors such as technological maturity, data availability, and relevance to the study's geographical and temporal boundaries.

Table 1 Decarbonisation strategies assessed and excluded.

Strategy	Description	Maturity	Reason for inclusion or exclusion	Relevant references
**Low carbon electricity mix**	Transition to a national electricity grid with substantial proportions of renewable and low-carbon energies to power all stages of production.	High	Included: Electricity is a core input for grinding, conveying, and other plant operations. Decarbonising the grid is a foundational strategy in all major roadmaps. Transparent, quantitative future scenarios for the UK grid are available from official sources, enabling robust and policy-relevant LCA modelling.	Cembureau (n.d.); Georgiades et al. (2023); Global Cement and Concrete Association (2021); Habert et al. (2020); Mineral Products Association (2015)
**Fuel substitution in kilns**	Replacing carbon-intensive fossil fuels (e.g., coal) with alternative fuels like municipal solid waste (MSW), biomass, and waste-derived fuels in cement kilns.	Medium–High	Included: This strategy directly addresses the ~40% of cement emissions from fuel combustion. It is a central pillar of all industry decarbonisation plans and has a high TRL. Comprehensive data on various fuel mixes and their impacts were available for analysis.	Cembureau (n.d.); Global Cement and Concrete Association (2021); Habert et al. (2020)
**Transport electrification**	Utilizing battery electric trucks (BETs) for the transportation of raw materials to plants, cement to concrete batchers, and concrete to construction sites.	Medium–High	Included: Transport is a key component of the concrete supply chain's carbon footprint (A4 module), targeted for decarbonisation in industry roadmaps. Data was available to model the shift to BETs and assess the strategy's robustness across different transport distances and supply chain levels.	Institution of Civil Engineers (2022); Mineral Products Association (2015)
**Clinker substitution**	Incorporating Supplementary Cementitious Materials (SCMs) like fly ash, ground granulated blast-furnace slag (GGBS), or limestone filler to reduce the Portland cement clinker content of the final product.	High	Excluded: This is a vital but well studied strategy in prior LCAs. The study aimed to focus on other, less-quantified levers. Furthermore, future availability of GGBS and fly ash is constrained by the decarbonisation of the steel and coal industries, creating uncertainty for long-term generalised scenarios.	Georgiades et al. (2023); Habert et al. (2020); Institution of Civil Engineers (2022); Müller et al. (2024)
**Recycled aggregates**	Use of crushed and processed concrete from	Medium–High	Excluded: This is a vital but well studied strategy in prior LCAs. The	Marinković et al. (2023)

	demolition waste to replace virgin sand and gravel in new concrete mixes.		study aimed to focus on other, less-quantified levers.	
**Carbon capture & storage (CCS)**	Capturing CO_2 emissions from cement plant flues using technologies like amine scrubbing or oxy-fuel combustion and storing them permanently in geological formations.	Low–Medium	Excluded: While interesting for deep decarbonisation and a major component of 2050 roadmaps, CCS has a low TRL for widespread, commercial cement applications. It faces major deployment barriers, including high costs, policy gaps, and the need for new CO_2 transport infrastructure. Commercial feasibility is not expected until post-2030, and robust operational life cycle data is not yet available.	Cembureau (n.d.); Global Cement and Concrete Association (2021); Habert et al. (2020); Institution of Civil Engineers (2022)
**Alternative binders**	Developing and using novel binders (e.g., geopolymers, alkali-activated cements) or alternative clinker chemistries (e.g., Belite-Ye'elimite-Ferrite) that have lower process emissions than Portland cement.	Low	Excluded: These technologies are not yet market-scalable. Deployment is limited by the availability of specific raw materials (e.g., sodium silicate, bauxite), inconsistent performance data, and a lack of inclusion in mainstream construction standards. This makes robust LCA modelling for a national scenario challenging at present.	Cembureau (n.d.); Habert et al. (2020); Institution of Civil Engineers (2022)
**CO_2 mineralisation/ carbonation**	Utilising captured CO_2 to cure concrete or create synthetic aggregates/SCMs (active carbonation). Also includes the natural, passive absorption of CO_2 by concrete during its use and end-of-life phases (passive carbonation).	Low	Excluded: Active CO_2 mineralisation and curing are niche technologies with limited commercial deployment and scarce LCI data for a robust, large-scale assessment. Passive carbonation is an important process but occurs primarily in the use and end-of-life phases, which are outside this study's production-focused system boundary.	Institution of Civil Engineers (2022)
**Waste heat recovery**	Capturing high-temperature exhaust heat from the kiln process and converting it into electricity to power the cement plant, reducing demand for grid electricity.	High	Excluded: This is an established energy efficiency measure rather than a primary decarbonisation lever. Its impact is implicitly accounted for within the broader low carbon electricity mix and fuel efficiency scenarios, which model the overall energy demand and carbon intensity of power consumed by the plant.	Karellas et al. (2013)
**Design/material efficiency**	Optimising structural designs (e.g., using voids, coffers) to reduce overall material quantities, or optimising concrete mix designs to minimise cement content.	High	Excluded: This is a critical but design-phase strategy. Its impact is highly project-specific and dependent on engineering decisions, placing it beyond the scope of this process-based LCA focused on the production and transport impacts of a standard functional unit of material.	Afzal et al. (2020)
**Reuse of concrete elements**	Direct reuse of precast or in-situ concrete structural components from deconstructed buildings in new projects.	Low	Excluded: Data on large-scale direct reuse is limited, and feasibility is highly dependent on regional demolition practices and specific project characteristics.	–
**Demand-side reduction**	Strategies aimed at reducing overall construction volumes or influencing user behaviour to lower demand for new construction.	N/A	Excluded: This is an end-of-life and design-phase circularity strategy that falls outside the study's scope. Data on large-scale direct reuse is limited, and feasibility is highly dependent on regional demolition practices, element standardisation, and recertification processes.	–

S1. Inventory data

Table 2 Reference distances.

Transported element	Transport stages		Distances (km)		Reference
	From	To	BAU	Alternative	
Cement production					
Cement raw materials	Quarry	Cement plant	50	150	MPA The Concrete Centre (2023)
Concrete production					
Cement	Cement plant	Concrete plant	42	126	MPA The Concrete Centre (2023)
Additive (plasticiser)	Additive plant	Concrete plant	42	126	MPA The Concrete Centre (2023)
Aggregates	Quarry	Concrete plant	42	126	MPA The Concrete Centre (2023)
Building					
Concrete	Concrete plant	Construction site	29	87	MPA The Concrete Centre (2023)
Steel	Steel plant	Construction site	29	87	MPA The Concrete Centre (2023)

The cement inventory is shown in Table 3. For the gypsum to clinker mass ratio, the ratios of 5/95, 7/93, 3/97 were used, where each corresponds the average, maximum, and minimum gypsum contents versus average, minimum, and maximum clinker contents, respectively.

Table 3 Cement inventory data.

Cement raw material	Weight	Unit
Clinker	341.25	kg/m^3 concrete
Gypsum	18.42	kg/m^3 concrete

The amount of raw meal varies with the kiln technology, as shown in Table 4.

Table 4 Cement raw material quantities by kiln type (unit: kg/ton cement). Source: (Marceau et al., 2007).

Cement raw material	Wet kiln	Long dry kiln	Preheater kiln	Preheater/ Precalciner kiln
Quarried materials				
Limestone	1228.00	1262.00	1137.00	1127.00
Cement rock, marl	269.00	131.00	70.00	249.00
Shale	65.00	13.00	23.00	68.00
Clay	62.00	35.00	100.00	54.00
Sand	57.00	36.00	36.00	38.00
Slate	7.00	0.00	0.00	0.00
Iron, iron ore	9.00	15.00	16.00	14.00
Industrial by-products				
Bottom ash	10.00	19.00	5.00	9.00
Fly ash	17.00	23.00	7.00	12.00
Blast furnace slag	25.00	38.00	34.00	9.00
Foundry sand	0.00	11.00	5.00	3.00
Others	3.00	29.00	59.00	23.00

The details of the concrete composition utilised in the study are shown in Table 5. Major concrete raw materials include cement, water, fine aggregates, and coarse aggregates. Typically, a concrete mixture is about 7-15% cement, 60-80% aggregates, and 15-20% water by weight.

Table 5 Concrete composition.

Concrete components	Weight	Unit
Main components		
Cement	350	kg/m^3 concrete
Water	175	kg/m^3 concrete
Fine aggregates	715	kg/m^3 concrete
Coarse aggregates	1080	kg/m^3 concrete
Admixtures		
Superplasticiser	0.06	kg/m^3 concrete

Table 6 Conveying distance and technology.

Conveyed product	Distance conveyed	Unit	Conveying technology
Cement production			
Raw meal	50	m	Belt
Ground meal	20	m	Belt
Blended meal	20	m	Belt
Clinker	20	m	Belt
Cooled clinker	20	m	Belt
Blended cement	20	m	Belt
Main components			
Fine aggregates	20	m	Belt
Coarse aggregates	20	m	Belt

Table 7 Conveying technology and associated energy.

Conveying technology	Energy	Unit	Reference
Airlift (pneumatic conveying)	1.65E-05	kWh/kg-m	Commercial data (Claudius Peters)
Belt	3.24E-06	kWh/kg-m	Commercial data (Agico Cement)
Bucket elevator	6.74E-06	kWh/kg-m	Commercial data (Agico Cement)
Dense phase pump	5.90E-06	kWh/kg-m	Commercial data (SDDOM)
Screw pump	1.20E-06	kWh/kg-m	Petek Gursel (2014)

Table 8 Crushing technology and associated energy.

Conveying technology	Energy	Unit	Reference
Cone crusher	1.11	kWh/ton	Commercial data (Sinonine)
Hammer crusher	1.62	kWh/ton	Commercial data (Sinonine, Chaeng)
Impact crusher	1.10	kWh/ton	Commercial data (Sinonine, Chaeng)
Jaw crusher	0.86	kWh/ton	Commercial data (Sinonine)

Table 9 Kiln fuel preparation associated electricity.

Fuel	Energy	Unit	Reference
Conventional fuels			
Bituminous coal	40	kWh/ton	Boesch et al. (2009)
Lignite coal	35	kWh/ton	Boesch et al. (2009)
Distillate (diesel or light) fuel oil	0	kWh/ton	
Petroleum coke	45	kWh/ton	Boesch et al. (2009)
Residual fuel (heavy) oil	3	kWh/ton	Bhatty et al. (2004); Boesch et al. (2009)
Natural gas	0	kWh/ton	
Biomass	25	kWh/ton	Boesch and Hellweg (2010)
Waste			
Waste oil			
Waste solvent	3	kWh/ton	Boesch et al. (2009)
Waste tire	3	kWh/ton	Boesch and Hellweg (2010)
Waste paper	45	kWh/ton	Boesch and Hellweg (2010)
Waste plastics	25	kWh/ton	Boesch and Hellweg (2010)
Waste sewage sludge	43	kWh/ton	Boesch and Hellweg (2010)

Table 10 Electricity use for milling and grinding technology options.

Milling and griding technology	Energy	Unit	Reference
Ball mill	36	kWh/ton	**Marceau et al. (2007)**
Horizontal roller mill	25.5	kWh/ton	**Marceau et al. (2007)**
Roller press	27.5	kWh/ton	**Marceau et al. (2007)**
Tube mill	34.675	kWh/ton	**Marceau et al. (2007)**
Vertical roller mill	27.1	kWh/ton	**Marceau et al. (2007)**

Table 11 Electricity use for concrete mixing and batching technology options.

Mixing and batching technology	Energy	Unit	Reference
Compact plant	0.723	kWh/m^3	Commercial data (Meka Global)
Mobile plant	0.758	kWh/m^3	Commercial data (Meka Global)
Stationary plant	0.783	kWh/m^3	Commercial data (Meka Global)

Table 12 Electricity use and water consumption for clinker cooling technology options.

Mixing and batching technology	Electricity (kWh ton cement)	Water consumption (m^3/ton clinker)	Reference
Grate cooler (recirculating excess air)	9.025	0	Cembureau (1999)
Planetary (satellite) cooler	0.95	0.03	Cembureau (1999)
Reciprocating grate cooler (conventional)	4.75	0	Cembureau (1999)
Reciprocating grate cooler (modern)	5.7	0	Cembureau (1999)
Rotary (tube) cooler	3.563	0.03	Cembureau (1999)
Vertical gravity cooler with planetary cooler	8.075	0	Cembureau (1999)

Table 13 Electricity use and particulate matter emission for particulate matter control technology.

Mixing and batching technology	Electricity (kWh ton cement)	PM (kg/ton cement)	Reference
Electrostatic precipitators (ESP)	1.664	0.00005	**Marceau et al. (2007)**
Fabric filter (FF)	1.902	0.00006	**Marceau et al. (2007)**

Table 14 Technology options for the production processes.

Process	Tecnology/type of process
Cement production	
Raw materials crushing	Impact crusher
Raw materials prehomogenisation	Dry process
Raw materials griding	Ball mill
Raw meal blending/prehomogenisation	Dry process
Pyroprocessing	Preheater/precalciner kiln
Clinker cooling	Rotary (tube) cooler
Finish milling, grinding, blending	Tube mill
Clinker cooling PM control	Fabric filter
Aggregate production	
Primary crushing	Cone crusher
Secondary crushing	Cone crusher
Screening	Horizontal screening

The building analysed in the study was a 5-story reinforced concrete building, which was designed using the software CYPE. The code considered for the design was Eurocode. The building design is shown in Figure 1, and details on the design and the inventory are presented in Table 15, Table 16, and Table 17.

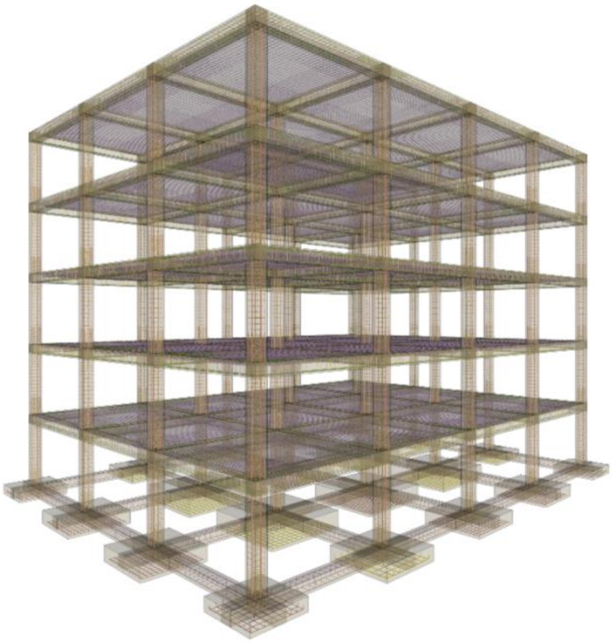

Figure 1 Structural components considered in the LCA analysis (foundations, columns, beams, and slabs). Non-structural elements such as interior finishes and mechanical systems are excluded from the study.

Table 15 Building design.

	Quantity	Unit
Floors	4	Number
Story height	3	m
Bays in transversal direction	3	m
Distance between bays in transversal direction	5	m
Bays in longitudinal direction	4	m
Distance between bays in longitudinal direction	5	m

Table 16 Building inventory – floors.

Floor	Flat slabs		Beams		Columns	
	Concrete (m^3)	Steel (kg)	Concrete (m^3)	Steel (kg)	Concrete (m^3)	Steel (kg)
Floor 1	38.09	4623	25.38	1553	8.32	922
Floor 2	38.09	4610	25.38	1498	8.32	922
Floor 3	38.09	4546	25.38	1467	8.32	922
Floor 4	38.09	4531	25.38	1404	8.32	922
Floor 5	38.09	4400	25.38	1395	8.32	800

Table 17 Building inventory – foundation.

	Pad footings		Tie beams	
	Concrete (m^3)	Steel (kg)	Concrete (m^3)	Steel (kg)
Foundation	73.76	1847	12.05	858

S2. Validation of the model

The BAU model was validated by comparing the results against existing literature. Anderson and Moncaster (2020) reviewed LCAs performed for cements, aggregates, and concrete mixes. For CEM I, the global warming potential of the studies they analysed ranged between 680 and 970 kg CO_2-eq per ton of cement. The BAU results for the cement in this study is of 891 kg CO_2-eq per ton of cement, which falls within the range reviewed by Anderson and Moncaster (2020).

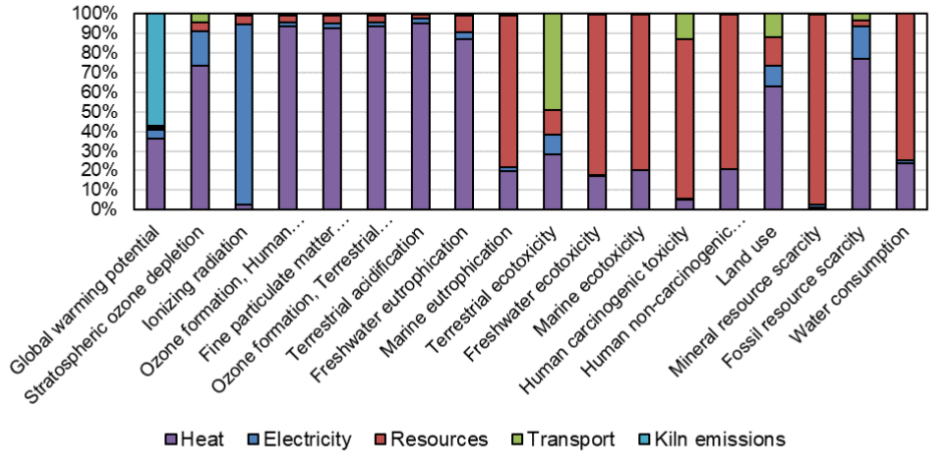

Figure 2 Relative results of the LCA for 1 ton of cement.

In their review, the range of global warming potential for ready mix concrete with 28-day strength of 25 MPa was found to be between 150 and 370 kg CO_2-eq per m^3 of concrete. The BAU results in this study were of 336 kg CO_2-eq per m^3 of concrete, which is consistent with the range.

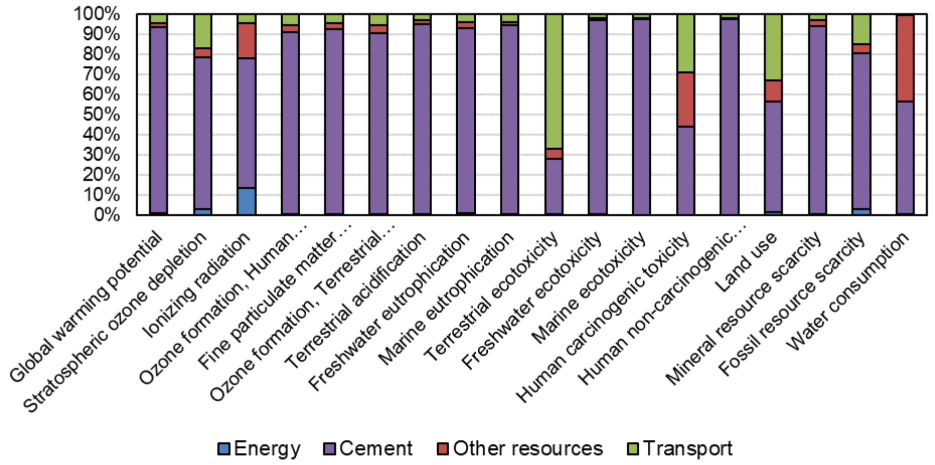

Figure 3 Relative results of the LCA for m^3 of concrete.

Lastly, at a building level, Gibbons et al. (2022) wrote that the embodied carbon corresponding to the business-as-usual construction of a building typically ranges between 150-400 $kgCO_2$-eq/m^2. In this study, the results obtained were of 141 $kgCO_2$-eq per m^2 of the building. This is slightly lower than the above range, but the reason for this is the consideration of the structural elements solely, namely foundations, pillars, and slabs.

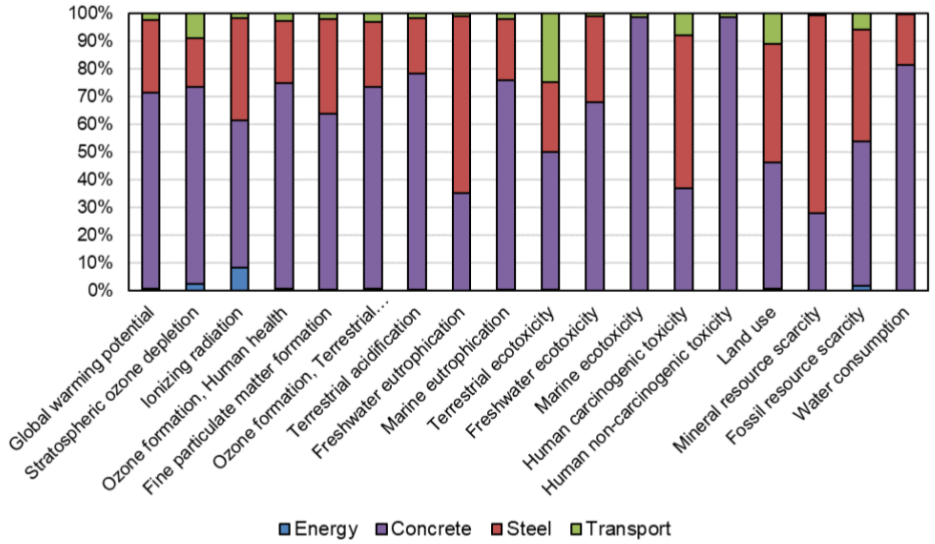

Figure 4 Relative results of the LCA for m^2 of building.

S2. Results

The results of the impact categories when considering all scenarios are presented in Figure 5, which shows the percentage change with respect to the BAU scenario for cement, concrete, and building impacts in each impact category. In this analysis, 'all scenarios' refers to the combined scenario, which incorporates all previously assessed modifications to electricity mix, fuel substitution, and transport electrification. This approach allows for evaluating the cumulative benefits of decarbonisation measures beyond individual scenario outcomes.

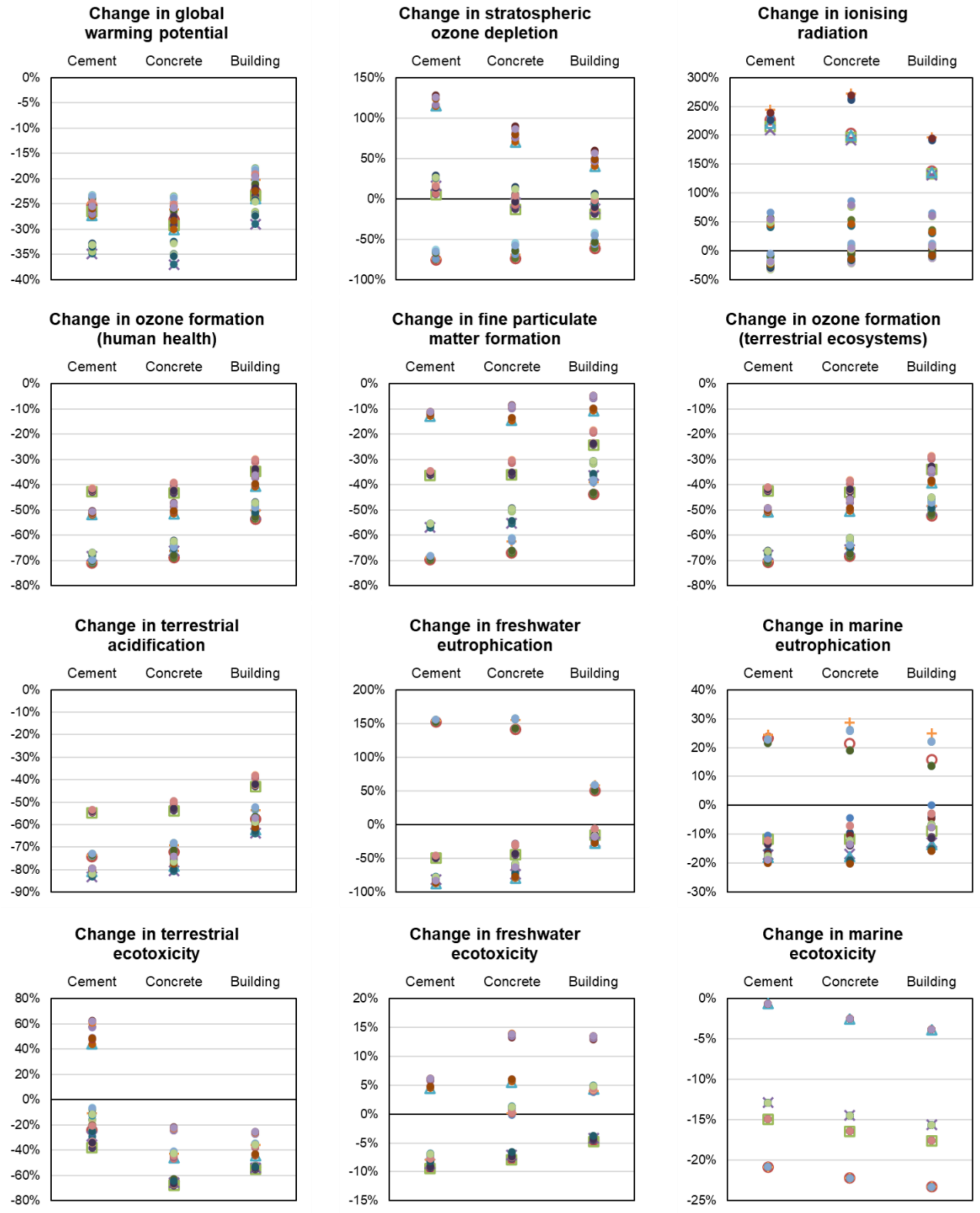

Figure 5 Results of all scenarios on the impacts of cement (FU: 1 ton), concrete (FU: 1 m^3), and building (FU: 1 m^2).

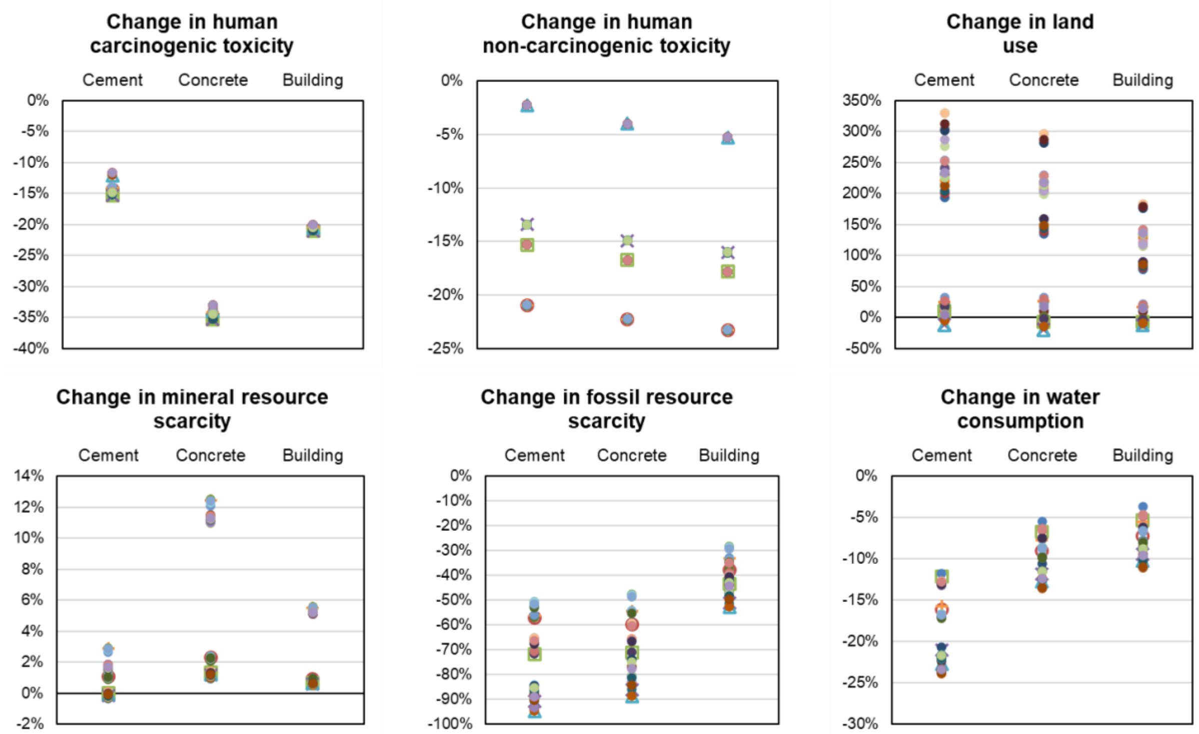

Figure 5 (continued) Results of all scenarios on the impacts of cement (FU: 1 ton), concrete (FU: 1 m^3), and building (FU: 1 m^2).

Table 18 Results of the cleaner electricity scenarios for the production of 1 ton of cement (part 1).

Scenario	Global warming potential	Stratospheric ozone depletion	Ionizing radiation	Ozone formation, Human health	Fine particulate matter formation	Ozone formation, Terrestrial ecosystems	Terrestrial acidification	Freshwater eutrophication	Marine eutrophication
	kg CO_2 eq	kg CFC11 eq	kBq Co-60 eq	kg NOx eq	kg PM2.5 eq	kg NOx eq	kg SO2 eq	kg P eq	kg N eq
BAU UK 2022	8.91E+02	1.63E-04	1.82E+01	2.57E+00	9.19E-01	2.59E+00	3.00E+00	8.63E-02	3.10E-02
LCE1	8.52E+02	1.46E-04	5.88E+01	2.53E+00	9.02E-01	2.55E+00	2.94E+00	8.43E-02	3.15E-02
LCE2	8.52E+02	1.46E-04	2.70E+01	2.53E+00	9.03E-01	2.55E+00	2.94E+00	8.44E-02	3.10E-02
LCE3	8.67E+02	1.61E-04	1.38E+01	2.55E+00	9.10E-01	2.57E+00	2.96E+00	8.57E-02	3.10E-02
LCE4	8.66E+02	1.64E-04	1.31E+01	2.55E+00	9.10E-01	2.57E+00	2.96E+00	8.55E-02	3.10E-02
LCE5	8.65E+02	1.61E-04	1.45E+01	2.55E+00	9.09E-01	2.57E+00	2.96E+00	8.56E-02	3.10E-02

Table 19 Results of the cleaner electricity scenarios for the production of 1 ton of cement (part 2).

Scenario	Terrestrial ecotoxicity	Freshwater ecotoxicity	Marine ecotoxicity	Human carcinogenic toxicity	Human non-carcinogenic toxicity	Land use	Mineral resource scarcity	Fossil resource scarcity	Water consumption
	kg 1,4-DCB	kg 1,4-DCB	kg 1,4-DCB	kg 1,4-DCB	kg 1,4-DCB	m^2a crop eq	kg Cu eq	kg oil eq	m^3
BAU UK 2022	3.40E+02	6.54E+01	6.51E+05	2.70E+02	5.47E+05	3.29E+00	3.97E+00	8.16E+01	1.01E+01
LCE1	3.51E+02	6.54E+01	6.51E+05	2.70E+02	5.47E+05	5.23E+00	4.00E+00	6.89E+01	1.02E+01
LCE2	3.49E+02	6.54E+01	6.51E+05	2.70E+02	5.47E+05	5.48E+00	3.99E+00	6.90E+01	1.01E+01
LCE3	3.62E+02	6.56E+01	6.51E+05	2.70E+02	5.47E+05	1.22E+01	4.00E+00	7.32E+01	1.01E+01
LCE4	3.67E+02	6.56E+01	6.51E+05	2.70E+02	5.47E+05	1.51E+01	3.99E+00	7.31E+01	1.01E+01
LCE5	3.65E+02	6.56E+01	6.51E+05	2.70E+02	5.47E+05	1.26E+01	4.00E+00	7.25E+01	1.01E+01

Table 20 Results of the cleaner electricity scenarios for the production of 1 m^3 of concrete (part 1).

Scenario	Global warming potential<br>kg CO_2 eq	Stratospheric ozone depletion<br>kg CFC11 eq	Ionizing radiation<br>kBq Co-60 eq	Ozone formation, Human health<br>kg NOx eq	Fine particulate matter formation<br>kg PM2.5 eq	Ozone formation, Terrestrial ecosystems<br>kg NOx eq	Terrestrial acidification<br>kg SO2 eq	Freshwater eutrophication<br>kg P eq	Marine eutrophication<br>kg N eq
BAU UK 2022	3.36E+02	7.52E-05	9.84E+00	9.93E-01	3.49E-01	1.01E+00	1.11E+00	3.28E-02	1.15E-02
LCE1	3.19E+02	6.80E-05	2.73E+01	9.74E-01	3.42E-01	9.86E-01	1.08E+00	3.19E-02	1.17E-02
LCE2	3.19E+02	6.81E-05	1.36E+01	9.74E-01	3.42E-01	9.86E-01	1.09E+00	3.19E-02	1.15E-02
LCE3	3.26E+02	7.47E-05	7.94E+00	9.83E-01	3.45E-01	9.95E-01	1.09E+00	3.25E-02	1.15E-02
LCE4	3.25E+02	7.60E-05	7.68E+00	9.85E-01	3.45E-01	9.97E-01	1.10E+00	3.24E-02	1.15E-02
LCE5	3.25E+02	7.44E-05	8.25E+00	9.83E-01	3.45E-01	9.95E-01	1.09E+00	3.24E-02	1.15E-02

Table 21 Results of the cleaner electricity scenarios for the production of 1 m^3 of concrete (part 2).

Scenario	Terrestrial ecotoxicity<br>kg 1,4-DCB	Freshwater ecotoxicity<br>kg 1,4-DCB	Marine ecotoxicity<br>kg 1,4-DCB	Human carcinogenic toxicity<br>kg 1,4-DCB	Human non-carcinogenic toxicity<br>kg 1,4-DCB	Land use<br>m^2a crop eq	Mineral resource scarcity<br>kg Cu eq	Fossil resource scarcity<br>kg oil eq	Water consumption<br>m^3
BAU UK 2022	4.37E+02	2.36E+01	2.33E+05	2.15E+02	1.96E+05	2.09E+00	1.48E+00	3.67E+01	6.26E+00
LCE1	4.42E+02	2.36E+01	2.33E+05	2.15E+02	1.96E+05	2.91E+00	1.49E+00	3.13E+01	6.31E+00
LCE2	4.41E+02	2.37E+01	2.33E+05	2.15E+02	1.96E+05	3.02E+00	1.49E+00	3.14E+01	6.26E+00
LCE3	4.47E+02	2.37E+01	2.33E+05	2.16E+02	1.96E+05	5.92E+00	1.49E+00	3.32E+01	6.27E+00
LCE4	4.49E+02	2.37E+01	2.33E+05	2.15E+02	1.96E+05	7.14E+00	1.49E+00	3.31E+01	6.27E+00
LCE5	4.48E+02	2.37E+01	2.33E+05	2.15E+02	1.96E+05	6.08E+00	1.49E+00	3.29E+01	6.26E+00

Table 22 Results of the cleaner electricity scenarios for the production of 1 m^2 of building (part 1).

Scenario	Global warming potential<br>kg CO_2 eq	Stratospheric ozone depletion<br>kg CFC11 eq	Ionizing radiation<br>kBq Co-60 eq	Ozone formation, Human health<br>kg NOx eq	Fine particulate matter formation<br>kg PM2.5 eq	Ozone formation, Terrestrial ecosystems<br>kg NOx eq	Terrestrial acidification<br>kg SO2 eq	Freshwater eutrophication<br>kg P eq	Marine eutrophication<br>kg N eq

BAU UK 2022	1.41E+02	3.16E-05	5.51E+00	3.97E-01	1.64E-01	4.10E-01	4.24E-01	2.77E-02	4.52E-03
LCE1	1.35E+02	2.89E-05	1.18E+01	3.91E-01	1.61E-01	4.03E-01	4.15E-01	2.74E-02	4.60E-03
LCE2	1.35E+02	2.90E-05	6.88E+00	3.90E-01	1.61E-01	4.03E-01	4.15E-01	2.74E-02	4.52E-03
LCE3	1.37E+02	3.14E-05	4.82E+00	3.94E-01	1.62E-01	4.06E-01	4.19E-01	2.76E-02	4.51E-03
LCE4	1.37E+02	3.18E-05	4.73E+00	3.94E-01	1.62E-01	4.07E-01	4.19E-01	2.76E-02	4.51E-03
LCE5	1.37E+02	3.13E-05	4.93E+00	3.94E-01	1.62E-01	4.07E-01	4.19E-01	2.76E-02	4.51E-03

Table 23 Results of the cleaner electricity scenarios for the production of 1 m^2 of building (part 2).

	**Terrestrial ecotoxicity**	**Freshwater ecotoxicity**	**Marine ecotoxicity**	**Human carcinogenic toxicity**	**Human non-carcinogenic toxicity**	**Land use**	**Mineral resource scarcity**	**Fossil resource scarcity**	**Water consumption**
**Scenario**	kg 1,4-DCB	kg 1,4-DCB	kg 1,4-DCB	kg 1,4-DCB	kg 1,4-DCB	m^2a crop eq	kg Cu eq	kg oil eq	m^3
BAU UK 2022	2.61E+02	1.03E+01	7.01E+04	1.72E+02	5.89E+04	1.36E+00	1.56E+00	2.10E+01	2.29E+00
LCE1	2.62E+02	1.03E+01	7.01E+04	1.72E+02	5.89E+04	1.65E+00	1.57E+00	1.90E+01	2.31E+00
LCE2	2.62E+02	1.04E+01	7.01E+04	1.72E+02	5.89E+04	1.69E+00	1.57E+00	1.90E+01	2.29E+00
LCE3	2.64E+02	1.04E+01	7.01E+04	1.72E+02	5.89E+04	2.74E+00	1.57E+00	1.97E+01	2.29E+00
LCE4	2.65E+02	1.04E+01	7.01E+04	1.72E+02	5.89E+04	3.18E+00	1.57E+00	1.97E+01	2.29E+00
LCE5	2.64E+02	1.04E+01	7.01E+04	1.72E+02	5.89E+04	2.80E+00	1.57E+00	1.96E+01	2.29E+00

Table 24 Results of the cleaner fuels scenarios for the production of 1 ton of cement (part 1).

	**Global warming potential**	**Stratospheric ozone depletion**	**Ionizing radiation**	**Ozone formation, Human health**	**Fine particulate matter formation**	**Ozone formation, Terrestrial ecosystems**	**Terrestrial acidification**	**Freshwater eutrophication**	**Marine eutrophication**
**Scenario**	kg CO_2 eq	kg CFC11 eq	kBq Co-60 eq	kg NOx eq	kg PM2.5 eq	kg NOx eq	kg SO2 eq	kg P eq	kg N eq
BAU	8.91E+02	1.63E-04	1.82E+01	2.57E+00	9.19E-01	2.59E+00	3.00E+00	8.63E-02	3.10E-02
Solvent	9.09E+02	1.30E-04	1.90E+01	9.00E-01	3.39E-01	9.25E-01	9.32E-01	2.54E-01	4.20E-02
Biomass	7.10E+02	6.24E-05	1.98E+01	8.08E-01	2.99E-01	8.26E-01	8.35E-01	2.19E-01	3.78E-02
MSW 1	6.99E+02	1.93E-04	1.71E+01	1.54E+00	6.07E-01	1.56E+00	1.42E+00	4.48E-02	2.70E-02

MSW 2	6.24E+02	2.11E-04	1.67E+01	8.82E-01	4.16E-01	9.06E-01	5.70E-01	1.74E-02	2.53E-02
Paper and plastic	6.92E+02	3.72E-04	1.73E+01	1.30E+00	8.23E-01	1.34E+00	6.40E-01	1.25E-02	2.50E-02

Table 25 Results of the cleaner fuels scenarios for the production of 1 ton of cement (part 2).

**Scenario**	**Terrestrial ecotoxicity**<br>kg 1,4-DCB	**Freshwater ecotoxicity**<br>kg 1,4-DCB	**Marine ecotoxicity**<br>kg 1,4-DCB	**Human carcinogenic toxicity**<br>kg 1,4-DCB	**Human non-carcinogenic toxicity**<br>kg 1,4-DCB	**Land use**<br>m^2a crop eq	**Mineral resource scarcity**<br>kg Cu eq	**Fossil resource scarcity**<br>kg oil eq	**Water consumption**<br>m^3
BAU	3.40E+02	6.54E+01	6.51E+05	2.70E+02	5.47E+05	3.29E+00	3.97E+00	8.16E+01	1.01E+01
Solvent	4.48E+02	5.97E+01	5.21E+05	3.52E+02	4.37E+05	2.66E+00	4.04E+00	6.38E+01	8.61E+00
Biomass	3.92E+02	5.92E+01	5.18E+05	2.66E+02	4.35E+05	2.24E+00	3.98E+00	4.95E+01	8.38E+00
MSW 1	3.45E+02	5.91E+01	5.56E+05	2.64E+02	4.66E+05	2.02E+00	3.94E+00	3.78E+01	8.78E+00
MSW 2	3.75E+02	5.96E+01	5.69E+05	2.64E+02	4.76E+05	1.35E+00	3.93E+00	2.20E+01	7.88E+00
Paper and plastic	6.24E+02	6.81E+01	6.49E+05	2.72E+02	5.37E+05	1.23E+00	3.93E+00	1.95E+01	7.71E+00

Table 26 Results of the cleaner transport scenarios for the production of 1 ton of cement (part 1).

**Scenario**	**Global warming potential**<br>kg CO_2 eq	**Stratospheric ozone depletion**<br>kg CFC11 eq	**Ionizing radiation**<br>kBq Co-60 eq	**Ozone formation, Human health**<br>kg NOx eq	**Fine particulate matter formation**<br>kg PM2.5 eq	**Ozone formation, Terrestrial ecosystems**<br>kg NOx eq	**Terrestrial acidification**<br>kg SO2 eq	**Freshwater eutrophication**<br>kg P eq	**Marine eutrophication**<br>kg N eq
BAU (normal truck, short distance)	8.91E+02	1.63E-04	1.82E+01	2.57E+00	9.19E-01	2.59E+00	3.00E+00	8.63E-02	3.10E-02
Normal truck, long distance	9.09E+02	1.77E-04	1.86E+01	2.63E+00	9.36E-01	2.66E+00	3.03E+00	8.78E-02	3.15E-02
BET, short distance	8.85E+02	1.57E-04	1.85E+01	2.55E+00	9.15E-01	2.57E+00	2.99E+00	8.67E-02	3.09E-02
BET, long distance	8.90E+02	1.59E-04	1.95E+01	2.57E+00	9.23E-01	2.59E+00	3.01E+00	8.90E-02	3.13E-02

Table 27 Results of the cleaner transport scenarios for the production of 1 ton of cement (part 2).

Scenario	Terrestrial ecotoxicity<br>kg 1,4-DCB	Freshwater ecotoxicity<br>kg 1,4-DCB	Marine ecotoxicity<br>kg 1,4-DCB	Human carcinogenic toxicity<br>kg 1,4-DCB	Human non-carcinogenic toxicity<br>kg 1,4-DCB	Land use<br>m^2a crop eq	Mineral resource scarcity<br>kg Cu eq	Fossil resource scarcity<br>kg oil eq	Water consumption<br>m^3
BAU (normal truck, short distance)	3.40E+02	6.54E+01	6.51E+05	2.70E+02	5.47E+05	3.29E+00	3.97E+00	8.16E+01	1.01E+01
Normal truck, long distance	6.74E+02	6.59E+01	6.56E+05	3.41E+02	5.51E+05	4.08E+00	4.02E+00	8.78E+01	1.01E+01
BET, short distance	1.95E+02	6.56E+01	6.48E+05	2.35E+02	5.45E+05	3.00E+00	3.98E+00	7.92E+01	1.01E+01
BET, long distance	2.39E+02	6.64E+01	6.48E+05	2.36E+02	5.45E+05	3.20E+00	4.05E+00	8.08E+01	1.01E+01

Table 28 Results of the cleaner transport scenarios for the production of 1 m^3 of concrete (part 1).

Scenario	Global warming potential<br>kg CO_2 eq	Stratospheric ozone depletion<br>kg CFC11 eq	Ionizing radiation<br>kBq Co-60 eq	Ozone formation, Human health<br>kg NOx eq	Fine particulate matter formation<br>kg PM2.5 eq	Ozone formation, Terrestrial ecosystems<br>kg NOx eq	Terrestrial acidification<br>kg SO2 eq	Freshwater eutrophication<br>kg P eq	Marine eutrophication<br>kg N eq
BAU (normal truck, short distance)	3.36E+02	7.52E-05	9.81E+00	9.93E-01	3.49E-01	1.01E+00	1.11E+00	3.28E-02	1.15E-02
Normal truck, long distance	3.73E+02	1.06E-04	1.08E+01	1.12E+00	3.86E-01	1.14E+00	1.19E+00	3.59E-02	1.26E-02
BET, short distance	3.23E+02	6.26E-05	1.04E+01	9.47E-01	3.40E-01	9.58E-01	1.09E+00	3.36E-02	1.13E-02
BET, long distance	3.34E+02	6.82E-05	1.27E+01	9.80E-01	3.58E-01	9.97E-01	1.13E+00	3.84E-02	1.21E-02

Table 29 Results of the cleaner transport scenarios for the production of 1 m^3 of concrete (part 2).

Scenario	Terrestrial ecotoxicity<br>kg 1,4-DCB	Freshwater ecotoxicity<br>kg 1,4-DCB	Marine ecotoxicity<br>kg 1,4-DCB	Human carcinogenic toxicity<br>kg 1,4-DCB	Human non-carcinogenic toxicity<br>kg 1,4-DCB	Land use<br>m^2a crop eq	Mineral resource scarcity<br>kg Cu eq	Fossil resource scarcity<br>kg oil eq	Water consumption<br>m^3
BAU (normal truck, short distance)	4.37E+02	2.36E+01	2.33E+05	2.15E+02	1.96E+05	2.09E+00	1.48E+00	3.67E+01	6.26E+00
Normal truck, long distance	1.14E+03	2.48E+01	2.44E+05	3.65E+02	2.04E+05	3.74E+00	1.59E+00	4.98E+01	6.35E+00
BET, short distance	1.32E+02	2.40E+01	2.28E+05	1.41E+02	1.92E+05	1.47E+00	1.50E+00	3.18E+01	6.26E+00
BET, long distance	2.25E+02	2.58E+01	2.28E+05	1.43E+02	1.92E+05	1.89E+00	1.64E+00	3.52E+01	6.32E+00

Table 30 Results of the cleaner transport scenarios for the production of 1 m^2 of building (part 1).

Scenario	Global warming potential<br>kg CO_2 eq	Stratospheric ozone depletion<br>kg CFC11 eq	Ionizing radiation<br>kBq Co-60 eq	Ozone formation, Human health<br>kg NOx eq	Fine particulate matter formation<br>kg PM2.5 eq	Ozone formation, Terrestrial ecosystems<br>kg NOx eq	Terrestrial acidification<br>kg SO2 eq	Freshwater eutrophication<br>kg P eq	Marine eutrophication<br>kg N eq
BAU (normal truck, short distance)	1.41E+02	3.16E-05	5.51E+00	3.97E-01	1.64E-01	4.10E-01	4.24E-01	2.77E-02	4.52E-03
Normal truck, long distance	1.58E+02	4.64E-05	5.98E+00	4.58E-01	1.81E-01	4.75E-01	4.62E-01	2.92E-02	5.05E-03
BET, short distance	1.34E+02	2.55E-05	5.81E+00	3.75E-01	1.59E-01	3.87E-01	4.15E-01	2.81E-02	4.44E-03
BET, long distance	1.40E+02	2.82E-05	6.90E+00	3.91E-01	1.68E-01	4.06E-01	4.34E-01	3.04E-02	4.81E-03

Table 31 Results of the cleaner transport scenarios for the production of 1 m^2 of building (part 2).

Scenario	Terrestrial ecotoxicity<br>kg 1,4-DCB	Freshwater ecotoxicity<br>kg 1,4-DCB	Marine ecotoxicity<br>kg 1,4-DCB	Human carcinogenic toxicity<br>kg 1,4-DCB	Human non-carcinogenic toxicity<br>kg 1,4-DCB	Land use<br>m^2a crop eq	Mineral resource scarcity<br>kg Cu eq	Fossil resource scarcity<br>kg oil eq	Water consumption<br>m^3
BAU (normal truck, short distance)	2.61E+02	1.03E+01	7.01E+04	1.72E+02	5.89E+04	1.36E+00	1.56E+00	2.10E+01	2.29E+00
Normal truck, long distance	5.98E+02	1.09E+01	7.52E+04	2.44E+02	6.30E+04	2.15E+00	1.62E+00	2.73E+01	2.33E+00
BET, short distance	1.14E+02	1.05E+01	6.76E+04	1.37E+02	5.69E+04	1.06E+00	1.57E+00	1.86E+01	2.29E+00
BET, long distance	1.59E+02	1.14E+01	6.76E+04	1.37E+02	5.69E+04	1.26E+00	1.64E+00	2.03E+01	2.32E+00

Table 32 Results of the combined scenarios for the production of 1 ton of cement (part 1).

Electricity scenario	Fuel scenario	Transport scenario	Global warming potential<br>kg CO_2 eq	Stratospheric ozone depletion<br>kg CFC11 eq	Ionizing radiation<br>kBq Co-60 eq	Ozone formation, Human health<br>kg NOx eq	Fine particulate matter formation<br>kg PM2.5 eq	Ozone formation, Terrestrial ecosystems<br>kg NOx eq	Terrestrial acidification<br>kg SO2 eq	Freshwater eutrophication<br>kg P eq	Marine eutrophication<br>kg N eq
BAU	BAU	BAU - short	8.91E+02	1.63E-04	1.82E+01	2.57E+00	9.19E-01	2.59E+00	3.00E+00	8.63E-02	3.10E-02
BAU	BAU	BAU - long	9.09E+02	1.77E-04	1.86E+01	2.63E+00	9.36E-01	2.66E+00	3.03E+00	8.78E-02	3.15E-02
LCE1	Biomass	BET - short	6.66E+02	4.01E-05	5.93E+01	7.44E-01	2.78E-01	7.58E-01	7.70E-01	2.17E-01	3.82E-02
LCE1	MSW 1	BET - short	6.55E+02	1.71E-04	5.73E+01	1.47E+00	5.86E-01	1.49E+00	1.35E+00	4.32E-02	2.74E-02
LCE1	MSW 2	BET - short	5.80E+02	1.89E-04	5.61E+01	8.17E-01	3.96E-01	8.39E-01	5.06E-01	1.59E-02	2.57E-02
LCE1	Paper and plastic	BET - short	6.47E+02	3.49E-04	5.84E+01	1.24E+00	8.02E-01	1.28E+00	5.73E-01	1.09E-02	2.54E-02
LCE1	Biomass	BET - long	6.69E+02	4.19E-05	6.25E+01	7.57E-01	2.86E-01	7.74E-01	7.86E-01	2.20E-01	3.86E-02
LCE1	MSW 1	BET - long	6.58E+02	1.73E-04	6.06E+01	1.48E+00	5.94E-01	1.51E+00	1.37E+00	4.54E-02	2.78E-02
LCE1	MSW 2	BET - long	5.84E+02	1.91E-04	5.93E+01	8.31E-01	4.03E-01	8.55E-01	5.22E-01	1.81E-02	2.61E-02
LCE1	Paper and plastic	BET - long	6.51E+02	3.51E-04	6.16E+01	1.25E+00	8.09E-01	1.29E+00	5.89E-01	1.31E-02	2.58E-02
LCE2	Biomass	BET - short	6.66E+02	4.04E-05	2.86E+01	7.43E-01	2.78E-01	7.58E-01	7.72E-01	2.18E-01	3.77E-02

LCE2	MSW 1	BET - short	6.55E+02	1.71E-04	2.61E+01	1.47E+00	5.87E-01	1.49E+00	1.36E+00	4.33E-02	2.69E-02
LCE2	MSW 2	BET - short	5.81E+02	1.89E-04	2.55E+01	8.17E-01	3.96E-01	8.39E-01	5.07E-01	1.60E-02	2.52E-02
LCE2	Paper and plastic	BET - short	6.48E+02	3.49E-04	2.65E+01	1.24E+00	8.02E-01	1.28E+00	5.75E-01	1.10E-02	2.49E-02
LCE2	Biomass	BET - long	6.70E+02	4.22E-05	3.02E+01	7.57E-01	2.86E-01	7.74E-01	7.88E-01	2.20E-01	3.81E-02
LCE2	MSW 1	BET - long	6.59E+02	1.73E-04	2.76E+01	1.48E+00	5.94E-01	1.51E+00	1.37E+00	4.55E-02	2.73E-02
LCE2	MSW 2	BET - long	5.84E+02	1.91E-04	2.71E+01	8.31E-01	4.03E-01	8.55E-01	5.23E-01	1.81E-02	2.56E-02
LCE2	Paper and plastic	BET - long	6.51E+02	3.51E-04	2.81E+01	1.25E+00	8.10E-01	1.29E+00	5.91E-01	1.32E-02	2.53E-02
LCE3	Biomass	BET - short	6.80E+02	5.52E-05	1.58E+01	7.63E-01	2.85E-01	7.79E-01	7.91E-01	2.19E-01	3.77E-02
LCE3	MSW 1	BET - short	6.69E+02	1.86E-04	1.30E+01	1.49E+00	5.93E-01	1.51E+00	1.38E+00	4.45E-02	2.68E-02
LCE3	MSW 2	BET - short	5.95E+02	2.04E-04	1.27E+01	8.37E-01	4.02E-01	8.59E-01	5.26E-01	1.72E-02	2.51E-02
LCE3	Paper and plastic	BET - short	6.62E+02	3.65E-04	1.32E+01	1.26E+00	8.09E-01	1.30E+00	5.95E-01	1.23E-02	2.48E-02
LCE3	Biomass	BET - long	6.84E+02	5.78E-05	1.66E+01	7.77E-01	2.93E-01	7.96E-01	8.09E-01	2.21E-01	3.80E-02
LCE3	MSW 1	BET - long	6.73E+02	1.89E-04	1.39E+01	1.50E+00	6.02E-01	1.53E+00	1.39E+00	4.68E-02	2.72E-02
LCE3	MSW 2	BET - long	5.99E+02	2.07E-04	1.36E+01	8.51E-01	4.10E-01	8.76E-01	5.44E-01	1.94E-02	2.55E-02
LCE3	Paper and plastic	BET - long	6.66E+02	3.67E-04	1.40E+01	1.27E+00	8.17E-01	1.31E+00	6.12E-01	1.45E-02	2.52E-02
LCE4	Biomass	BET - short	6.79E+02	5.82E-05	1.52E+01	7.67E-01	2.86E-01	7.82E-01	7.93E-01	2.19E-01	3.77E-02
LCE4	MSW 1	BET - short	6.69E+02	1.89E-04	1.24E+01	1.49E+00	5.94E-01	1.51E+00	1.38E+00	4.44E-02	2.68E-02
LCE4	MSW 2	BET - short	5.94E+02	2.07E-04	1.21E+01	8.40E-01	4.03E-01	8.63E-01	5.28E-01	1.70E-02	2.51E-02
LCE4	Paper and plastic	BET - short	6.61E+02	3.68E-04	1.26E+01	1.26E+00	8.09E-01	1.30E+00	5.97E-01	1.21E-02	2.48E-02
LCE4	Biomass	BET - long	6.84E+02	6.10E-05	1.60E+01	7.81E-01	2.94E-01	8.00E-01	8.11E-01	2.21E-01	3.80E-02
LCE4	MSW 1	BET - long	6.73E+02	1.92E-04	1.32E+01	1.51E+00	6.02E-01	1.53E+00	1.40E+00	4.66E-02	2.72E-02
LCE4	MSW 2	BET - long	5.98E+02	2.10E-04	1.29E+01	8.55E-01	4.11E-01	8.80E-01	5.46E-01	1.93E-02	2.55E-02
LCE4	Paper and plastic	BET - long	6.65E+02	3.71E-04	1.34E+01	1.28E+00	8.17E-01	1.32E+00	6.15E-01	1.44E-02	2.52E-02
LCE5	Biomass	BET - short	6.78E+02	5.45E-05	1.65E+01	7.64E-01	2.85E-01	7.80E-01	7.92E-01	2.19E-01	3.77E-02
LCE5	MSW 1	BET - short	6.67E+02	1.86E-04	1.37E+01	1.49E+00	5.93E-01	1.51E+00	1.38E+00	4.44E-02	2.68E-02
LCE5	MSW 2	BET - short	5.93E+02	2.04E-04	1.34E+01	8.38E-01	4.02E-01	8.60E-01	5.27E-01	1.70E-02	2.51E-02
LCE5	Paper and plastic	BET - short	6.60E+02	3.64E-04	1.39E+01	1.26E+00	8.09E-01	1.30E+00	5.95E-01	1.22E-02	2.48E-02
LCE5	Biomass	BET - long	6.82E+02	5.71E-05	1.74E+01	7.78E-01	2.93E-01	7.97E-01	8.09E-01	2.21E-01	3.80E-02

LCE5	MSW 1	BET - long	6.71E+02	1.88E-04	1.46E+01	1.51E+00	6.01E-01	1.53E+00	1.39E+00	4.66E-02	2.72E-02
LCE5	MSW 2	BET - long	5.96E+02	2.06E-04	1.43E+01	8.52E-01	4.10E-01	8.77E-01	5.44E-01	1.93E-02	2.55E-02
LCE5	Paper and plastic	BET - long	6.64E+02	3.67E-04	1.48E+01	1.27E+00	8.17E-01	1.31E+00	6.13E-01	1.44E-02	2.52E-02

Table 33 Results of the combined scenarios for the production of 1 ton of cement (part 2).

**Electricity scenario**	**Fuel scenario**	**Transport scenario**	**Terrestrial ecotoxicity** kg 1,4-DCB	**Freshwater ecotoxicity** kg 1,4-DCB	**Marine ecotoxicity** kg 1,4-DCB	**Human carcinogenic toxicity** kg 1,4-DCB	**Human non-carcinogenic toxicity** kg 1,4-DCB	**Land use** m^2a crop eq	**Mineral resource scarcity** kg Cu eq	**Fossil resource scarcity** kg oil eq	**Water consumption** m^3
BAU	BAU	BAU - short	8.91E+02	1.63E-04	1.82E+01	2.57E+00	9.19E-01	2.59E+00	3.00E+00	8.63E-02	3.10E-02
BAU	BAU	BAU - long	9.09E+02	1.77E-04	1.86E+01	2.63E+00	9.36E-01	2.66E+00	3.03E+00	8.78E-02	3.15E-02
LCE1	Biomass	BET - short	6.66E+02	4.01E-05	5.93E+01	7.44E-01	2.78E-01	7.58E-01	7.70E-01	2.17E-01	3.82E-02
LCE1	MSW 1	BET - short	6.55E+02	1.71E-04	5.73E+01	1.47E+00	5.86E-01	1.49E+00	1.35E+00	4.32E-02	2.74E-02
LCE1	MSW 2	BET - short	5.80E+02	1.89E-04	5.61E+01	8.17E-01	3.96E-01	8.39E-01	5.06E-01	1.59E-02	2.57E-02
LCE1	Paper and plastic	BET - short	6.47E+02	3.49E-04	5.84E+01	1.24E+00	8.02E-01	1.28E+00	5.73E-01	1.09E-02	2.54E-02
LCE1	Biomass	BET - long	6.69E+02	4.19E-05	6.25E+01	7.57E-01	2.86E-01	7.74E-01	7.86E-01	2.20E-01	3.86E-02
LCE1	MSW 1	BET - long	6.58E+02	1.73E-04	6.06E+01	1.48E+00	5.94E-01	1.51E+00	1.37E+00	4.54E-02	2.78E-02
LCE1	MSW 2	BET - long	5.84E+02	1.91E-04	5.93E+01	8.31E-01	4.03E-01	8.55E-01	5.22E-01	1.81E-02	2.61E-02
LCE1	Paper and plastic	BET - long	6.51E+02	3.51E-04	6.16E+01	1.25E+00	8.09E-01	1.29E+00	5.89E-01	1.31E-02	2.58E-02
LCE2	Biomass	BET - short	6.66E+02	4.04E-05	2.86E+01	7.43E-01	2.78E-01	7.58E-01	7.72E-01	2.18E-01	3.77E-02
LCE2	MSW 1	BET - short	6.55E+02	1.71E-04	2.61E+01	1.47E+00	5.87E-01	1.49E+00	1.36E+00	4.33E-02	2.69E-02
LCE2	MSW 2	BET - short	5.81E+02	1.89E-04	2.55E+01	8.17E-01	3.96E-01	8.39E-01	5.07E-01	1.60E-02	2.52E-02
LCE2	Paper and plastic	BET - short	6.48E+02	3.49E-04	2.65E+01	1.24E+00	8.02E-01	1.28E+00	5.75E-01	1.10E-02	2.49E-02
LCE2	Biomass	BET - long	6.70E+02	4.22E-05	3.02E+01	7.57E-01	2.86E-01	7.74E-01	7.88E-01	2.20E-01	3.81E-02
LCE2	MSW 1	BET - long	6.59E+02	1.73E-04	2.76E+01	1.48E+00	5.94E-01	1.51E+00	1.37E+00	4.55E-02	2.73E-02
LCE2	MSW 2	BET - long	5.84E+02	1.91E-04	2.71E+01	8.31E-01	4.03E-01	8.55E-01	5.23E-01	1.81E-02	2.56E-02
LCE2	Paper and plastic	BET - long	6.51E+02	3.51E-04	2.81E+01	1.25E+00	8.10E-01	1.29E+00	5.91E-01	1.32E-02	2.53E-02

LCE3	Biomass	BET - short	6.80E+02	5.52E-05	1.58E+01	7.63E-01	2.85E-01	7.79E-01	7.91E-01	2.19E-01	3.77E-02
LCE3	MSW 1	BET - short	6.69E+02	1.86E-04	1.30E+01	1.49E+00	5.93E-01	1.51E+00	1.38E+00	4.45E-02	2.68E-02
LCE3	MSW 2	BET - short	5.95E+02	2.04E-04	1.27E+01	8.37E-01	4.02E-01	8.59E-01	5.26E-01	1.72E-02	2.51E-02
LCE3	Paper and plastic	BET - short	6.62E+02	3.65E-04	1.32E+01	1.26E+00	8.09E-01	1.30E+00	5.95E-01	1.23E-02	2.48E-02
LCE3	Biomass	BET - long	6.84E+02	5.78E-05	1.66E+01	7.77E-01	2.93E-01	7.96E-01	8.09E-01	2.21E-01	3.80E-02
LCE3	MSW 1	BET - long	6.73E+02	1.89E-04	1.39E+01	1.50E+00	6.02E-01	1.53E+00	1.39E+00	4.68E-02	2.72E-02
LCE3	MSW 2	BET - long	5.99E+02	2.07E-04	1.36E+01	8.51E-01	4.10E-01	8.76E-01	5.44E-01	1.94E-02	2.55E-02
LCE3	Paper and plastic	BET - long	6.66E+02	3.67E-04	1.40E+01	1.27E+00	8.17E-01	1.31E+00	6.12E-01	1.45E-02	2.52E-02
LCE4	Biomass	BET - short	6.79E+02	5.82E-05	1.52E+01	7.67E-01	2.86E-01	7.82E-01	7.93E-01	2.19E-01	3.77E-02
LCE4	MSW 1	BET - short	6.69E+02	1.89E-04	1.24E+01	1.49E+00	5.94E-01	1.51E+00	1.38E+00	4.44E-02	2.68E-02
LCE4	MSW 2	BET - short	5.94E+02	2.07E-04	1.21E+01	8.40E-01	4.03E-01	8.63E-01	5.28E-01	1.70E-02	2.51E-02
LCE4	Paper and plastic	BET - short	6.61E+02	3.68E-04	1.26E+01	1.26E+00	8.09E-01	1.30E+00	5.97E-01	1.21E-02	2.48E-02
LCE4	Biomass	BET - long	6.84E+02	6.10E-05	1.60E+01	7.81E-01	2.94E-01	8.00E-01	8.11E-01	2.21E-01	3.80E-02
LCE4	MSW 1	BET - long	6.73E+02	1.92E-04	1.32E+01	1.51E+00	6.02E-01	1.53E+00	1.40E+00	4.66E-02	2.72E-02
LCE4	MSW 2	BET - long	5.98E+02	2.10E-04	1.29E+01	8.55E-01	4.11E-01	8.80E-01	5.46E-01	1.93E-02	2.55E-02
LCE4	Paper and plastic	BET - long	6.65E+02	3.71E-04	1.34E+01	1.28E+00	8.17E-01	1.32E+00	6.15E-01	1.44E-02	2.52E-02
LCE5	Biomass	BET - short	6.78E+02	5.45E-05	1.65E+01	7.64E-01	2.85E-01	7.80E-01	7.92E-01	2.19E-01	3.77E-02
LCE5	MSW 1	BET - short	6.67E+02	1.86E-04	1.37E+01	1.49E+00	5.93E-01	1.51E+00	1.38E+00	4.44E-02	2.68E-02
LCE5	MSW 2	BET - short	5.93E+02	2.04E-04	1.34E+01	8.38E-01	4.02E-01	8.60E-01	5.27E-01	1.70E-02	2.51E-02
LCE5	Paper and plastic	BET - short	6.60E+02	3.64E-04	1.39E+01	1.26E+00	8.09E-01	1.30E+00	5.95E-01	1.22E-02	2.48E-02
LCE5	Biomass	BET - long	6.82E+02	5.71E-05	1.74E+01	7.78E-01	2.93E-01	7.97E-01	8.09E-01	2.21E-01	3.80E-02
LCE5	MSW 1	BET - long	6.71E+02	1.88E-04	1.46E+01	1.51E+00	6.01E-01	1.53E+00	1.39E+00	4.66E-02	2.72E-02
LCE5	MSW 2	BET - long	5.96E+02	2.06E-04	1.43E+01	8.52E-01	4.10E-01	8.77E-01	5.44E-01	1.93E-02	2.55E-02
LCE5	Paper and plastic	BET - long	6.64E+02	3.67E-04	1.48E+01	1.27E+00	8.17E-01	1.31E+00	6.13E-01	1.44E-02	2.52E-02

Table 34 Results of the combined scenarios for the production of 1 m^3 of concrete (part 1).

Electricity scenario	Fuel scenario	Transport scenario	Global warming potential<br>kg CO_2 eq	Stratospheric ozone depletion<br>kg CFC11 eq	Ionizing radiation<br>kBq Co-60 eq	Ozone formation, Human health<br>kg NOx eq	Fine particulate matter formation<br>kg PM2.5 eq	Ozone formation, Terrestrial ecosystems<br>kg NOx eq	Terrestrial acidification<br>kg SO2 eq	Freshwater eutrophication<br>kg P eq	Marine eutrophication<br>kg N eq
BAU	BAU	BAU - short	3.36E+02	7.52E-05	9.81E+00	9.93E-01	3.49E-01	1.01E+00	1.11E+00	3.28E-02	1.15E-02
BAU	BAU	BAU - long	3.73E+02	1.06E-04	1.08E+01	1.12E+00	3.86E-01	1.14E+00	1.19E+00	3.59E-02	1.26E-02
LCE1	Biomass	BET - short	2.41E+02	1.98E-05	2.98E+01	3.09E-01	1.15E-01	3.18E-01	3.08E-01	7.91E-02	1.39E-02
LCE1	MSW 1	BET - short	2.38E+02	6.55E-05	2.91E+01	5.64E-01	2.23E-01	5.74E-01	5.12E-01	1.81E-02	1.02E-02
LCE1	MSW 2	BET - short	2.12E+02	7.19E-05	2.87E+01	3.35E-01	1.56E-01	3.46E-01	2.15E-01	8.55E-03	9.56E-03
LCE1	Paper and plastic	BET - short	2.35E+02	1.28E-04	2.95E+01	4.82E-01	2.98E-01	4.99E-01	2.39E-01	6.82E-03	9.46E-03
LCE1	Biomass	BET - long	2.48E+02	2.35E-05	3.66E+01	3.37E-01	1.31E-01	3.52E-01	3.42E-01	8.37E-02	1.48E-02
LCE1	MSW 1	BET - long	2.44E+02	6.93E-05	3.59E+01	5.92E-01	2.39E-01	6.08E-01	5.46E-01	2.27E-02	1.10E-02
LCE1	MSW 2	BET - long	2.18E+02	7.57E-05	3.55E+01	3.63E-01	1.72E-01	3.80E-01	2.49E-01	1.31E-02	1.04E-02
LCE1	Paper and plastic	BET - long	2.42E+02	1.32E-04	3.63E+01	5.10E-01	3.15E-01	5.32E-01	2.73E-01	1.14E-02	1.03E-02
LCE2	Biomass	BET - short	2.42E+02	1.99E-05	1.51E+01	3.09E-01	1.15E-01	3.18E-01	3.08E-01	7.91E-02	1.37E-02
LCE2	MSW 1	BET - short	2.38E+02	6.57E-05	1.42E+01	5.64E-01	2.23E-01	5.74E-01	5.13E-01	1.82E-02	9.92E-03
LCE2	MSW 2	BET - short	2.12E+02	7.21E-05	1.40E+01	3.35E-01	1.56E-01	3.46E-01	2.16E-01	8.59E-03	9.33E-03
LCE2	Paper and plastic	BET - short	2.35E+02	1.28E-04	1.44E+01	4.82E-01	2.98E-01	4.99E-01	2.39E-01	6.86E-03	9.22E-03
LCE2	Biomass	BET - long	2.49E+02	2.37E-05	1.84E+01	3.37E-01	1.32E-01	3.51E-01	3.42E-01	8.37E-02	1.45E-02
LCE2	MSW 1	BET - long	2.45E+02	6.95E-05	1.75E+01	5.92E-01	2.39E-01	6.08E-01	5.47E-01	2.27E-02	1.07E-02
LCE2	MSW 2	BET - long	2.19E+02	7.59E-05	1.73E+01	3.63E-01	1.73E-01	3.80E-01	2.50E-01	1.32E-02	1.01E-02
LCE2	Paper and plastic	BET - long	2.42E+02	1.32E-04	1.76E+01	5.10E-01	3.15E-01	5.32E-01	2.73E-01	1.15E-02	1.00E-02
LCE3	Biomass	BET - short	2.48E+02	2.70E-05	8.98E+00	3.18E-01	1.18E-01	3.28E-01	3.18E-01	7.97E-02	1.37E-02
LCE3	MSW 1	BET - short	2.44E+02	7.29E-05	8.01E+00	5.73E-01	2.26E-01	5.84E-01	5.22E-01	1.87E-02	9.89E-03
LCE3	MSW 2	BET - short	2.18E+02	7.92E-05	7.91E+00	3.44E-01	1.59E-01	3.56E-01	2.25E-01	9.17E-03	9.30E-03
LCE3	Paper and plastic	BET - short	2.42E+02	1.35E-04	8.08E+00	4.92E-01	3.02E-01	5.09E-01	2.49E-01	7.46E-03	9.18E-03
LCE3	Biomass	BET - long	2.57E+02	3.25E-05	1.08E+01	3.49E-01	1.35E-01	3.64E-01	3.54E-01	8.44E-02	1.45E-02
LCE3	MSW 1	BET - long	2.53E+02	7.84E-05	9.78E+00	6.03E-01	2.43E-01	6.20E-01	5.59E-01	2.35E-02	1.07E-02

LCE3	MSW 2	BET - long	2.27E+02	8.47E-05	9.68E+00	3.75E-01	1.77E-01	3.92E-01	2.61E-01	1.39E-02	1.01E-02
LCE3	Paper and plastic	BET - long	2.51E+02	1.41E-04	9.84E+00	5.22E-01	3.19E-01	5.45E-01	2.85E-01	1.22E-02	9.96E-03
LCE4	Biomass	BET - short	2.48E+02	2.85E-05	8.70E+00	3.20E-01	1.19E-01	3.29E-01	3.19E-01	7.96E-02	1.37E-02
LCE4	MSW 1	BET - short	2.44E+02	7.43E-05	7.73E+00	5.75E-01	2.26E-01	5.86E-01	5.23E-01	1.87E-02	9.89E-03
LCE4	MSW 2	BET - short	2.18E+02	8.06E-05	7.63E+00	3.46E-01	1.60E-01	3.58E-01	2.26E-01	9.10E-03	9.30E-03
LCE4	Paper and plastic	BET - short	2.42E+02	1.37E-04	7.79E+00	4.94E-01	3.02E-01	5.11E-01	2.50E-01	7.39E-03	9.18E-03
LCE4	Biomass	BET - long	2.56E+02	3.43E-05	1.04E+01	3.51E-01	1.36E-01	3.66E-01	3.55E-01	8.44E-02	1.45E-02
LCE4	MSW 1	BET - long	2.53E+02	8.02E-05	9.43E+00	6.06E-01	2.44E-01	6.22E-01	5.60E-01	2.34E-02	1.07E-02
LCE4	MSW 2	BET - long	2.26E+02	8.64E-05	9.33E+00	3.77E-01	1.77E-01	3.94E-01	2.63E-01	1.38E-02	1.01E-02
LCE4	Paper and plastic	BET - long	2.50E+02	1.43E-04	9.49E+00	5.24E-01	3.19E-01	5.47E-01	2.87E-01	1.21E-02	9.96E-03
LCE5	Biomass	BET - short	2.47E+02	2.67E-05	9.31E+00	3.19E-01	1.18E-01	3.28E-01	3.18E-01	7.97E-02	1.37E-02
LCE5	MSW 1	BET - short	2.43E+02	7.25E-05	8.34E+00	5.74E-01	2.26E-01	5.84E-01	5.22E-01	1.87E-02	9.89E-03
LCE5	MSW 2	BET - short	2.17E+02	7.88E-05	8.23E+00	3.45E-01	1.59E-01	3.56E-01	2.25E-01	9.11E-03	9.29E-03
LCE5	Paper and plastic	BET - short	2.41E+02	1.35E-04	8.41E+00	4.92E-01	3.02E-01	5.09E-01	2.49E-01	7.40E-03	9.18E-03
LCE5	Biomass	BET - long	2.56E+02	3.21E-05	1.12E+01	3.50E-01	1.35E-01	3.64E-01	3.54E-01	8.44E-02	1.45E-02
LCE5	MSW 1	BET - long	2.52E+02	7.80E-05	1.02E+01	6.04E-01	2.43E-01	6.20E-01	5.59E-01	2.34E-02	1.07E-02
LCE5	MSW 2	BET - long	2.26E+02	8.42E-05	1.01E+01	3.75E-01	1.76E-01	3.92E-01	2.61E-01	1.38E-02	1.01E-02
LCE5	Paper and plastic	BET - long	2.49E+02	1.40E-04	1.03E+01	5.23E-01	3.19E-01	5.45E-01	2.85E-01	1.21E-02	9.96E-03

Table 35 Results of the combined scenarios for the production of 1 m^3 of concrete (part 2).

**Electricity scenario**	**Fuel scenario**	**Transport scenario**	**Terrestrial ecotoxicity**	**Freshwater ecotoxicity**	**Marine ecotoxicity**	**Human carcinogenic toxicity**	**Human non-carcinogenic toxicity**	**Land use**	**Mineral resource scarcity**	**Fossil resource scarcity**	**Water consumption**
			kg 1,4-DCB	kg 1,4-DCB	kg 1,4-DCB	kg 1,4-DCB	kg 1,4-DCB	m^2a crop eq	kg Cu eq	kg oil eq	m^3
BAU	BAU	BAU - short	4.37E+02	2.36E+01	2.33E+05	2.15E+02	1.96E+05	2.09E+00	1.48E+00	3.67E+01	6.26E+00
BAU	BAU	BAU - long	1.14E+03	2.48E+01	2.44E+05	3.65E+02	2.04E+05	3.74E+00	1.59E+00	4.98E+01	6.35E+00
LCE1	Biomass	BET - short	1.56E+02	2.18E+01	1.81E+05	1.40E+02	1.52E+05	2.00E+00	1.51E+00	1.48E+01	5.70E+00

LCE1	MSW 1	BET - short	1.39E+02	2.18E+01	1.95E+05	1.39E+02	1.63E+05	1.93E+00	1.50E+00	1.06E+01	5.84E+00
LCE1	MSW 2	BET - short	1.50E+02	2.20E+01	2.00E+05	1.39E+02	1.67E+05	1.68E+00	1.50E+00	5.18E+00	5.53E+00
LCE1	Paper and plastic	BET - short	2.37E+02	2.49E+01	2.27E+05	1.42E+02	1.88E+05	1.67E+00	1.50E+00	4.10E+00	5.47E+00
LCE1	Biomass	BET - long	2.50E+02	2.37E+01	1.81E+05	1.42E+02	1.52E+05	2.63E+00	1.66E+00	1.68E+01	5.78E+00
LCE1	MSW 1	BET - long	2.33E+02	2.36E+01	1.95E+05	1.41E+02	1.63E+05	2.56E+00	1.65E+00	1.26E+01	5.92E+00
LCE1	MSW 2	BET - long	2.44E+02	2.38E+01	2.00E+05	1.41E+02	1.67E+05	2.32E+00	1.65E+00	7.13E+00	5.60E+00
LCE1	Paper and plastic	BET - long	3.31E+02	2.68E+01	2.27E+05	1.44E+02	1.88E+05	2.30E+00	1.65E+00	6.06E+00	5.55E+00
LCE2	Biomass	BET - short	1.55E+02	2.19E+01	1.81E+05	1.40E+02	1.52E+05	2.11E+00	1.51E+00	1.49E+01	5.65E+00
LCE2	MSW 1	BET - short	1.39E+02	2.18E+01	1.95E+05	1.39E+02	1.63E+05	2.05E+00	1.50E+00	1.07E+01	5.79E+00
LCE2	MSW 2	BET - short	1.49E+02	2.20E+01	2.00E+05	1.39E+02	1.67E+05	1.80E+00	1.49E+00	5.23E+00	5.48E+00
LCE2	Paper and plastic	BET - short	2.36E+02	2.50E+01	2.27E+05	1.42E+02	1.88E+05	1.79E+00	1.49E+00	4.16E+00	5.41E+00
LCE2	Biomass	BET - long	2.49E+02	2.37E+01	1.81E+05	1.42E+02	1.52E+05	2.78E+00	1.66E+00	1.68E+01	5.71E+00
LCE2	MSW 1	BET - long	2.33E+02	2.37E+01	1.95E+05	1.41E+02	1.63E+05	2.71E+00	1.64E+00	1.26E+01	5.86E+00
LCE2	MSW 2	BET - long	2.43E+02	2.39E+01	2.00E+05	1.41E+02	1.67E+05	2.46E+00	1.64E+00	7.20E+00	5.54E+00
LCE2	Paper and plastic	BET - long	3.30E+02	2.68E+01	2.27E+05	1.44E+02	1.88E+05	2.45E+00	1.64E+00	6.13E+00	5.48E+00
LCE3	Biomass	BET - short	1.61E+02	2.19E+01	1.81E+05	1.40E+02	1.52E+05	5.23E+00	1.52E+00	1.68E+01	5.66E+00
LCE3	MSW 1	BET - short	1.45E+02	2.19E+01	1.95E+05	1.40E+02	1.63E+05	5.21E+00	1.50E+00	1.26E+01	5.80E+00
LCE3	MSW 2	BET - short	1.55E+02	2.21E+01	2.00E+05	1.40E+02	1.67E+05	4.92E+00	1.50E+00	7.17E+00	5.48E+00
LCE3	Paper and plastic	BET - short	2.43E+02	2.51E+01	2.27E+05	1.43E+02	1.88E+05	5.00E+00	1.50E+00	6.16E+00	5.42E+00
LCE3	Biomass	BET - long	2.56E+02	2.38E+01	1.81E+05	1.42E+02	1.52E+05	6.65E+00	1.66E+00	1.92E+01	5.73E+00
LCE3	MSW 1	BET - long	2.40E+02	2.38E+01	1.95E+05	1.41E+02	1.63E+05	6.63E+00	1.65E+00	1.51E+01	5.87E+00
LCE3	MSW 2	BET - long	2.50E+02	2.40E+01	2.00E+05	1.41E+02	1.67E+05	6.33E+00	1.65E+00	9.61E+00	5.55E+00
LCE3	Paper and plastic	BET - long	3.38E+02	2.69E+01	2.27E+05	1.44E+02	1.88E+05	6.41E+00	1.65E+00	8.60E+00	5.49E+00
LCE4	Biomass	BET - short	1.63E+02	2.19E+01	1.81E+05	1.40E+02	1.52E+05	6.55E+00	1.51E+00	1.68E+01	5.66E+00
LCE4	MSW 1	BET - short	1.47E+02	2.19E+01	1.95E+05	1.40E+02	1.63E+05	6.55E+00	1.50E+00	1.26E+01	5.80E+00
LCE4	MSW 2	BET - short	1.58E+02	2.21E+01	2.00E+05	1.39E+02	1.67E+05	6.23E+00	1.50E+00	7.13E+00	5.48E+00
LCE4	Paper and plastic	BET - short	2.45E+02	2.51E+01	2.27E+05	1.42E+02	1.88E+05	6.35E+00	1.50E+00	6.11E+00	5.42E+00
LCE4	Biomass	BET - long	2.59E+02	2.38E+01	1.81E+05	1.42E+02	1.52E+05	8.28E+00	1.66E+00	1.92E+01	5.73E+00

LCE4	MSW 1	BET - long	2.43E+02	2.37E+01	1.95E+05	1.41E+02	1.63E+05	8.28E+00	1.65E+00	1.50E+01	5.87E+00
LCE4	MSW 2	BET - long	2.53E+02	2.39E+01	2.00E+05	1.41E+02	1.67E+05	7.96E+00	1.64E+00	9.55E+00	5.55E+00
LCE4	Paper and plastic	BET - long	3.41E+02	2.69E+01	2.27E+05	1.44E+02	1.88E+05	8.08E+00	1.65E+00	8.54E+00	5.49E+00
LCE5	Biomass	BET - short	1.62E+02	2.19E+01	1.81E+05	1.40E+02	1.52E+05	5.41E+00	1.51E+00	1.65E+01	5.65E+00
LCE5	MSW 1	BET - short	1.46E+02	2.19E+01	1.95E+05	1.40E+02	1.63E+05	5.39E+00	1.50E+00	1.23E+01	5.80E+00
LCE5	MSW 2	BET - short	1.56E+02	2.21E+01	2.00E+05	1.40E+02	1.67E+05	5.09E+00	1.50E+00	6.86E+00	5.48E+00
LCE5	Paper and plastic	BET - short	2.44E+02	2.51E+01	2.27E+05	1.43E+02	1.88E+05	5.18E+00	1.50E+00	5.84E+00	5.42E+00
LCE5	Biomass	BET - long	2.57E+02	2.38E+01	1.81E+05	1.42E+02	1.52E+05	6.87E+00	1.66E+00	1.88E+01	5.72E+00
LCE5	MSW 1	BET - long	2.41E+02	2.37E+01	1.95E+05	1.41E+02	1.63E+05	6.85E+00	1.65E+00	1.47E+01	5.86E+00
LCE5	MSW 2	BET - long	2.52E+02	2.39E+01	2.00E+05	1.41E+02	1.67E+05	6.55E+00	1.65E+00	9.22E+00	5.55E+00
LCE5	Paper and plastic	BET - long	3.39E+02	2.69E+01	2.27E+05	1.44E+02	1.88E+05	6.64E+00	1.65E+00	8.20E+00	5.49E+00

Table 36 Results of the combined scenarios for the production of 1 m^2 of building (part 1).

**Electricity scenario**	**Fuel scenario**	**Transport scenario**	**Global warming potential**	**Stratospheric ozone depletion**	**Ionizing radiation**	**Ozone formation, Human health**	**Fine particulate matter formation**	**Ozone formation, Terrestrial ecosystems**	**Terrestrial acidification**	**Freshwater eutrophication**	**Marine eutrophication**
			kg CO_2 eq	kg CFC11 eq	kBq Co-60 eq	kg NOx eq	kg PM2.5 eq	kg NOx eq	kg SO2 eq	kg P eq	kg N eq
BAU	BAU	BAU - short	1.41E+02	3.16E-05	5.51E+00	3.97E-01	1.64E-01	4.10E-01	4.24E-01	2.77E-02	4.52E-03
BAU	BAU	BAU - long	1.58E+02	4.64E-05	5.98E+00	4.58E-01	1.81E-01	4.75E-01	4.62E-01	2.92E-02	5.05E-03
LCE1	Biomass	BET - short	1.09E+02	1.21E-05	1.31E+01	1.84E-01	9.18E-02	1.96E-01	1.81E-01	4.15E-02	5.23E-03
LCE1	MSW 1	BET - short	1.08E+02	2.57E-05	1.29E+01	2.60E-01	1.24E-01	2.72E-01	2.41E-01	2.34E-02	4.11E-03
LCE1	MSW 2	BET - short	9.99E+01	2.76E-05	1.28E+01	1.92E-01	1.04E-01	2.04E-01	1.53E-01	2.06E-02	3.93E-03
LCE1	Paper and plastic	BET - short	1.07E+02	4.42E-05	1.30E+01	2.36E-01	1.46E-01	2.49E-01	1.60E-01	2.01E-02	3.90E-03
LCE1	Biomass	BET - long	1.12E+02	1.39E-05	1.63E+01	1.98E-01	9.97E-02	2.12E-01	1.97E-01	4.37E-02	5.64E-03
LCE1	MSW 1	BET - long	1.11E+02	2.75E-05	1.61E+01	2.73E-01	1.32E-01	2.88E-01	2.58E-01	2.56E-02	4.51E-03
LCE1	MSW 2	BET - long	1.03E+02	2.94E-05	1.60E+01	2.05E-01	1.12E-01	2.20E-01	1.69E-01	2.28E-02	4.34E-03
LCE1	Paper and plastic	BET - long	1.10E+02	4.60E-05	1.63E+01	2.49E-01	1.54E-01	2.66E-01	1.76E-01	2.23E-02	4.31E-03

LCE2	Biomass	BET - short	1.09E+02	1.22E-05	7.53E+00	1.84E-01	9.18E-02	1.96E-01	1.81E-01	4.15E-02	5.14E-03
LCE2	MSW 1	BET - short	1.08E+02	2.58E-05	7.26E+00	2.60E-01	1.24E-01	2.72E-01	2.41E-01	2.34E-02	4.02E-03
LCE2	MSW 2	BET - short	1.00E+02	2.77E-05	7.20E+00	1.92E-01	1.04E-01	2.04E-01	1.53E-01	2.06E-02	3.84E-03
LCE2	Paper and plastic	BET - short	1.07E+02	4.43E-05	7.31E+00	2.36E-01	1.46E-01	2.49E-01	1.60E-01	2.01E-02	3.81E-03
LCE2	Biomass	BET - long	1.12E+02	1.40E-05	9.09E+00	1.98E-01	9.97E-02	2.12E-01	1.97E-01	4.37E-02	5.52E-03
LCE2	MSW 1	BET - long	1.11E+02	2.76E-05	8.82E+00	2.73E-01	1.32E-01	2.88E-01	2.58E-01	2.56E-02	4.40E-03
LCE2	MSW 2	BET - long	1.03E+02	2.95E-05	8.77E+00	2.05E-01	1.12E-01	2.20E-01	1.70E-01	2.28E-02	4.22E-03
LCE2	Paper and plastic	BET - long	1.10E+02	4.61E-05	8.87E+00	2.49E-01	1.54E-01	2.66E-01	1.77E-01	2.23E-02	4.19E-03
LCE3	Biomass	BET - short	1.11E+02	1.49E-05	5.20E+00	1.88E-01	9.30E-02	1.99E-01	1.84E-01	4.17E-02	5.13E-03
LCE3	MSW 1	BET - short	1.10E+02	2.85E-05	4.92E+00	2.63E-01	1.25E-01	2.75E-01	2.45E-01	2.36E-02	4.01E-03
LCE3	MSW 2	BET - short	1.02E+02	3.03E-05	4.88E+00	1.95E-01	1.05E-01	2.08E-01	1.57E-01	2.08E-02	3.83E-03
LCE3	Paper and plastic	BET - short	1.09E+02	4.70E-05	4.93E+00	2.39E-01	1.47E-01	2.53E-01	1.64E-01	2.03E-02	3.80E-03
LCE3	Biomass	BET - long	1.15E+02	1.75E-05	6.05E+00	2.02E-01	1.01E-01	2.17E-01	2.02E-01	4.40E-02	5.51E-03
LCE3	MSW 1	BET - long	1.14E+02	3.11E-05	5.77E+00	2.78E-01	1.33E-01	2.93E-01	2.63E-01	2.59E-02	4.38E-03
LCE3	MSW 2	BET - long	1.07E+02	3.30E-05	5.73E+00	2.10E-01	1.13E-01	2.25E-01	1.74E-01	2.31E-02	4.21E-03
LCE3	Paper and plastic	BET - long	1.14E+02	4.96E-05	5.78E+00	2.54E-01	1.56E-01	2.70E-01	1.82E-01	2.26E-02	4.17E-03
LCE4	Biomass	BET - short	1.11E+02	1.54E-05	5.10E+00	1.88E-01	9.31E-02	2.00E-01	1.85E-01	4.17E-02	5.13E-03
LCE4	MSW 1	BET - short	1.10E+02	2.90E-05	4.81E+00	2.64E-01	1.25E-01	2.76E-01	2.45E-01	2.36E-02	4.01E-03
LCE4	MSW 2	BET - short	1.02E+02	3.09E-05	4.78E+00	1.96E-01	1.05E-01	2.08E-01	1.57E-01	2.08E-02	3.83E-03
LCE4	Paper and plastic	BET - short	1.09E+02	4.76E-05	4.83E+00	2.40E-01	1.47E-01	2.54E-01	1.64E-01	2.03E-02	3.80E-03
LCE4	Biomass	BET - long	1.15E+02	1.82E-05	5.91E+00	2.03E-01	1.01E-01	2.18E-01	2.02E-01	4.40E-02	5.51E-03
LCE4	MSW 1	BET - long	1.14E+02	3.18E-05	5.63E+00	2.79E-01	1.33E-01	2.94E-01	2.63E-01	2.59E-02	4.38E-03
LCE4	MSW 2	BET - long	1.06E+02	3.37E-05	5.60E+00	2.11E-01	1.14E-01	2.26E-01	1.75E-01	2.31E-02	4.21E-03
LCE4	Paper and plastic	BET - long	1.13E+02	5.04E-05	5.64E+00	2.55E-01	1.56E-01	2.71E-01	1.82E-01	2.25E-02	4.17E-03
LCE5	Biomass	BET - short	1.11E+02	1.48E-05	5.33E+00	1.88E-01	9.30E-02	2.00E-01	1.84E-01	4.17E-02	5.13E-03
LCE5	MSW 1	BET - short	1.10E+02	2.84E-05	5.04E+00	2.63E-01	1.25E-01	2.76E-01	2.45E-01	2.36E-02	4.01E-03
LCE5	MSW 2	BET - short	1.02E+02	3.02E-05	5.01E+00	1.96E-01	1.05E-01	2.08E-01	1.57E-01	2.08E-02	3.83E-03
LCE5	Paper and plastic	BET - short	1.09E+02	4.69E-05	5.06E+00	2.39E-01	1.47E-01	2.53E-01	1.64E-01	2.03E-02	3.80E-03

LCE5	Biomass	BET - long	1.15E+02	1.74E-05	6.21E+00	2.03E-01	1.01E-01	2.17E-01	2.02E-01	4.40E-02	5.50E-03
LCE5	MSW 1	BET - long	1.14E+02	3.10E-05	5.93E+00	2.78E-01	1.33E-01	2.93E-01	2.63E-01	2.59E-02	4.38E-03
LCE5	MSW 2	BET - long	1.06E+02	3.28E-05	5.89E+00	2.10E-01	1.13E-01	2.25E-01	1.74E-01	2.31E-02	4.21E-03
LCE5	Paper and plastic	BET - long	1.13E+02	4.95E-05	5.95E+00	2.54E-01	1.56E-01	2.71E-01	1.82E-01	2.26E-02	4.17E-03

Table 37 Results of the combined scenarios for the production of 1 m^2 of building (part 2).

Electricity scenario	Fuel scenario	Transport scenario	Terrestrial ecotoxicity	Freshwater ecotoxicity	Marine ecotoxicity	Human carcinogenic toxicity	Human non-carcinogenic toxicity	Land use	Mineral resource scarcity	Fossil resource scarcity	Water consumption
			kg 1,4-DCB	kg 1,4-DCB	kg 1,4-DCB	kg 1,4-DCB	kg 1,4-DCB	m^{2}a crop eq	kg Cu eq	kg oil eq	m^3
BAU	BAU	BAU - short	2.61E+02	1.03E+01	7.01E+04	1.72E+02	5.89E+04	1.36E+00	1.56E+00	2.10E+01	2.29E+00
BAU	BAU	BAU - long	5.98E+02	1.09E+01	7.52E+04	2.44E+02	6.30E+04	2.15E+00	1.62E+00	2.73E+01	2.33E+00
LCE1	Biomass	BET - short	1.21E+02	9.87E+00	5.38E+04	1.36E+02	4.52E+04	1.29E+00	1.58E+00	1.31E+01	2.13E+00
LCE1	MSW 1	BET - short	1.16E+02	9.85E+00	5.78E+04	1.36E+02	4.84E+04	1.27E+00	1.58E+00	1.18E+01	2.17E+00
LCE1	MSW 2	BET - short	1.20E+02	9.91E+00	5.92E+04	1.36E+02	4.95E+04	1.20E+00	1.57E+00	1.02E+01	2.07E+00
LCE1	Paper and plastic	BET - short	1.45E+02	1.08E+01	6.74E+04	1.37E+02	5.59E+04	1.19E+00	1.57E+00	9.92E+00	2.06E+00
LCE1	Biomass	BET - long	1.66E+02	1.08E+01	5.38E+04	1.37E+02	4.52E+04	1.59E+00	1.65E+00	1.40E+01	2.16E+00
LCE1	MSW 1	BET - long	1.62E+02	1.07E+01	5.78E+04	1.37E+02	4.84E+04	1.57E+00	1.65E+00	1.28E+01	2.21E+00
LCE1	MSW 2	BET - long	1.65E+02	1.08E+01	5.92E+04	1.37E+02	4.95E+04	1.50E+00	1.65E+00	1.12E+01	2.11E+00
LCE1	Paper and plastic	BET - long	1.91E+02	1.17E+01	6.74E+04	1.38E+02	5.59E+04	1.50E+00	1.65E+00	1.09E+01	2.09E+00
LCE2	Biomass	BET - short	1.21E+02	9.89E+00	5.38E+04	1.36E+02	4.52E+04	1.33E+00	1.58E+00	1.31E+01	2.11E+00
LCE2	MSW 1	BET - short	1.16E+02	9.87E+00	5.78E+04	1.36E+02	4.84E+04	1.31E+00	1.57E+00	1.19E+01	2.15E+00
LCE2	MSW 2	BET - short	1.19E+02	9.93E+00	5.92E+04	1.36E+02	4.95E+04	1.24E+00	1.57E+00	1.03E+01	2.06E+00
LCE2	Paper and plastic	BET - short	1.45E+02	1.08E+01	6.74E+04	1.37E+02	5.58E+04	1.24E+00	1.57E+00	9.94E+00	2.04E+00
LCE2	Biomass	BET - long	1.66E+02	1.08E+01	5.38E+04	1.37E+02	4.52E+04	1.65E+00	1.65E+00	1.41E+01	2.14E+00
LCE2	MSW 1	BET - long	1.61E+02	1.08E+01	5.78E+04	1.37E+02	4.84E+04	1.63E+00	1.64E+00	1.28E+01	2.18E+00
LCE2	MSW 2	BET - long	1.64E+02	1.08E+01	5.92E+04	1.37E+02	4.95E+04	1.56E+00	1.64E+00	1.12E+01	2.09E+00

LCE2	Paper and plastic	BET - long	1.90E+02	1.17E+01	6.74E+04	1.38E+02	5.59E+04	1.56E+00	1.64E+00	1.09E+01	2.07E+00
LCE3	Biomass	BET - short	1.23E+02	9.92E+00	5.38E+04	1.36E+02	4.52E+04	2.51E+00	1.58E+00	1.38E+01	2.11E+00
LCE3	MSW 1	BET - short	1.19E+02	9.90E+00	5.78E+04	1.36E+02	4.84E+04	2.51E+00	1.58E+00	1.26E+01	2.15E+00
LCE3	MSW 2	BET - short	1.22E+02	9.96E+00	5.92E+04	1.36E+02	4.95E+04	2.42E+00	1.57E+00	1.10E+01	2.06E+00
LCE3	Paper and plastic	BET - short	1.48E+02	1.08E+01	6.74E+04	1.37E+02	5.59E+04	2.44E+00	1.58E+00	1.07E+01	2.04E+00
LCE3	Biomass	BET - long	1.69E+02	1.08E+01	5.38E+04	1.37E+02	4.52E+04	3.19E+00	1.65E+00	1.50E+01	2.14E+00
LCE3	MSW 1	BET - long	1.64E+02	1.08E+01	5.78E+04	1.37E+02	4.84E+04	3.19E+00	1.65E+00	1.38E+01	2.18E+00
LCE3	MSW 2	BET - long	1.67E+02	1.09E+01	5.92E+04	1.37E+02	4.95E+04	3.10E+00	1.65E+00	1.22E+01	2.09E+00
LCE3	Paper and plastic	BET - long	1.93E+02	1.17E+01	6.74E+04	1.38E+02	5.59E+04	3.12E+00	1.65E+00	1.19E+01	2.07E+00
LCE4	Biomass	BET - short	1.24E+02	9.91E+00	5.38E+04	1.36E+02	4.52E+04	3.01E+00	1.58E+00	1.38E+01	2.11E+00
LCE4	MSW 1	BET - short	1.19E+02	9.90E+00	5.78E+04	1.36E+02	4.84E+04	3.01E+00	1.57E+00	1.26E+01	2.15E+00
LCE4	MSW 2	BET - short	1.23E+02	9.95E+00	5.92E+04	1.36E+02	4.95E+04	2.92E+00	1.57E+00	1.10E+01	2.06E+00
LCE4	Paper and plastic	BET - short	1.48E+02	1.08E+01	6.74E+04	1.37E+02	5.59E+04	2.95E+00	1.57E+00	1.07E+01	2.04E+00
LCE4	Biomass	BET - long	1.70E+02	1.08E+01	5.38E+04	1.37E+02	4.52E+04	3.84E+00	1.65E+00	1.50E+01	2.14E+00
LCE4	MSW 1	BET - long	1.66E+02	1.08E+01	5.78E+04	1.37E+02	4.84E+04	3.84E+00	1.65E+00	1.38E+01	2.18E+00
LCE4	MSW 2	BET - long	1.69E+02	1.09E+01	5.92E+04	1.37E+02	4.95E+04	3.75E+00	1.65E+00	1.21E+01	2.09E+00
LCE4	Paper and plastic	BET - long	1.95E+02	1.17E+01	6.74E+04	1.38E+02	5.59E+04	3.78E+00	1.65E+00	1.18E+01	2.07E+00
LCE5	Biomass	BET - short	1.24E+02	9.91E+00	5.38E+04	1.36E+02	4.52E+04	2.58E+00	1.58E+00	1.37E+01	2.11E+00
LCE5	MSW 1	BET - short	1.19E+02	9.90E+00	5.78E+04	1.36E+02	4.84E+04	2.58E+00	1.57E+00	1.25E+01	2.15E+00
LCE5	MSW 2	BET - short	1.22E+02	9.96E+00	5.92E+04	1.36E+02	4.95E+04	2.49E+00	1.57E+00	1.09E+01	2.06E+00
LCE5	Paper and plastic	BET - short	1.48E+02	1.08E+01	6.74E+04	1.37E+02	5.59E+04	2.51E+00	1.57E+00	1.06E+01	2.04E+00
LCE5	Biomass	BET - long	1.70E+02	1.08E+01	5.38E+04	1.37E+02	4.52E+04	3.28E+00	1.65E+00	1.49E+01	2.14E+00
LCE5	MSW 1	BET - long	1.65E+02	1.08E+01	5.78E+04	1.37E+02	4.84E+04	3.28E+00	1.65E+00	1.36E+01	2.18E+00
LCE5	MSW 2	BET - long	1.68E+02	1.09E+01	5.92E+04	1.37E+02	4.95E+04	3.19E+00	1.65E+00	1.20E+01	2.09E+00
LCE5	Paper and plastic	BET - long	1.94E+02	1.17E+01	6.74E+04	1.38E+02	5.59E+04	3.21E+00	1.65E+00	1.17E+01	2.07E+00

Table 38 Comparison of scenario results with industry roadmap targets

Decarbonisation lever	Industry roadmap target (% CO_2 Reduction)	This study's scenario & result (% CO_2 reduction)	Alignment and context
Decarbonisation of electricity	5% of total CO_2 emissions when decarbonising electricity used at both cement plants and in concrete production (GCCA, 2021).<br>6% from renewable energy use in cement manufacturing (CEMBUREAU, n.d.).<br>4% of cementitious material emissions (MPA, 2020)	The Low-Carbon Electricity (LCE) scenario resulted in a 2.7-4.4% reduction in GWP for cement production.	The findings are consistent with the reduction potential attributed to grid decarbonisation in key industry reports, confirming its role as an important, albeit secondary, lever compared to direct emissions reduction.
Decarbonisation of transport	7% for cementitious materials (MPA, 2020).<br>1.5% of total CO_2 emissions in cement manufacturing by moving to zero-carbon transport modes (CEMBUREAU, n.d.).	The Alternative Low-Emission Transport (ALET) scenario shows GWP reductions of up to 4% for cement, 7% for concrete, and 8% for the building level when using BETs.	The analysis confirms that transport electrification is an effective strategy, with impacts that become more pronounced further down the supply chain. The results fall within the range projected by industry roadmaps.
Fuel switching	16% for Portland cement production (ICE, 2022; MPA, 2020).<br>~30% of total CO_2 for clinker via >90% alternative fuel use (CEMBUREAU, n.d.).	The Alternative Fuel Substitution (AFS) scenario resulted in a 20-30% reduction in GWP for cement production.	The results align closely with, and in some cases exceed, the ambitions of major UK and European roadmaps, confirming that significant fuel substitution is a viable and impactful decarbonisation strategy.

References

Afzal, M., Liu, Y., Cheng, J. C. P., & Gan, V. J. L. (2020). Reinforced concrete structural design optimization: A critical review. *Journal of Cleaner Production*, *260*. https://doi.org/10.1016/j.jclepro.2020.120623

Anderson, J., & Moncaster, A. (2020). Embodied carbon of concrete in buildings, Part 1: analysis of published EPD. *Buildings and Cities*, *1*(1), 198-217. https://doi.org/10.5334/bc.59

Bhatty, J. I., Miller, F. M., & Kosmatka, S. H. (2004). *Innovations in portland cement manufacturing*. Portland Cement Association Skokie, Ill.

Boesch, M. E., & Hellweg, S. (2010). Identifying improvement potentials in cement production with life cycle assessment. *ENVIRONMENTAL SCIENCE & TECHNOLOGY*, *44 23*, 9143-9149.

Boesch, M. E., Koehler, A., & Hellweg, S. (2009). Model for cradle-to-gate life cycle assessment of clinker production [Article]. *Environmental Science and Technology*, *43*(19), 7578-7583. https://doi.org/10.1021/es900036e

Cembureau. (1999). Best Available Techniques for the Cement Industry. *The European Cement Association*.

Cembureau. (n.d.). *Cementing the European Green Deal: Reaching climate neutrality along the cement and concrete value chain by 2050*. https://cembureau.eu/media/kuxd32gi/cembureau-2050-roadmap_final-version_web.pdf

Georgiades, M., Shah, I. H., Steubing, B., Cheeseman, C., & Myers, R. J. (2023). Prospective life cycle assessment of European cement production. *Resources, Conservation and Recycling*, *194*. https://doi.org/10.1016/j.resconrec.2023.106998

Gibbons, O. P., Orr, J. J., Archer-Jones, C., Arnold, W., & Green, D. (2022). How to calculate embodied carbon (2nd edition). In. The Institution of Structural Engineers: IStructE Ltd.

Global Cement and Concrete Association. (2021). *Concrete future: the GCCA 2050 Cement and Concrete Industry Roadmap for Net Zero Concrete*.

Habert, G., Miller, S. A., John, V. M., Provis, J. L., Favier, A., Horvath, A., & Scrivener, K. L. (2020). Environmental impacts and decarbonization strategies in the cement and concrete industries. *Nature Reviews Earth & Environment*, *1*(11), 559-573. https://doi.org/10.1038/s43017-020-0093-3

Institution of Civil Engineers. (2022). *Low Carbon Concrete Routemap: Setting the agenda for a path to net zero*.

Karellas, S., Leontaritis, A. D., Panousis, G., Bellos, E., & Kakaras, E. (2013). Energetic and exergetic analysis of waste heat recovery systems in the cement industry. *Energy*, *58*, 147-156. https://doi.org/10.1016/j.energy.2013.03.097

Marceau, M. L., Nisbet, M. A., & VanGeem, M. G. (2007). *Life Cycle Inventory of Portland Cement Concrete* (Vol. SN3011). Portland Cement Association.

Marinković, S., Josa, I., Braymand, S., & Tošić, N. (2023). Sustainability assessment of recycled aggregate concrete structures: A critical view on the current state-of-knowledge and practice. *STRUCTURAL CONCRETE*, *24*(2), 1956-1979. https://doi.org/10.1002/suco.202201245

Mineral Products Association. (2015). *UK Cement Industry 2050 Greenhouse Gas Strategy*. https://cement.mineralproducts.org/documents/MPA_Cement_2050_Strategy.pdf

MPA The Concrete Centre. (2023). Concrete Industry Sustainability Performance Report. 14th report: 2020 performance data. In.

Müller, A., Harpprecht, C., Sacchi, R., Maes, B., van Sluisveld, M., Daioglou, V., Šavija, B., & Steubing, B. (2024). Decarbonizing the cement industry: Findings from coupling prospective life cycle assessment of clinker with integrated assessment model scenarios. *Journal of Cleaner Production*, *450*. https://doi.org/10.1016/j.jclepro.2024.141884

Petek Gursel, A. (2014). *Life-Cycle Assessment of Concrete: Decision-Support Tool and Case Study Application* University of California, Berkeley].
